# Dissecting miRNA gene repression on single cell level with an advanced fluorescent reporter system

**DOI:** 10.1038/srep45197

**Published:** 2017-03-24

**Authors:** Nicolas Lemus-Diaz, Kai O. Böker, Ignacio Rodriguez-Polo, Michael Mitter, Jasmin Preis, Maximilian Arlt, Jens Gruber

**Affiliations:** 1 Junior Research Group Medical RNA Biology, German Primate Center, Kellnerweg 4, 37077 Göttingen, Germany

## Abstract

Despite major advances on miRNA profiling and target predictions, functional readouts for endogenous miRNAs are limited and frequently lead to contradicting conclusions. Numerous approaches including functional high-throughput and miRISC complex evaluations suggest that the functional miRNAome differs from the predictions based on quantitative sRNA profiling. To resolve the apparent contradiction of expression versus function, we generated and applied a fluorescence reporter gene assay enabling single cell analysis. This approach integrates and adapts a mathematical model for miRNA-driven gene repression. This model predicts three distinct miRNA-groups with unique repression activities (low, mid and high) governed not just by expression levels but also by miRNA/target-binding capability. Here, we demonstrate the feasibility of the system by applying controlled concentrations of synthetic siRNAs and in parallel, altering target-binding capability on corresponding reporter-constructs. Furthermore, we compared miRNA-profiles with the modeled predictions of 29 individual candidates. We demonstrate that expression levels only partially reflect the miRNA function, fitting to the model-projected groups of different activities. Furthermore, we demonstrate that subcellular localization of miRNAs impacts functionality. Our results imply that miRNA profiling alone cannot define their repression activity. The gene regulatory function is a dynamic and complex process beyond a minimalistic conception of “highly expressed equals high repression”.

Gene regulation by miRNAs is triggered by target mRNA degradation, translational repression or destabilization of target mRNAs through de-capping and poly-A removal^1^,^2^,^3^,^4^,^5^,^6^. Multiple studies have revealed changes of the expression level of small non-coding RNAs (sncRNA) including gene regulatory microRNAs (miRNA) in various tissues, cell types and diseases^7^,^8^,^9^,^10^.

Progress in next-generation sequencing introduced numerous datasets for transcript analyses, which are used for diagnosis and prognostics in various diseases, in particular in several cancer types^11^. However, these analyses intuitively and frequently concluded that miRNA repression activity is reflected by its expression pattern and level, and in turn, that a robustly expressed miRNA shall repress its targets with increased efficacy. This is to some extent the case and follows defined modes of action^12^,^13^,^14^. Nevertheless, the proportional relation of miRNA expression and function is not always detectable^15^.

Recently, computational approaches on high throughput data investigated miRNA expression levels relative to their respective target mRNA levels. The results revealed only a weak correlation and suggested that expression levels do not concisely explain the regulatory effects^15^,^16^. Therefore, Ago2-miRNA association in miRISCs rather than the total cellular miRNA content has been proposed as a better predictor for repression activity^15^,^17^,^18^. Even though some miRNAs were found to be enriched in Ago2-complexes, these did not necessarily display increased levels of repression in luciferase reporter assays or qRT-PCR. In addition, not all small RNAs that interacted with Ago2 displayed detectable repression activities^15^,^17^,^18^.

Furthermore, miRNAs with similar cellular concentration, as detected in RNAseq read counts or alternative quantification methods, exhibited a wide range of functional outputs. High-throughput assays for miRNA activity^19^ showed that only the most abundant miRNAs mediated target repression while around 60% of the evaluated miRNAs had no activity. Some highly expressed miRNAs reached only weak regulatory activity, while studies on transgene inhibition by miRNAs suggested that a certain level of miRNA expression is required to obtain a detectable repression^20^,^21^.

The increasing complexity and detailed expression analyses did so far not result in improved functional reporters. Commonly luciferase reporter assays get involved in exhaustive biochemical characterizations^22^,^23^,^24^ but the corresponding experimental outputs only allow observations in whole cell populations, and usually reflect end-point-measurements. The more detailed functional assays that are currently available require multiple experimental steps that make their implementation time consuming and rather expensive.

To generate an easy, reliable and accessible assay for single cell level analysis of miRNA activity, we implemented a single plasmid based dual reporter gene system. Within the construct one fluorescence-encoding gene contains a complementary miRNA-target region within its 3′UTR, while the second fluorescence gene served as a reference. The outputs of various reporter-constructs were integrated with a previously reported mathematical model for miRNA-repression^25^. This model describes miRNA control of gene regulation in terms of binding capability and miRNA expression levels. Combining the new reporter-constructs with the elegant model for data interpretation enabled us to perform a high-resolution analysis of miRNA-induced repression.

We experimentally examined the feasibility of integrating the mathematical model for our single plasmid reporter-system, which is referred to as **Untranslated Trans Assay** (**UTA**), by using a synthetic non-human targeting siRNA. This allowed us to incorporate the experimental validation with the model derived predictions to describe gene regulation on the single cell level. Three repression groups were revealed and supported both by experimental data and modeling, i.e. low, mid and high functional miRNAs, based on the interaction between relative expression and binding capabilities, defined in the model by two parameters *θ* (concentration) and λ(binding), respectively.

To validate the system for endogenous miRNA analysis, we generated small-RNA profiles from HEK293 cells in triplicate and built a read-count based list of 29 miRNAs with high, medium or low expression signatures. Specific complementary targets for the individual candidates were integrated into one of the two different fluorescent proteins on the UTA constructs and used in the subsequent single cell analysis. Our measurements revealed that miRNA-activities match the predicted functional groups, but also indicated that repression activity does not directly correlate with the expression levels of the 29 endogenous miRNAs that have been evaluated.

Furthermore, we showed that UTA system can be applied also in HeLa cells, where the ordinal categorical functional groups (high, mid and low) were also observed. Furthermore, some individual miRNAs displayed their functionality according to their subcellular location, low functional and high expressed miRNA were at least partially located in the nucleus, while some mid expressed but high functional miRNAs were seen mainly cytoplasmic.

With the UTA system we were able to i) validate *λ*-*θ* relations for synthetic siRNAs and corresponding target-reporters with stronger or weaker siRNA-binding sites, ii) estimate this relation from experimental data for endogenous miRNAs and evaluate their overall functionality and iii) show that miRNA expression should be closely evaluated in context with functionality and iv) show that some miRNA’s functionality is independent of their overall expression, but their specific cellular location.

In summary, we introduced a system that enables a detailed evaluation of individual miRNAs via a single sensor construct and combined it with downstream *in vitro* and *in silico* analysis, Utilizing this system we could demonstrate that expression level and functionality do not act proportionally for numerous endogenous human miRNAs. This system could help to improve the understanding of miRNA levels and effects in complex situations, including cancer development and progression or cell differentiation.

## Materials and Methods

### Cell culture

Human HEK293 cells were maintained in DMEM (Dulbecco’s modified Eagle’s Medium) supplemented with 15% FCS and 1% antibiotics solution (Pen/Strep), HeLa cells were maintained in RPMI-1640 medium supplemented with 10% FCS and 1% antibiotics solution (Pen/Strep) cells were maintained at 37 °C and 5% CO_2._

### Cloning

**Figure 1:**
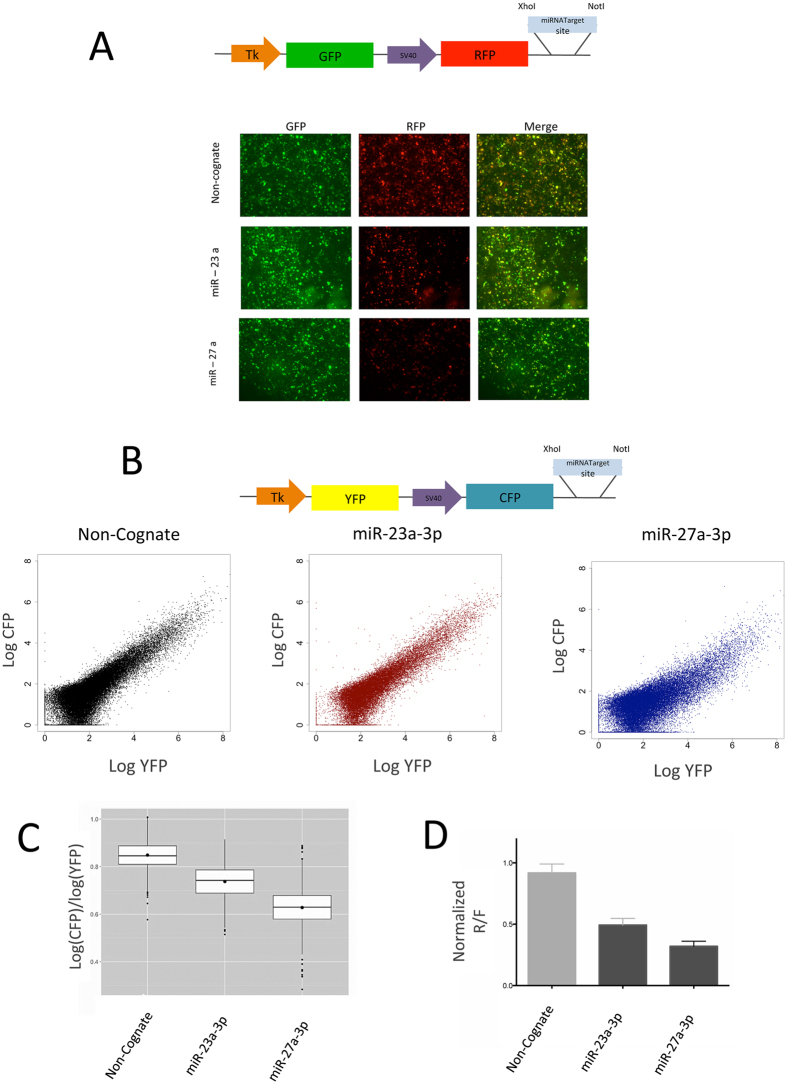
UTA Dual Fluorescence Reporter System functionally characterized miRNA activity. Unstranslated Trans Assay (UTA) uses two independent fluorescent proteins expressed individually from two different promoters. RFP in A (or CFP in B) contained a perfect complementary target region for miRNAs within its 3′ UTR while YFP (or GFP) is unaffected (cartoons depict the used promoters and proteins). Human HEK293 cells were transfected with three different sensor constructs (miR-23a-3p, miR-27a-3p and non-cognate as control) and evaluated after 72 h. (**A**) Qualitative micrographs and (**B**) scatter plots for flow cytometry measurements display constant GFP/YFP expression in all the samples within a broad range of fluorescence intensities. The RFP/CFP expression was reduced for miRNA-23a-3p and miRNA-27a-3p targets but remained proportional to GFP/YFP in the non-cognate control. (**C**) Decreased ratios CFP/YFP for miRNA-targeted constructs from bulk quantitative analysis was performed after flow cytometry. Fluorescence intensity ratios of CFP and YFP for miRNA-23a-3p, miRNA-27a-3p and the non-cognate control were calculated using custom R scripts. (**D**) For standard luciferase assays HEK293 were transfected with miRNA-23a-3p and miRNA-27a-3p targets located within the 3′UTR of *Renilla* luciferase and the ratio between *Renilla* and Firefly luciferase were examined after 72 h.

Plasmids were constructed using key features of the PsiCheck2 (Promega, WI, USA) dual luciferase reporter plasmid: promoters, XhoI and NotI sites were kept and fluorescent (GFP, RFP or CFP) proteins were inserted (Fig. 1A,B). Small RNA sequences were derived from miRBase (www.mirbase.org) and oligonucleotides (Sigma-Aldrich, Munich Germany) were designed with NotI and XhoI overhangs annealed and ligated into fluorescence plasmid and PsiCheck2.

For a complete list of ligated target site oligonucleotides, see the Supplementary Table 1 The fluorescent empty plasmids were deposited in Addgene (82446-82447 empty vector and GL-2 are available).

### Fluorescence microscopy and flow cytometry

HEK293 cells and HeLa (50000 cells/well) were transfected with Lipofectamine 2000 (Thermofisher, MA, USA) or calcium phosphate precipitation method, cells were incubated for 72 h at 37 °C and 5% CO_2_. Fluorescence microscopy pictures were taken using an Axio Observer microscope (Zeiss, Jena, Germany). Exposure time was 90 ms for GFP (Filter BP 525/50) and 900 ms for RFP (Filter BP 605/70).

Plasmid and siRNA-GL2 or Lamin A/C (Dharmacon, CO, USA) were transfected into HEK293 cells (50000 cells/well in 24 well plate) with Lipofectamine 2000 and incubated for 72 h at 37 °C and 5% CO_2_ and subsequently prepared for flow cytometry.

Flow cytometry was performed using a BD LSR II instrument (BD, NJ, USA). The filters employed were 550LP- BP575/26 and BP450/50 for YFP and CFP respectively. YFP positive cells were selected using the FACSdiva™ software and FCS files were exported for analysis in R (see data analysis).

### Luciferase reporter assay

HEK293 cells (50000 cells/well) were transfected with Lipofectamine 2000 (Thermofisher, MA, USA) and incubated for 72 h at 37 °C and 5% CO_2_. Luciferase activities were determined with the Dual-Glo^®^ Luciferase Assay (Promega, WI, USA). psiCHECK-2 transfected cells were treated in accordance with the manufacturer’s protocols and luminescence was assayed on a Synergy 2 microplate reader (Biotek, Winooski, VT, USA). Luminescence intensities for the Renilla luciferase (R_luc_) were normalized against the simultaneously examined firefly luciferase (F_luc_). Normalized R_luc_/F_luc_ values were calculated according to manufacturer’s guidelines.

### RNA isolation and NGS library preparation

Total RNA was isolated from HEK293 and HeLa cells via Phenol/Chloroform extraction according to manufacturer’s protocol (Trizol, Thermo Fisher Scientific, MA, US). The BioAnalyzer System was used to measure the quality and quantity of total RNA. (BioAnalyzer RNA Pico Kit, Agilent, CA, USA). 2 μg of total RNA was used for RNA purification with magnetic bead cleanup module (Life Technologies, CA, USA). Purified RNA was ligated with sequencing adapters, reverse transcribed and purified (Ion Total RNA-Seq Kit v2, Life Technologies, CA, USA). Finally, barcodes were added and library size and amount was detected via BioAnalyzer HS Chip (Agilent CA, USA).

### Next generation sequencing and analysis

Diluted library (18pM) was clonally amplified by emulsion PCR in the IonTorrent OneTouch System according to manufacturer´s protocol (Ion PGM Template OT2 200 Kit, Life Technologies). Amplified library purification was performed by OneTouch ES System. Templated ISPs were loaded onto IonTorrent 316 Chip and sequenced via IonTorrent Personal Genome Machine. Raw reads were trimmed and high quality reads were analyzed via custom Perl scripts as described^10^ and further analyzed by using the R software. Count reads were normalized using a DEseq package^26^ and plots were generated using custom R scripts^27^.

To analyze sRNA contents the Ago2 Co-Immunoprecipitation from HEK293 data was retrieved from the public database GEO: GSE58127^18^; the aligned raw reads for the HEK293 experiments were selected and RPKM values were calculated for plotting.

HeLa cells small RNA data was retrieved from GEO GSE50057^28^, fastq files retrieved and generated were filtered using the Torrent *Suite* Software (Life Technologies, Carlsbad, USA) and high quality reads were then aligned to the miRbase21 and snoRNA base v3 using bowtie2^29^, all the libraries were normalized using RPM (Reads assigned per million mapped reads) and used for plotting.

### Data and statistical analysis

#### Mathematical model

**Figure 2:**
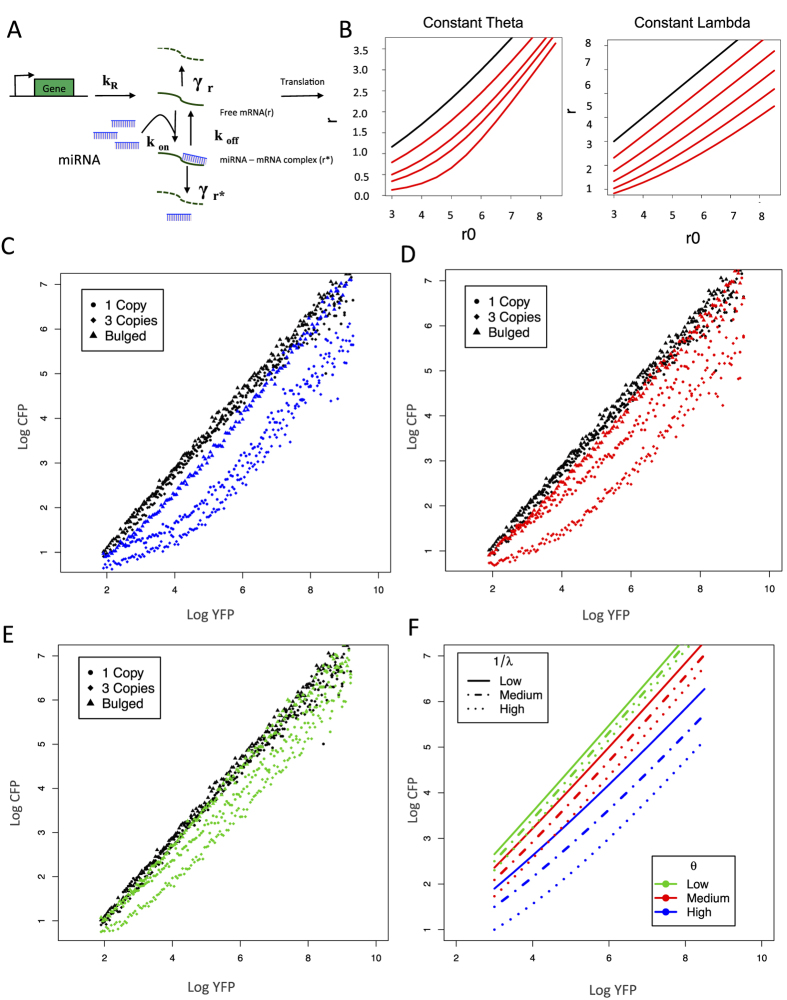
Molecular titration model for miRNA-mediated regulation can be exploited for UTA fluorescent reporters. (**A**) The titration model adapted from (ref. 25 and methods section) describes the steady-state levels of free mRNA (r) and miRNA associated mRNA (r*). The steady state solution for r contains two parameters that define the shape of the function: λ and θ, λ proportional to the effective dissociation constant of miRNA-mRNA (k_off_) and inverse-proportional to the on-rate (k_on_) constant of miRNA-mRNA complex formation while θ is proportional to miRNA concentration. (**B**) Several solutions were simulated using custom R scripts for r as a function of r_0_. Increasing values of θ (left) and λ (right) were utilized for simulations. Only values within the experimentally determined range of YFP intensities were used for r_0_. Control (non-human targeted) siRNA GL-2 reproduced model predictions; three different GL2 target UTA reporters were used to evaluate λ, i.e. bulged with 19 matching complementary base pairs, and 3 unpaired bases (Triangles), one 21 base perfectly complementary target (circles), and three copies of 21 exact complementary bases separated by 4 unpaired bases (diamonds). To mimic effector expression differences (*θ*) synthetic Gl-2 siRNA was co-transfected at different concentration (**C**) 20 nM (**D**) 1.5 nM and (**E**) 0.5 nM. The transfer functions for cells transfected with only the dual reporter (negative control) are depicted in black. (**F**) Simulation of data-derived parameters describes three functional miRNA classes. The GL-2 (siRNA) experimental transfer functions were fitted using non-linear regression methods to define *θ* and λ range; steady state solutions for nine combinations of *θ* (Colors) and 1/λ (lines) are depicted.

We adapted a previously published simple model that explains threshold on miRNA repression activity in terms of miRNAs binding capabilities and miRNA concentration^25^. The model considers the targeted mRNA rate of transcription (k_R_), its decay rate (γ_r_), the free mRNA interaction with miRNAs, which concentration is assumed to be constant, forming mRNA-miRNA complex (r^*^) at on rate (k_on_). This complex can dissociate either to its individual components (k_off_) or degrade the targeted mRNA (γ_r_). These processes are described in the following equations 1, 2, 3, 4 (Fig. 2A):


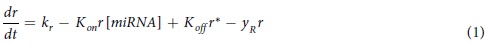



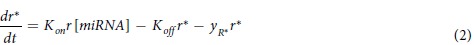


In this context, total miRNA concentration is considered as the free miRNAs plus the miRNAs included in the complex (r^*^):


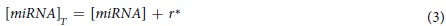


As before^25^ its assumed that no translation is happening from miRNA bound RNA, and only from the free RNA (r). And solving for the steady-state levels of r as before^25^:


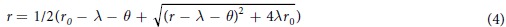


where:


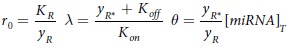


#### Transfer Function generation and parameter estimation

The subset data of YFP-positive cells were extracted on FSC 2.0 or 3.0 and transferred into the R environment using the FlowCore bioconductor package^30^,^31^,^32^. Transfer function generation from raw FACS data was generated as before^25^,^33^,^34^,^35^, in brief YFP Log transformed relative intensities were binned at 0.05 intervals and then the average of relative log CFP intensities was calculated (Supplementary Fig. S1B) transfer function for siRNA experiments or endogenous miRNA on HEK293 cells were used to estimate 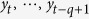 and 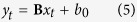 by non-linear (weighted) least squares using equation (4), individual estimates and fit lines for miRNA can be found in Supplementary extended data.

The ranges of YFP intensities were used for simulations and the simulations can be reproduced at https://medicalrnabiology.shinyapps.io/pUTApp/.

#### Other statistical analysis

Graphs were generated using custom scripts with the open source statistical Software R and R-Studio^27^,^36^. Non parametric ANOVA analysis, Dunn’s multiple comparison tests and linear analysis were performed using the PRISM software (GraphPad, CA, USA).

## Results

### The “Untranslated trans Assay” (UTA) fluorescence reporter system characterized miRNA activity and is comparable with luciferase reporter

To test miRNA repression activity at the single cell level we designed two sets of constructs that are: i) suitable for microscopy (Fig. 1A) and ii) accesible to analysis by flow cytometry (Fig. 1B), taking into account the set of excitation and emission filters broadly accessible^37^. We utilized the commonly used dual luciferase assay as a basis, and we introduced two different fluorescent reporters as individual expression units on one plasmid. One of these served as reporter of transcriptional and translational activity while the other contained the exact complementary sequences of a candidate-miRNA, thus serving as a reporter of gene regulatory activity. (Fig. 1).

For using the system in fluorescence microscopy settings, a green fluorescent protein (GFP) was used as gene-expression reference while a red fluorescent protein (RFP) contained a specific complementary target sequence (i.e. non cognate, miR-27a-3p or miR-23a-3p) as reporter for miRNA activity (Fig. 1A). By comparing fluorescence-intensities of the two fluorescence genes the miRNA-affected sites resulted in a reduced expression of RFP when compared to its internal expression reference (GFP), or the non-cognate target control. A mask based tool for evaluation of signal intensities in digital micrographs indicated that the construct design for microscopy enables the analysis of endogenous miRNA inhibitory activity both on the qualitative (Fig. 1A) and on the quantitative level (data not shown).

To quantify the miRNA inhibitory activity on a larger number of cells we developed in parallel constructs that were suited for flow cytometry, including the set of fluorescent proteins that fit the most commonly used laser/filter sets (Fig. 1B). For flow cytometry, the before mentioned construct-design now involved yellow (YFP) and cyan fluorescent proteins (CFP) as expression reference and miRNA reporter, respectively. The YFP intensity and CFP from the non-cognate insert shows a proportional increase between both proteins over the full range of YFP intensities, while for the inserted miRNA target sites the increment of YFP is not accompanied by CFP.

The intensity of CFP for miR-27a-3p is not proportional for low YFP intensities until it reaches high YFP intensities, suggesting a deviation from linearity. In contrast, for miR-23a-3p targeted constructs the CFP/YFP ratio decreased but it keeps a linear behavior (Fig. 1b and Supplementary Fig. S1).

For a direct comparison with luciferase based miRNA-activity analysis, YFP positive cells were gated and the ratio between log (CFP) and log (YFP) was calculated (Fig. 1C). The outcome was directly comparable with the equivalent construct containing Renilla and Firefly luciferases as reference and reporter genes (Fig. 1D). Data were congruent with the microscopy and cytometry observation that the inhibitory capacity of miR-27a-3p is higher than that of miR-23-3p.

### Titration model for miRNA thresholds can be used for UTA System

The output of UTA-experiments after flow cytometry from controls and the two individual miRNA-candidates displayed different results: the empty vector and several non-cognate inserts showed a linear shape relation between reporters (Fig. 1 and Supplementary Fig. S1), miR-23-3p exhibited a small shift of the linear behavior and miR-27a-3p showed a deviation of linearity. This behavior has been characterized as a threshold-linear response for small RNAs in bacteria^38^,^39^ and also for miRNAs^25^.

The advances on miRNA regulation analysis through mathematical models introduced a multilevel understanding of miRNA on an intricate set of nodes, which included targeted expression, feedback positive or negative loops for transcription factors (for review see ref. 40). Furthermore, understanding this multiplicity of interactions generated better algorithms to predict targets of individual miRNAs^41^,^42^. However, this detailed interaction analysis relies mainly on evaluations of expression or predicitions of miRNA – mRNA target interactions and hardly any would be based on a direct functional measurement.

However, the model that described miRNA threshold-linear behaviors in mammalian cells^25^ applied a direct measurement. The experimental layout involved a TET-on controlled bidirectional promoter system with two independent reporters. The approach of Mukkerji and colleagues required a minimum of four experimental steps to be replicated: 1. Generation of stable cell lines that expressed the tetracycline repressor; 2. Characterization of the tetracycline induction kinetics; 3. Transfection of the reporter plasmid; 4. Induction of expression with tetracycline. Since the experimental set up for reporters is comparable, we decided to verify the validity of the model on UTA unique plasmid set up.

The mathematical model describes steady state solutions for free mRNA to translate (r) as function of transcription in absence of miRNA regulation (r0), which here is measured by YFP intensity. The miRNA targeted mRNA transcript (CFP) includes a rate constant (k_on_), unbinding constant (k_off_) and decaying rate (γr*) (Fig. 2A and equations 1, 2, 3, 4 in methods).

Since YFP and CFP are proportional in absence of miRNA repression (Fig. 1B and Supplementary Fig. S1) and the transfection yield a heterogeneous range of fluorescence intensities, we assumed that YFP serves as a transcription-translation sensor an yields r_0_ values.

The steady state solutions for r (CFP) generate two parameters that control the shape of the function and are proportional to binding capabilities and miRNA levels, (*θ*) and (λ). Theta (θ) is proportional to the total miRNA concentration and lambda (λ) to the dissociation constant of the miRNA-mRNA complex (see methods). Using values within the YFP intensities as r_0_, we simulated steady state solutions for several θ and λ, and function shapes suggest that the threshold depends highly on binding capability (1/ λ) while the expression levels (θ) governs the shift of function (Fig. 2B).

To test the validity of the model for the UTA system, we compared a non-endogenous siRNA target with the model predictions. In order to sharpen the threshold (modifying λ: shifting linearity) we introduced either one or three target site-copies, or a bulged target site, to shift the threshold (changing θ) we transfected increasing concentration of siRNA (GL-2) and simplified the resulting FACS outputs through transfer functions (see methods and Supplementary Fig. S1B).

Increasing or decreasing λ by controlling the number of possible pairing bases (bulged, 1 or 3 copies) changes the threshold shape, while increasing concentration by siRNA transfection shift the transfer function away from the non-transfected control (Fig. 2C–E and Supplementary Figs S2 and S3).

Since GL-2 derived functions shape reproduces the model predictions, we simulated 9 different parameter combinations using the GL-2 derived *θ* and λ ranges, the simulation graphs described 3 different functional groups (low functional, mid functional and high functional) according to the shape of the curve (Fig. 2F).

The synthetic exogenous siRNAs transfer functions displayed the experimental threshold shape of the functional groups, where low binding (bulged target site) had no observable threshold but a parallel function shift from the non-transfected control. One and three copies of perfect complementary target sites exhibited threshold differences, for high functional concentrations the curve is non linear, while for mid functional the curve displayed the expected intermediate shape (Fig. 2, and Supplementary Figs S2 and S3).

In model terms, the low functional miRNAs have a low mRNA repression within several λ at low expression levels θ, were no threshold is observed and just a shift on the intercept of the linear behavior is observed. For high and mid functional groups, the function shape depends on 1/λ and θ coupled interaction, because high values for 1/λ could potentially pull medium-expressed miRNAs towards the high functional group (blue), while highly expressed and low 1/λ would rather bring the function close to the mid functional (red) miRNA group (Fig. 2F and supplementary link).

### Inhibitory miRNA activity is disjoined from measured expression

**Figure 3:**
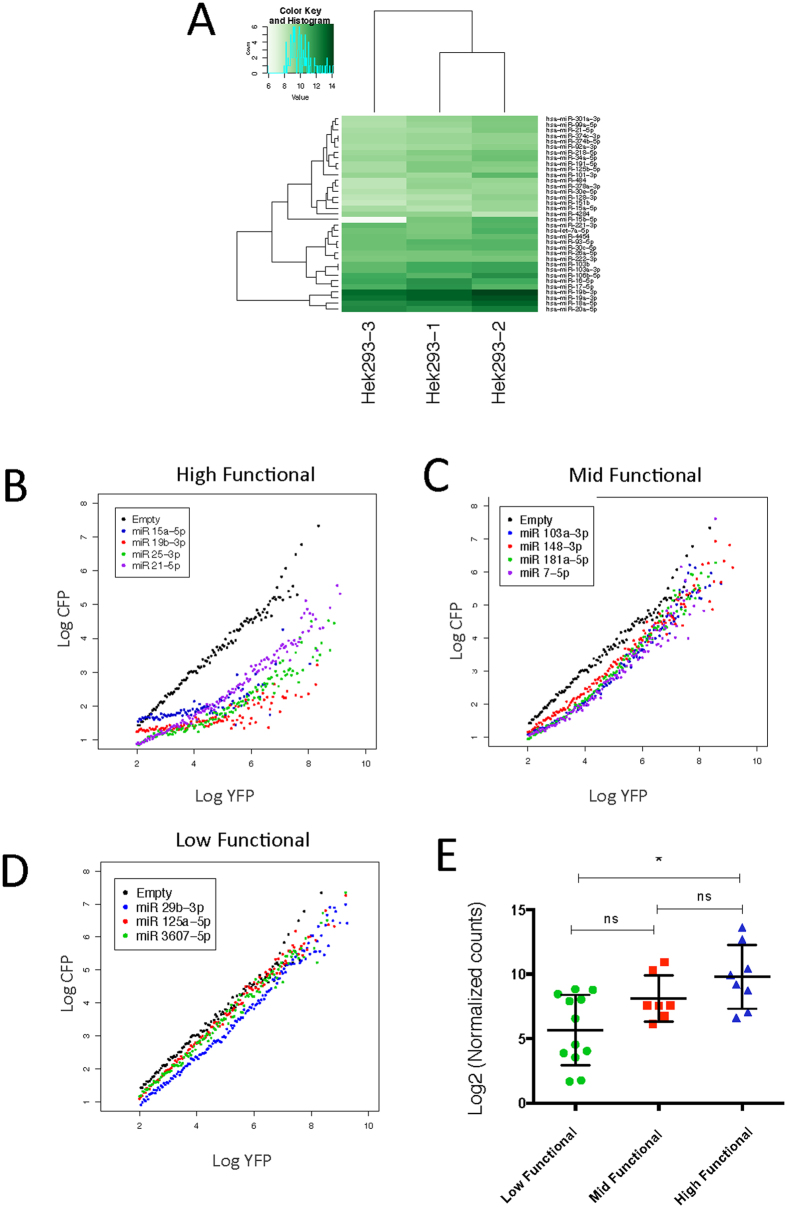
UTA Reporter derived functions uncouple miRNA expression from functionality. (**A**) Expression levels of miRNAs were investigated by NGS using three small-RNA libraries from three independent HEK293 cultures. Sequence data were processed and aligned to sRNA databases, subsequently reads were normalized using DEseq package. Sample quality and sequencing reproducibility were assured by using heat map and blind hierarchal clustering. Selected miRNA target regions were inserted into the CFP 3′UTR, transfected into HEK293 cells and incubated for 72 h, then cells were examined by FACS and transfer functions were calculated (for details see methods section). Three different functional behaviors are depicted according to the shape of the transfer function: (**B**) High functional (Left) have a threshold up to high YFP intensities, (**C**) mid Functional (Middle) have a threshold at intermediate YFP intensities and (**D**) low functional (Right) have no detectable threshold (compare Supplementary Fig. S3). (**E**) Functional groups were used as ordinal variables and mean normalized counts were compared between groups (P < 0.01 Kruskal-Wallis test, Dunns multiple comparisons test * < 0.05).

To test the relation between expressed miRNAs and the UTA measured repression, three independent HEK293 small-RNA libraries were generated and sequenced using the Ion Torrent platform. The microRNAs contained in the miR-Base 21 were selected as a reference dataset and the individual read-counts were normalized using the DEseq package on Bioconductor. The three independent libraries have highly similar read-count distributions and in particular the miRNAs share similar expression profiles (Fig. 3A and Supplementary Fig. S4). We used the average normalized log2 transformed reads to select 3 different miRNAs from quartile ranked data and to test their functionality (Supplementary Fig. S5).

The endogenous miRNA transfer functions displayed the same threshold behavior that was observed on raw flow cytometry data (Fig. 1B and Supplementary Fig. S1). The miRNAs UTA transfer functions show that low expressed miRNA below median have low or no inhibitory capability, while miRNAs over the median and third quartile show a range of behavior that does not reflect their expression level (Supplementary Fig. S5); due to this observation we increased the number of tested miRNA above the median.

Comparing the shape of the curves from endogenous miRNAs with the simulations and the GL-2 experiments, we observed the defined groups (Fig. 2F): First a low functional group where no threshold is observed and just a shift on the intercept of the linear behavior is seen (Fig. 3D). To distinguish whether this change could be caused by the inserted target-sequence and not by an “effective low functional miRNA” we tested four different non-cognate constructs and did not observe any change of the curve shape compared with the control (Supplementary Fig. S1D).

Secondly, high functional miRNAs have an observed transfer function with a notorious threshold (low CFP intensities from a considerable range of YFP intensities), before it reaches a linear behavior (Fig. 3B) and the mid functional where a threshold is observable but the shift to linear is reached already at low YFP intensities (Fig. 3C).

Also, when we compared the mean of transformed read counts among the described functional groups we observed no differences between low-mid and high-mid functional groups but a clear and significant difference between the low and high functional. This finding indicates that differences at inhibitory potential might appear below 1000 reads, reflecting previously discussed expression thresholds seen in bulk luciferase and fluorescence assays^17^,^19^,^20^,^21^. These results also display that the differences on inhibitory potential for expression above the median differ broadly and depend highly on elements that govern the shape of the transfer function (see above).

To examine whether the described groups may rather be reflected by RISC associated miRNAs than by the total miRNA content, we analyzed public available Ago2 co-IP data sets on HEK293 cells^18^ and localized our functionally tested miRNAs. The distribution of our candidates is, in terms of expression levels, comparable between our libraries and other NGS platforms. Interestingly we did not identify any remarkable trend towards enrichment or depletion in Ago2 for our functional groups (Supplementary Fig. S6).

### Titration model for endogenous miRNAs settles expression and functionality discrepancy

**Figure 4:**
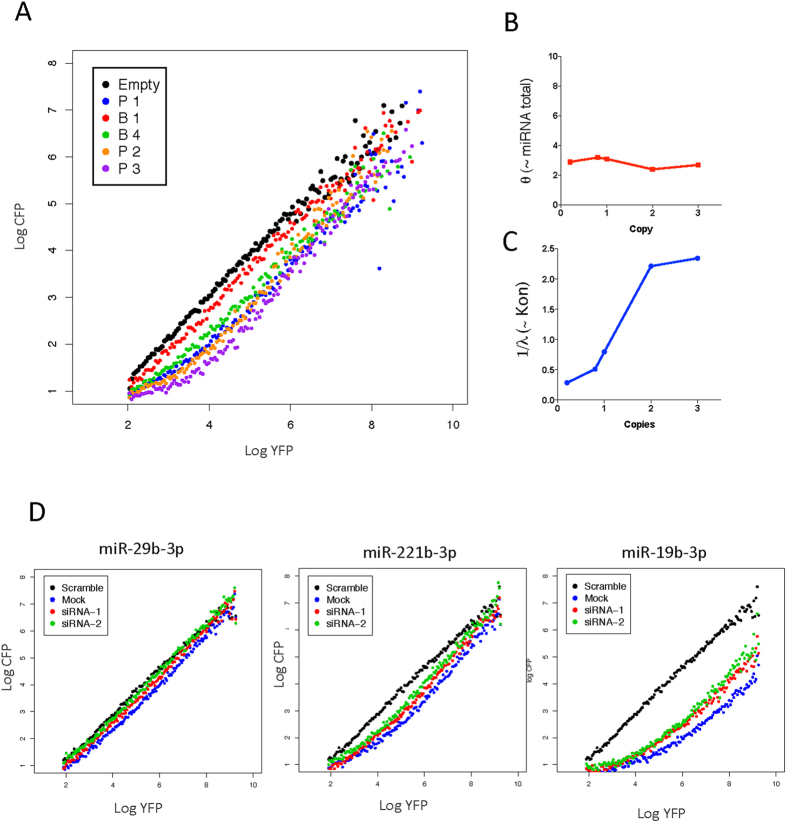
Threshold modulation is suitable for functional evaluation of endogenous miRNAs. Sharpening of the threshold was investigated by target site modulations; bulged and perfect binding sites for miR-27a-3p were inserted at 3′UTR of CFP, HEK293 cells were transfected and analyzed by FACS after 72 h. (**A**) Transfer functions for perfect binding sites (“P”) with 1, 2 and 3 copies and bulged (contain three unmatched bases “B”) sites 1 and 4 copies are shown. Experimentally derived parameters predicted behavior for endogenous miRNA: UTA derived transfer functions were fitted using non-linear least squares using custom R scripts and *θ* and λ values were calculated for different miR-27a-3p transfer functions: (**B**) Copy number of miRNA targets vs. calculated *θ* (~miRNA) and (**C**) Copy number of miRNA targets vs. 1/λ (~Kon), copy number value for bulged target sites was arbitrary set to 0.2. (**D**) Outcompeting miRNA with siRNA shift the UTA resultant function, three-selected UTA construct for low, mid and high functional miRNA were cotransfected with two distinct siRNA and evaluated after 72 h.

Since the GL-2 siRNA experiment proved that UTA out puts are remarkably valid for the model predictions. We decided to define its reliability for endogenous miRNAs we switch the threshold sharpness of miR-27a-3p introducing different copies for perfect match and bulged target sites (Fig. 4A). We observed similar sharpening upon copy number increment, which is also observable for other endogenous miRNA with low and high functionality (Supplementary Fig. S7). Our findings suggest that the repression measurement produced by our reporter analysis can generate an understandable relation between expression levels and binding capacities.

Since the model for miRNA repression activity generates two quantifiable parameters that could be artificially modulated, it also may have the capacity to generate coherent values when the transfer functions are fitted to threshold model. Using the transfer functions from miR-27a-3p threshold shape modulation λ and *θ* were calculated. Remarkably *θ* kept a constant value for perfect and bulged copies (Fig. 4B) reflecting the constant express levels of miR-27a-3p on the experiments, while λ reduces its value with the increased binding capacity (Fig. 4C).

To evaluate a change on θ for endogenous miRNAs, we reason that if an excess of siRNA is introduced it will outcompete endogenous miRNAs, thus reducing the number of effective miRNA molecules. Two independent siRNAs (LMNA and GL2) were co-transfected with UTA reporters for low, mid and high functional endogenous miRNAs. As expected the transfer function shifted upon siRNA co-transfection but no change on the sharpness was observed (Fig. 4A and Supplementary Fig. S7).

**Figure 5:**
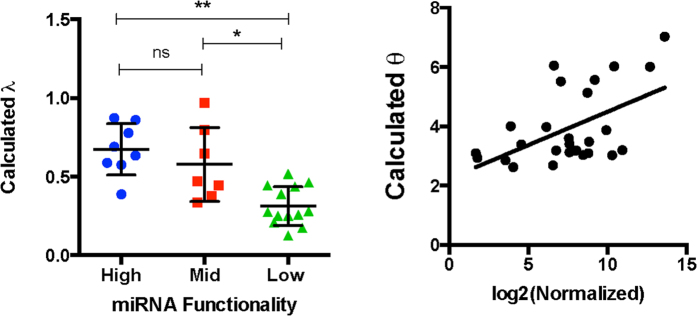
Molecular miRNA titration model describes functional groups and solves discrepancy of expression/repression by incorporating binding capability. UTA transfer functions for tested miRNAs were fitted using non-linear least squares regression and λ and *θ* were extracted (**A**) Calculated 1/λ (~K_on_) plotted versus different functional groups (P < 0.05 Kruskal-Wallis test, * < 0.05 ** < 0.01). (**B**) Expression in mean normalized counts from the three sequenced libraries vs. calculated (~miRNA).

Since the model explanation applies to repression activities of exogenous siRNA and endogenous miRNA and describes repression as an interaction between binding capabilities (λ) and miRNA quantity (*θ*), we derived these parameters for all tested miRNA-candidates and their transfer functions. We observed significant differences between the calculated 1/λ for low functional and the other tested groups, while there was no significant differences among mid and high functional groups (Fig. 5A). Interestingly, we observed that the calculated *θ* and the mean read counts are proportional (Fig. 5B) but without a direct correlation (see discussion).

We can conclude from our data that miRNA functionality depends on a coupled interaction between miRNA binding capabilities and expression levels. For that reason the three functional groups described above might have a broad spectrum for expression and binding capabilities, suggesting that miRNA expression levels from total or RISC associated miRNAs might be a limited tool for inhibitory potential prediction. Direct measurements at single cell level, defined as transfer function, indicated a better description of miRNA function that could be integrated into a more dynamic model for miRNAs.

### miR-103-3p and let-7-c cellular location explain expression and functionality discrepancy in HeLa cells

We considered that the UTA functional outputs together with the molecular titration model explain the discrepancy between miRNA expression and functional repression, showing scenarios where highly expressed miRNA might have no or low function or middle expressed miRNAs show a high functional repression mechanism. These specificities can deliver new hypothesis to define the reason behind this behavior.

miRNAs are located to different sites within a cell, e.g. in cytoplasm or nucleoplasm^19^,^43^,^44^,^45^. This situation potentially changes their mode of activity and thus yielding different behaviors when functionally tested with the UTA reporter assays. In order to test this scenario, we used deep sequenced small RNAs from different subcellular compartments of HeLa cells from the GEO data base^28^,^45^. To assure that the data retrieved and the conclusions we can generate are valid, we sequenced total small RNAs from HeLa cells and performed in parallel the same bioinformatics analysis to generate and compare retrieved fastq files.

**Figure 6:**
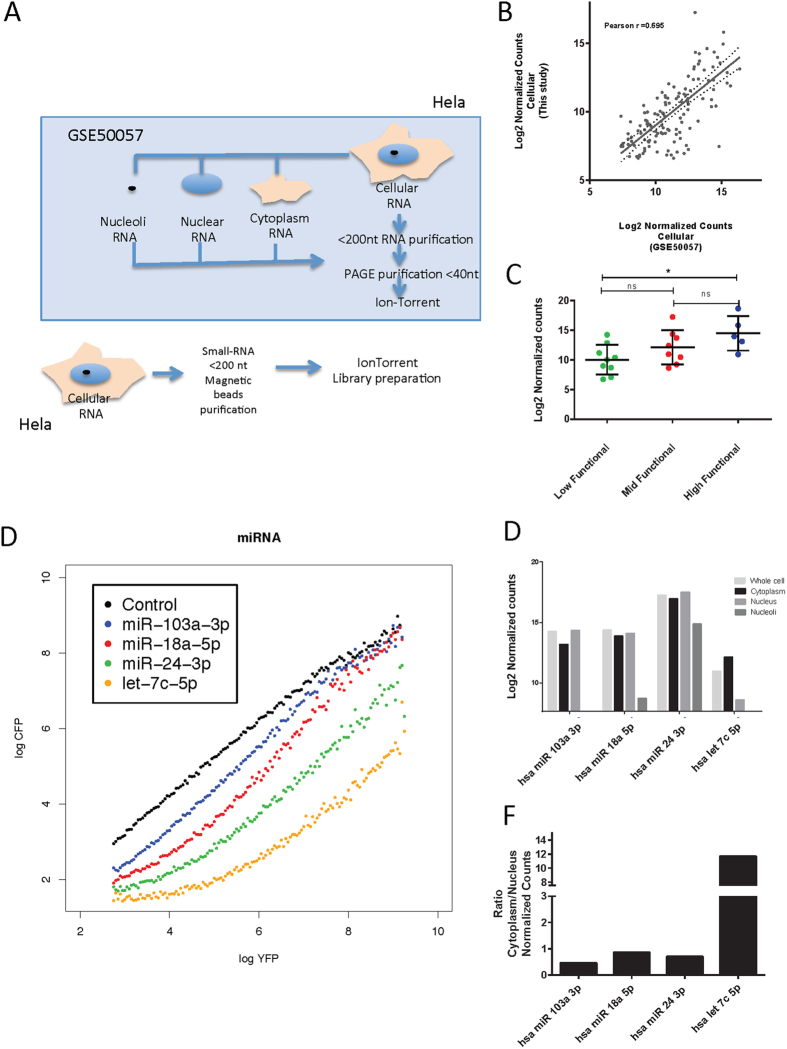
Subcellular localization of miRNAs influences their functional output. GEO50057 retrieved raw data from HeLa small – RNA seq experiment was retrieved and compared with fresh produced total cell small RNA library. Fractionated cell compartment derived small RNA libraries were used for further analysis of nuclear, cytoplasmic and nucleolar content. (**A**) Experimental layout for HeLa small RNA library preparation in GSE50057^28^ and this study. (**B**) Comparison of small RNA expression profiles from HeLa cells from two independent sources, plot of normalized counts HeLa library prepared vs retrieved (r = 0.695, P < 2.2e16 r^2^ = 0.48).(**C**) miRNAs UTA reporters were transfected into HeLa cells, evaluated after 72 h and UTA transfer functions were plotted (Supplementary Fig. S8) distributed into the three ordinal variables as before and cellular normalized counts were compared between groups (P < 0.01 Kruskal-Wallis test, Dunn’s multiple comparisons test * < 0.05). Four individual miRNAs were chosen according to their cellular expression, 3 miRNAs on low and mid functional groups with expression higher than the high functional median (miR-103a-3p, miR-18a-5p, miR-24-3p) and one high functional miRNA lower than the mid functional median of normalized cellular read counts. (**D**) UTA transfer functions (**E**) Bar plots for cellular compartments small RNA libraries and (**F**) Cytoplasmic/Nuclear ratio for selected candidates.

RPM Normalization was performed for each of the libraries used, the overall experimental design between GSE50057 and our produced library differ basically in an extra purification step performed for RNA smaller than 40 nt (Fig. 6A). The total cellular small RNA content from GSE50057 and the generated library display a positive correlation (Fig. 6B), this allowed us to assume that both HeLa cell lines were comparable and that the subcellular location data can be extrapolated to our cells. Moreover, similar correlations were also observed for cytoplasmic and nuclear libraries (Supplementary Fig. S8B).

We transfected UTA constructs sensoring all HEK293 selected miRNAs into HeLa cells and observed similar transfer function shapes. We also observed again the three distinct groups of functionality (Supplementary Fig. S8C) and also no differences on expression between the mid and high functional group (Fig. 6C). Interestingly these differences continued within the cytoplasmic fraction (Supplementary Fig. S8D) and together the data suggest that the UTA system is useful for more cell types and that the functional classification is not limited to HEK293 cells.

After testing the validity of the retrieved libraries and replicated the UTA output for HeLa cells, we selected some candidates from the different functional groups, checked their cellular location and calculated the cytoplasm-nuclear ratio. Highly expressed miRNAs located at mid and low functional groups that were greater or equal to the median expression of high functional miRNAs were selected (miR-103a-3p, miR-18a-5p, miR-24-3p), while one high functional miRNA with expression lower than the median of mid functional miRNAs expression was selected (let-7c-5p).

The UTA transfer functions for candidates were plotted (Fig. 6D). Interestingly, miR-103-3p that is highly expressed but low functional has a low ratio of cytoplasm to nuclear localization. Similar low ratios were observed for miR-18a-5p and miR-24-3p, while let-7c that is high functional but less abundantly expressed is located merely to the cytoplasm as is shown by a high ratio cytoplasm/nucleus.

In summary, our findings and results underline that the UTA construct system is applicable to other cells types. The functional data is useful to define alternative hypothesis based on its outputs, and that miRNAs subcellular location also defines its functionality, displaying that expression profiles without a systematic and detailed functional outputs could be misleading.

## Discussion

Recent studies have deep sequenced miRNAs present in the total small RNA population in various cell types and tissues. The underlying assumption among these studies is that miRNA quantification accurately reflect miRISC activity, since aligned reads from sRNA libraries display Ago2 guide strand stabilization at miRNA genomic loci^46^,^47^, and other structural features that facilitate miRNA discovery^48^. On the other hand Ago2 stability depends highly on miRNA abundance^49^,^50^ validating the postulate that miRNA expression is equivalent to repression activity.

Nevertheless, the complex and dynamic cellular function of miRNAs introduces uncertainty to the expression profiling conclusions. For instance: Ago proteins (Ago 1–4) quantities are lower than those of miRNA molecules, these Ago sub-stoichiometric amounts deal with the miRNA excess by multiple rounds of recruitments on miRNA-mRNA free complex, showing that Ago2 can interact with pre-made double stranded RNA^51^, implying that reads on miRNA profiles may contain also free mRNA-miRNA complexes.

Furthermore, studies of miRISCs sub-cellular locations suggested that only ER associated fractions perform gene silencing at the mRNA level^43^, while others show different subcellular miRNA localization^18^,^19^,^52^. Here we documented that miR-103a-3p is a highly expressed miRNA but displays only low functionality, because it is confined to the nuclear compartment. In contrast, the high-functional miRNA let-7-c with mid expression is located mainly in the cytoplasm (Fig. 6 and Supplementary Table 3).

At functional level, lentiviral based high-throughput methods, which include antibiotic-selection and cell sorting, confirmed the breach silencing-expression^19^,^52^ because more than 50% of expressed miRNAs have no detectable activity. Consequently, some studies proposed to investigate Ago2-associated miRNAs to improve inhibitory activity prediction^17^,^18^. However, miRISC experiments cannot entirely predict miRNA functionality, because Ago2 HITS-Clip described that some highly abundant Ago2-bound small RNAs showed no inhibitory activity^17^.

We reason that a high-resolution functional miRNA evaluation must be included and might bring clarifying evidence, since a great number of studies test the miRNA-functionality through artificial expression or luciferase based assays. These assays could misjudge the miRNA’s physiological activity and provide only whole population measurements within a rather narrow detection limit^17^,^18^,^19^. For that reason, we decided to generate and validate a single plasmid fluorescent reporter, which is called here Un-translated trans assay (UTA), to test systematically miRNA activity at the single cell level and put it in context with expression levels.

To set up a single cell resolution data analysis, we adapted and tested the reliability of a molecular titration model^25^. The published model was set up for only one endogenous miRNA (miR-20a) and involved a TET dependent bidirectional promoter, that requires selection of positive cells in parallel with the exogenous expression of the tetracycline repressor.

We propose that UTA drastically simplifies the experimental workflow and can expand the model to other cell types. It was created with new and established features suitable for all transfectable cell types. Our dual fluorescence system uses common constitutive promoters (Tk and SV40, Fig. 1), and enables qualitative read outs by microscopy (Fig. 1A), quantification by flow cytometry (Fig. 1B) and reproduced the results obtained in luciferase assays for the same miRNA candidates (Fig. 1C).

To utilize the existing model, we adapted the same flow cytometry raw data processing, which generates simple curves called transfer function^25^, (see methods – Supplementary Fig. S1B), to test the feasibility of the mathematical model with the simplified UTA constructs. The mathematical model estimates the two parameters λ and θ that integrate binding capabilities and miRNA expression, respectively. We used the curve outputs from flow cytometry and estimated the parameters without considering any independent K_on_, K_off_, or γ to avoid over-fitting. However, we consider that our system can be integrated with novel single-molecule of Ago complex derived kinetics to investigate miRNAs *in vivo*^53^,^54^,^55^.

Regardless the differences among our fluorescent sensor and the previously reported^25^, our non-human target siRNA data, which modulates the threshold sharpness (through differential binding site utilization) altering λ, and shift the function (increasing siRNA concentration) controlling *θ*, clearly exhibited the same behavior as simulated prediction (Fig. 2). In addition and more convincing, it also applies to endogenous miRNAs (Fig. 4 and Supplementary Fig. S7).

Interestingly, our UTA sensor also detaches miRNAs repression from expression at cellular and miRISC level (Fig. 3 and Supplementary Figs S5 and S6). We observed only significant difference on expression between low and high functional groups; this might correspond to the 1000 reads (~9 log 2 scale) detection threshold for luciferase-assays^17^, suggesting that detectable luciferase outputs are just evident for high functional miRNAs. Furthermore looking at RIP-seq data from HEK293, and located the tested functionality, we did not notice a bias towards high-functional miRNAs in Ago2 complexes (Supplementary Fig. S6), which show that Ago2 bound miRNAs are also not good for functional prediction^17^, we consider that it also clear that a great dynamic network control miRNA functionality. We can not discard that stabilizing mechanism might also changes the functional output we described, for example interaction with GW182^5^, posttranslational Ago2 modifications or location of P bodies^56^,^57^, or even different ratios number of target miRNAs^19^ could also yield our described output.

We considered that the UTA system could be used to test functionally to generate hypothesis for Ago2 modifications, interaction with other molecular participants as GW182, increased number of target. As an example, we tested whether the subcellular location might change the functional output for miRNAs, we used published data from small RNA libraries derived from different cellular compartments; we showed that the clones used are similar (Fig. 6B and Supplementary Fig. S8) to the HeLa clone we tested. Remarkably, HeLa cells show the 3 functional groups defined for HEK293, and that some miRNAs that are low functional are mainly at the nucleus, while other are specifically in the cytoplasm.

Here, we introduced evidence to clarify expression-function detachment. Our reporter system in combination with downstream analysis quantitatively characterized miRNA activity and defined parameters proportional to dissociation constant and relative expression. This is not only empowering quantitative high-resolution analysis of miRNA-functionality, which includes expression levels within its output; it also defines a better picture for miRNA function.

Additionally, we also consider that our tool, which integrates the titration model^25^, could explain the reason for miRNA detachment concerning expression versus repression because theoretically, miRNA levels can completely regulate miRNA mediated repression when its binding affinity is maximized. The threshold of this “perfect”miRNAs, which have a λ equal to 0, is completely defined by its expression, because the point until the threshold is working is equal to *θ* (compare online tool at λ value of 0).

Outside of classic miRNAs mechanisms, the increased number of studies showing small RNAs association with Ago2 or miRNA like size^10^,^17^,^58^,^59^,^60^ lack of detailed functional read outs: they showed either luciferase whole population^10^,^17^,^58^ or artificial overexpression^61^,^62^,^63^, that many times differs from the endogenous biogenesis pathways, meaning that the real physiological role is still undefined and contradictory in literature^62^,^63^,^64^,^65^,^66^,^67^,^68^. CLEAR-CLIP and CLASH studies showed how miRNAs have functional non-canonical binding including intronic and CDS mRNA regions^22^,^35^, indicating that predictions based on miRNA profiling and target analysis need a detailed functional output, therefore our system is helpful for these purposes.

Finally, functional studies between different conditions and further mechanistic definitions would be necessary to understand the three levels of miRNA-functionality. For example, a highly expressed miRNA included in Ago2 but bearing a characterized as low functional, might have high target-to-miRNA ratio, a different cellular location that inhibits its activity or even non-canonical binding. Importantly, these observations can only be reached through functional assay with high resolution as we introduced here, while simple profiling can lead to misinterpretation.

## Additional Information

**How to cite this article:** Lemus-Diaz, N. *et al*. Dissecting miRNA gene repression on single cell level with an advanced fluorescent reporter system. *Sci. Rep.* **7**, 45197; doi: 10.1038/srep45197 (2017).

**Publisher's note:** Springer Nature remains neutral with regard to jurisdictional claims in published maps and institutional affiliations.

## Supplementary Material

Supplementary Material
