# Supplementary material for: Dissecting miRNA gene repression on single cell level with an advanced fluorescent reporter system
Source: Sci Rep. 2017 Mar 24;7:45197. doi: 10.1038/srep45197 (PMC5364550; doi:10.1038/srep45197)
Supplement: Supplementary Material [file srep45197-s1.pdf]

**Supplementary Information**

# Dissecting miRNA gene repression on single cell level with an advanced fluorescent reporter system

Nicolas Lemus-Diaz, Kai O. Böker, Ignacio Rodriguez-Polo, Michael Mitter, Jasmin Preis, Maximilian Arlt, Jens Gruber

Junior Research Group Medical RNA Biology, German Primate Center, Kellnerweg 4, 37077 Göttingen, Germany

Figure. S1

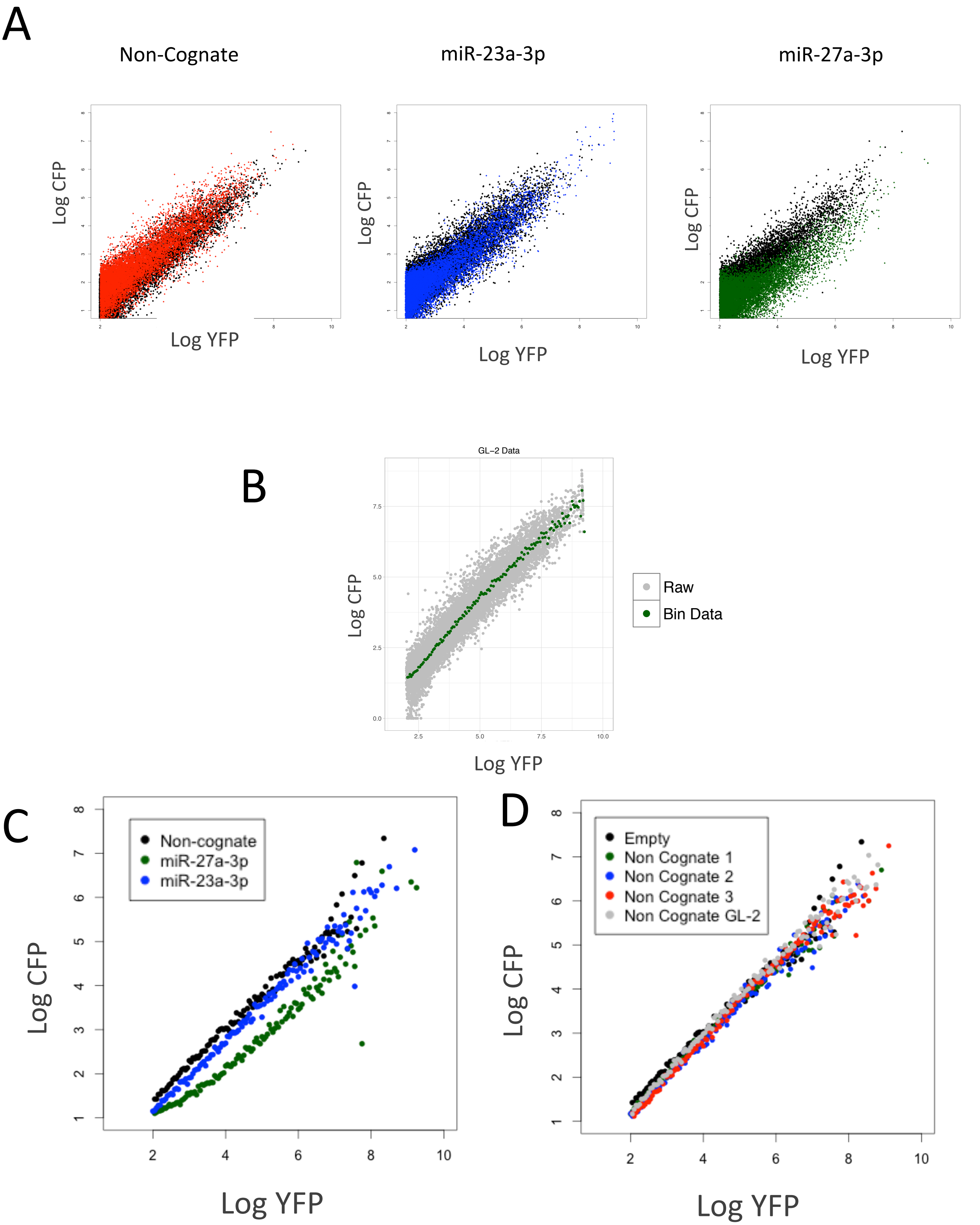
A
Non-Cognate
miR-23a-3p
miR-27a-3p
Log CFP
Log YFP
B
GL–2 Data
Raw
Bin Data
C
Non-cognate
miR-27a-3p
miR-23a-3p
D
Empty
Non Cognate 1
Non Cognate 2
Non Cognate 3
Non Cognate GL-2

**Figure S1**: **Dual Fluorescence Reporter System functionally characterized miRNA activity.** Hek293 were transfected with 3 different sensor constructs (miR-23a-3p, miR-27a-3p and Non-cognate) and evaluated after 72 h. **(A)** FACS scatter plots of YFP positive cells from constructs containing the miR-23-a-3p (Blue), miR-27a-3p (green) and non-cognate (Red) targeted site and the empty vector is depicted in black. Single cell analysis transfer function generation: YFP intensities were binned (1 to 8 at 0.05 intervals) and the average of each bin was calculated, **(B)** scatter plot of the GL-2 targeted construct (grey) and the calculated transfer function (green) **(C)** Transfer Function for miR-27a-3p and miR-23a-3p and **(D)** Transfer function for Three different cognate controls.

Figure. S2

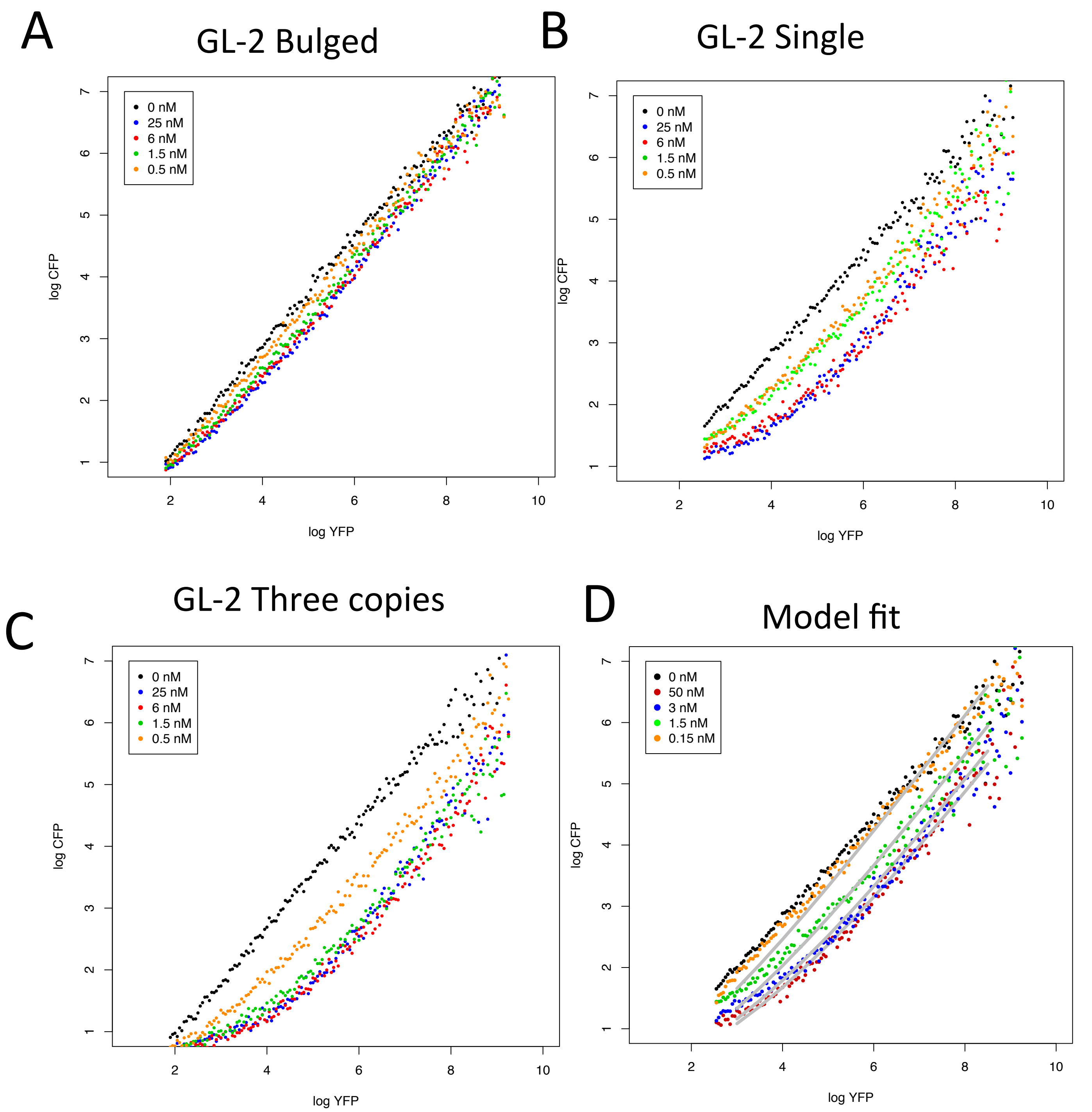
A
GL-2 Bulged
0 nM
25 nM
6 nM
1.5 nM
0.5 nM
log CFP
log YFP
B
GL-2 Single
0 nM
25 nM
6 nM
1.5 nM
0.5 nM
log CFP
log YFP
C
GL-2 Three copies
0 nM
25 nM
6 nM
1.5 nM
0.5 nM
log CFP
log YFP
D
Model fit
0 nM
50 nM
3 nM
1.5 nM
0.15 nM
log CFP
log YFP

**Figure S2: A molecular titration model for miRNA-mediated regulation describes different threshold behavior for siRNA activity.** Simulation was experimentally tested using GL-2 binding site included in the dual fluorescence reporter assay. To test λ three different target sites were evaluated: (A) Bulged 19 exact base pair bulged with 3 unpaired bases (B) one 21 perfectly complementary bases and (C) Three 21 exact complementary binding sites separated by 4 unpaired bases. (D) Fit lines derived from the model using nonlinear (weighted) least squares.

Figure. S3

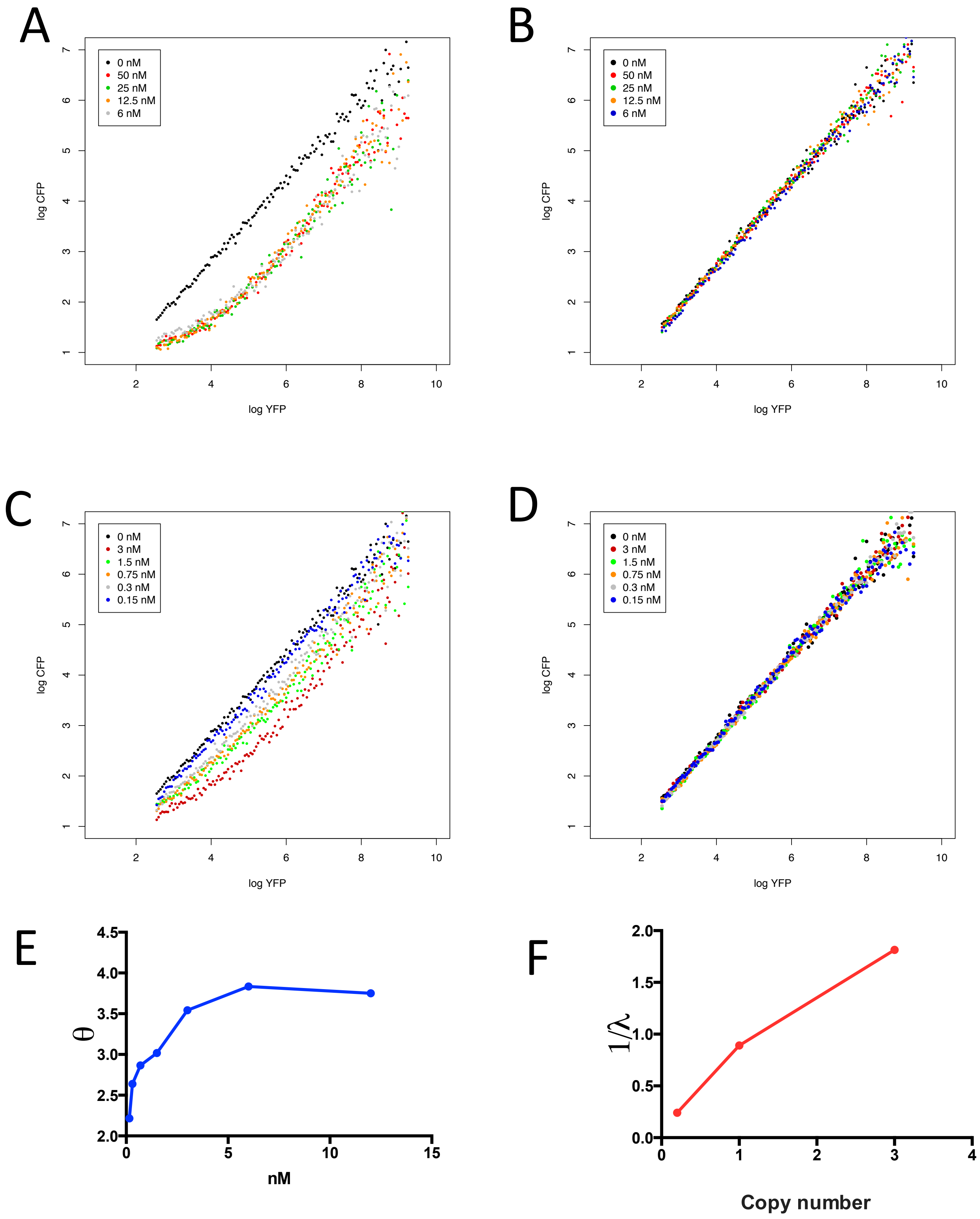
A
B
C
D
E
F
0 nM
50 nM
25 nM
12.5 nM
6 nM
3 nM
1.5 nM
0.75 nM
0.3 nM
0.15 nM
log CFP
log YFP
θ
nM
1/λ
Copy number

**Figure S3: Experimental range definition of θ and λ.** Dual Fluorescence Reporters for GL-2 and Non Cognate inserted sites were co-transfected in HEK293 with different concentration of GL-2 siRNA, after 72 h transfer functions were calculated: **(A)** GL-2 and **(B)** Non cognate targeted constructs co-transfected with 50 to 6 nM GL-2 siRNA. **(C)** GL-2 targeted and **(D)** Non-cognate targeted concentration construct co-transfected with 3 to 0.15 nM GL-2 siRNA.

Non-linear regression was performed to estimate θ and λ for all the Gl-2 siRNA experiments: first constant λ (binding capability) was selected arbitrary to estimate θ from 50 to 12 nM siRNA, then it was averaged and plotted: **(E)** Gl-2 siRNA Concentration vs. averaged estimated θ.

Estimated θ from 25 nM -GL-2 co-transfected was used to estimate λ for bulged, single and three copies constructs. **(E)** Copy number vs estimated 1/λ, bulged was arbitrary set to 0.5.

# Figure. S4

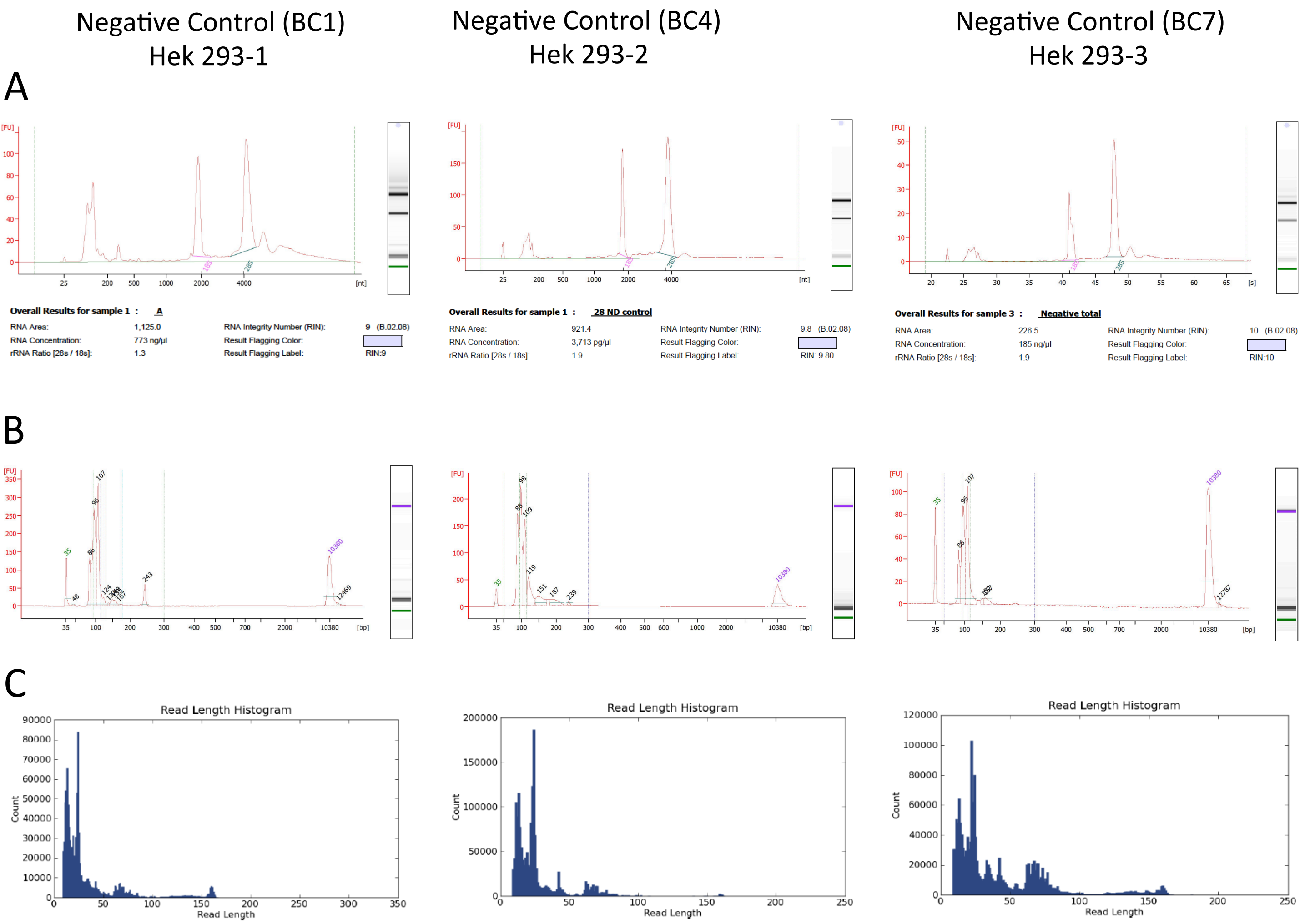

Negative Control (BC1)
Hek 293-1
Negative Control (BC4)
Hek 293-2
Negative Control (BC7)
Hek 293-3
A
Overall Results for sample 1 : A
RNA Area: 1,125.0
RNA Concentration: 773 ng/µl
rRNA Ratio [28s / 18s]: 1.3
RNA Integrity Number (RIN): 9 (B.02.08)
Result Flagging Color:
Result Flagging Label: RIN:9
Overall Results for sample 1 : 28 ND control
RNA Area: 921.4
RNA Concentration: 3,713 pg/µl
rRNA Ratio [28s / 18s]: 1.9
RNA Integrity Number (RIN): 9.8 (B.02.08)
Result Flagging Color:
Result Flagging Label: RIN: 9.80
Overall Results for sample 3 : Negative total
RNA Area: 226.5
RNA Concentration: 185 ng/µl
rRNA Ratio [28s / 18s]: 1.9
RNA Integrity Number (RIN): 10 (B.02.08)
Result Flagging Color:
Result Flagging Label: RIN:10
B
C
Read Length Histogram
Count
Read Length

**Figure S4: RNA quality control and library size distribution.** Three independent total RNA isolations were performed and RNA integrity tested by BioAnalyzer System **(A).** If RNA integrity number (RIN) was higher than 9 **(B)** Ion Torrent small RNA library preparation were performed and quantified and **(C).** Diluted library (18 pM) was sequenced via IonTorrent System.

Figure. S5

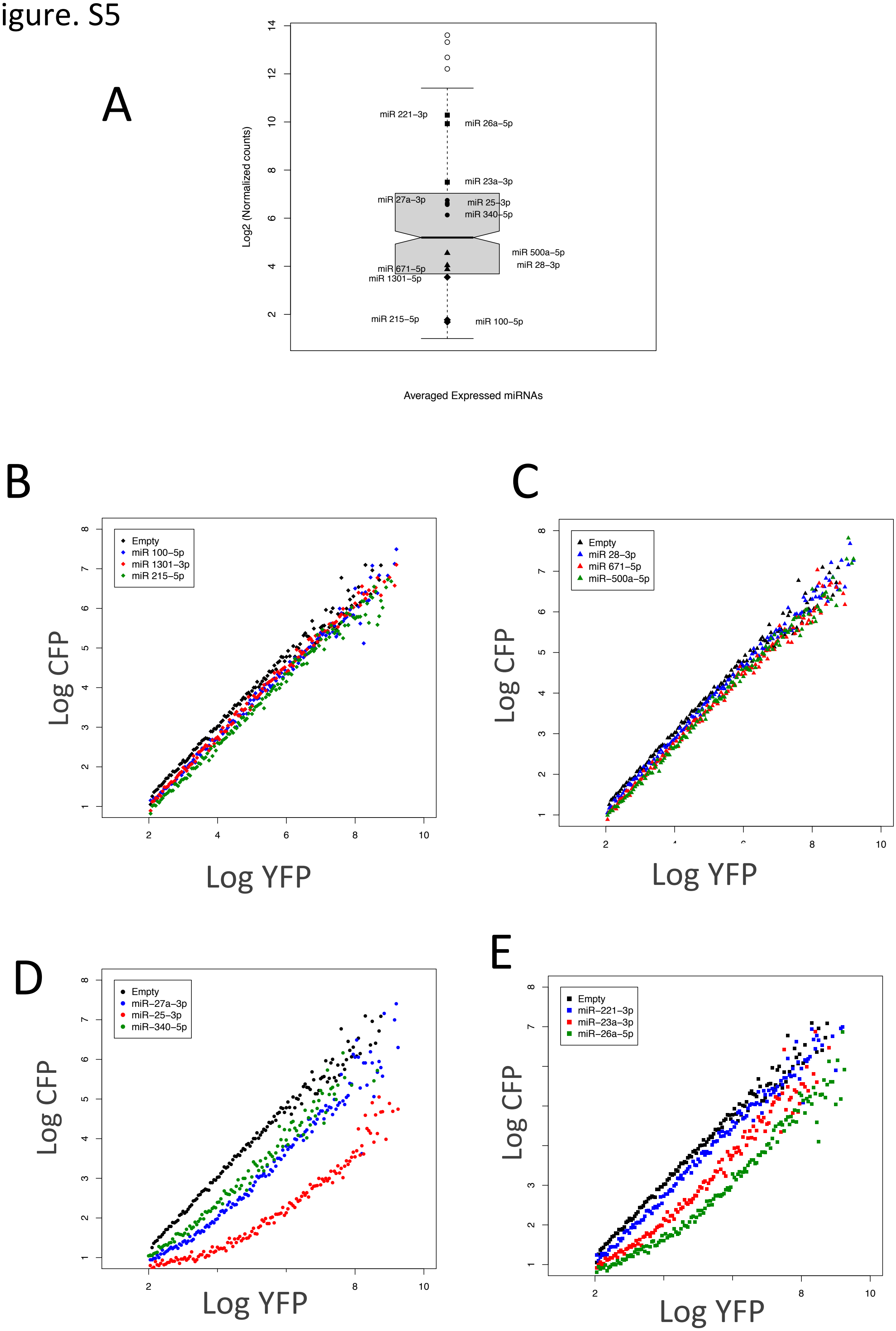
A
Log2 (Normalized counts)
Averaged Expressed miRNAs
miR 221–3p
miR 26a–5p
miR 23a–3p
miR 27a–3p
miR 25–3p
miR 340–5p
miR 500a–5p
miR 28–3p
miR 671–5p
miR 1301–5p
miR 215–5p
miR 100–5p
B
Empty
miR 100–5p
miR 1301–3p
miR 215–5p
Log CFP
Log YFP
C
Empty
miR 28–3p
miR 671–5p
miR–500a–5p
Log CFP
Log YFP
D
Empty
miR–27a–3p
miR–25–3p
miR–340–5p
Log CFP
Log YFP
E
Empty
miR–221–3p
miR–23a–3p
miR–26a–5p
Log CFP
Log YFP

**Figure S5: Dual Fluorescence Reporter System UTA is suitable for single cell measurements of endogenous miRNAs.** Three miRNAs libraries from 3 independent Hek293 cultures were sequenced by IonTorrent platform and **(A)** twelve different miRNAs were selected based on their expression levels (Mean Normalized Counts) and quartile position, then 12 different reporters for were generated and functionally tested using FACS. UTA Transfer function for candidates located: **(B)** below the first quartile, **(C)** between the median and the first quartile, **(C)** above median and the third quartile; **(D)** above the third quartile.

# Figure. S6

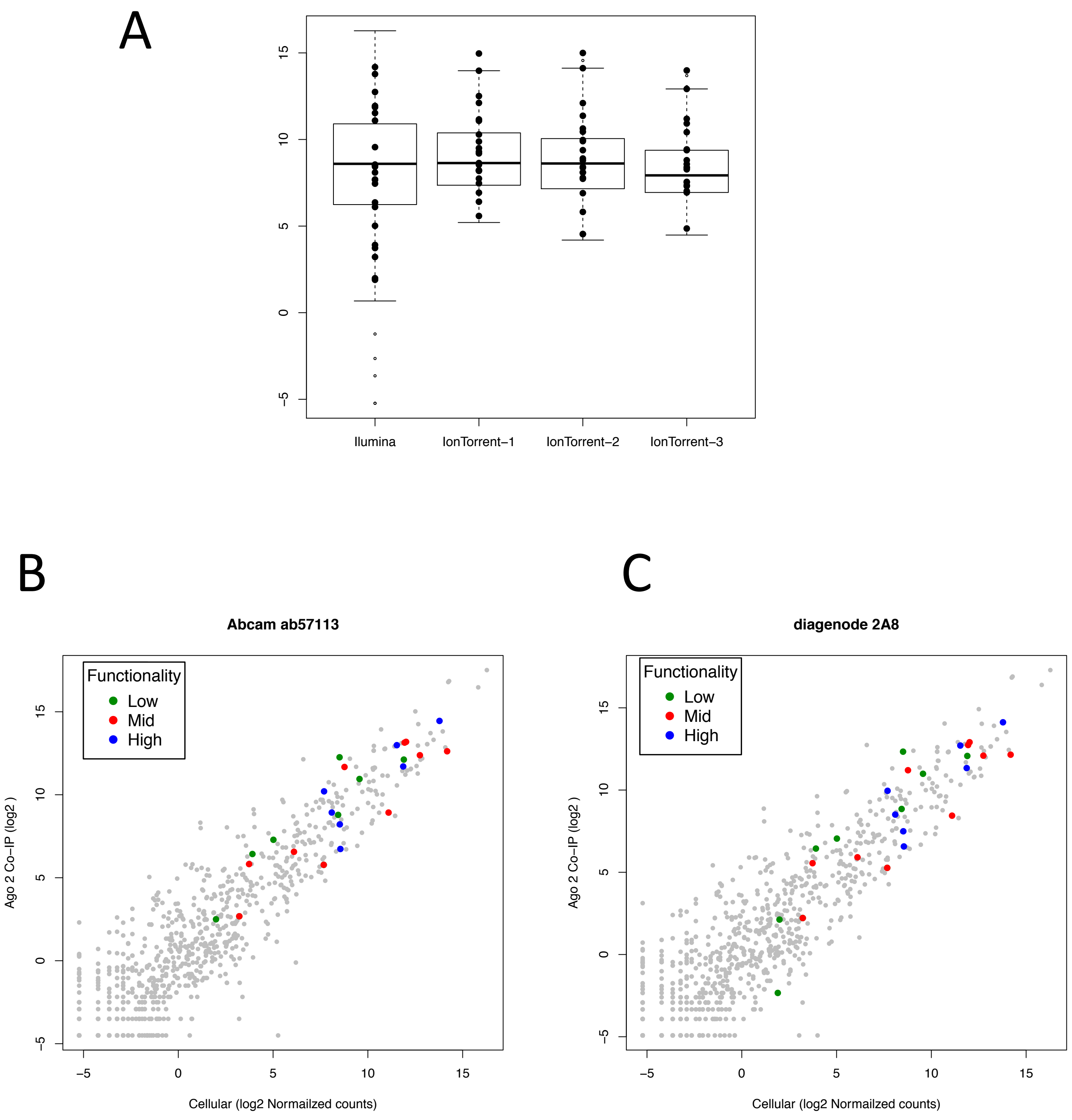

A
15
10
5
0
−5
Ilumina
IonTorrent−1
IonTorrent−2
IonTorrent−3
B
Abcam ab57113
Functionality
Low
Mid
High
Ago 2 Co−IP (log2 )
Cellular (log2 Normailzed counts)
C
diagenode 2A8
Functionality
Low
Mid
High
Ago 2 Co−IP (log2 )
Cellular (log2 Normailzed counts)

**Figure S6: AGO2 miRNA association does not explain functional groups from dual fluorescence reporters.** Read count tables from HEK293 total cellular content and AGO2 IP RNA were retrieved from (http://www.ncbi.nlm.nih.gov/geo/query/acc.cgi?acc=GSE58127). Tested miRNAs are similar distributed between NGS platforms: Illumina platform: **(A)** miRNA Data from HEK293 cells (GSE58127) Libraries were normalized by RPKM together with the Ion Torrent produced libraries in this study; miRNA distribution was plotted using Box plot and tested miRNAs are depicted as bold points.

AGO2 Co-IP data from two independent antibodies were compared with total cellular miRNA content Read counts plotted total cellular content vs. (B) Antibody Abcam57113 and (C) Diogenode 2A8; functional groups are depicted on green (low functional), red (Mid functional), blue (High Functional).

Figure. S7

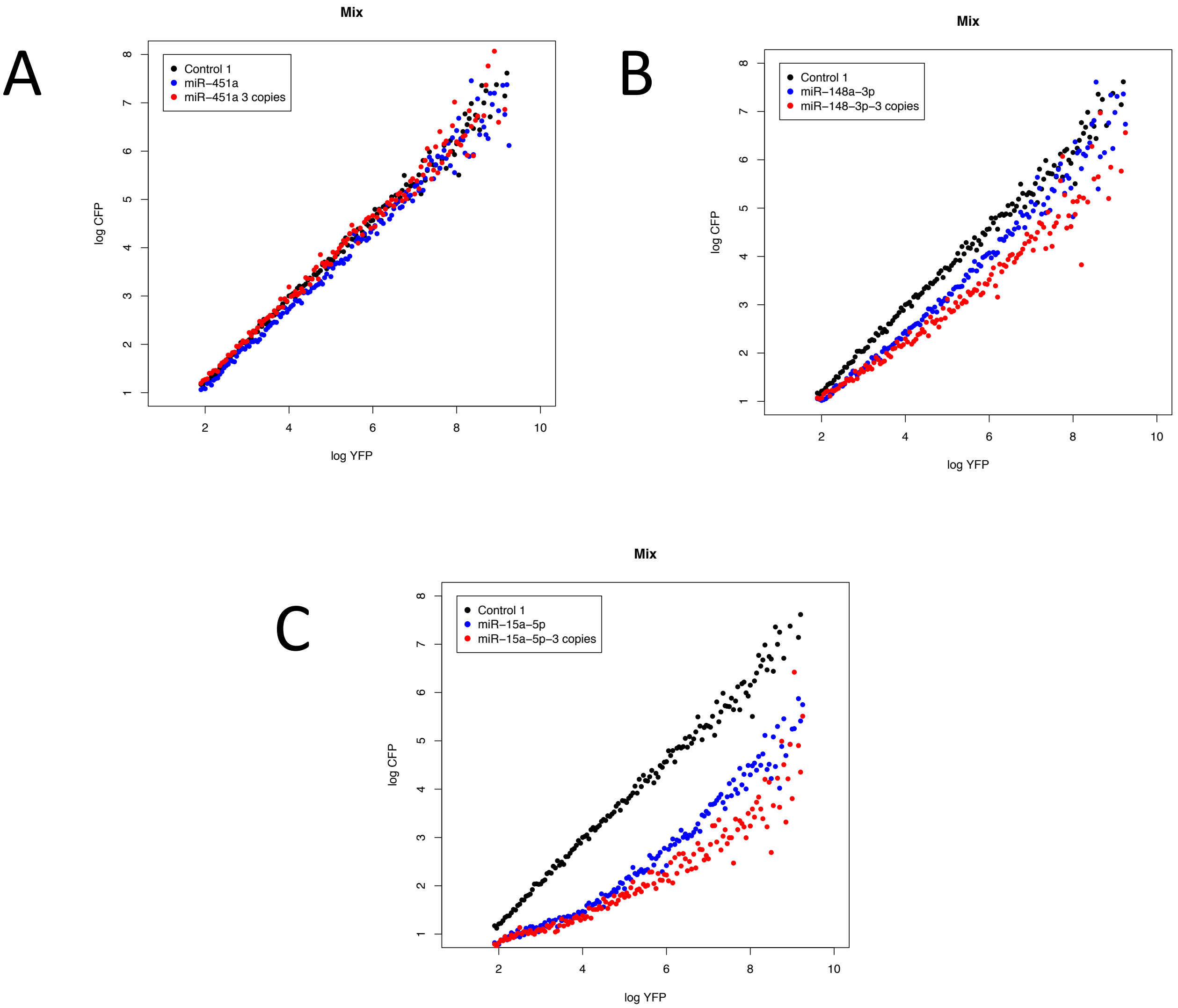
A
Mix
Control 1
miR−451a
miR−451a 3 copies
log CFP
log YFP
B
Mix
Control 1
miR−148a−3p
miR−148−3p−3 copies
log CFP
log YFP
C
Mix
Control 1
miR−15a−5p
miR−15a−5p−3 copies
log CFP
log YFP

**Figure S7 Threshold modulation of endogenous miRNAs:** Three miRNAs were selected (one for each functional group) and one and three copies of perfect complementarity were inserted in UTA reporter. HEK293 cells were transfected and evaluated after 72 h. **(A)** Low functional miRNA, **(B)** mid Functional miRNA and **(C)** High Functional RNA.

Figure. S8

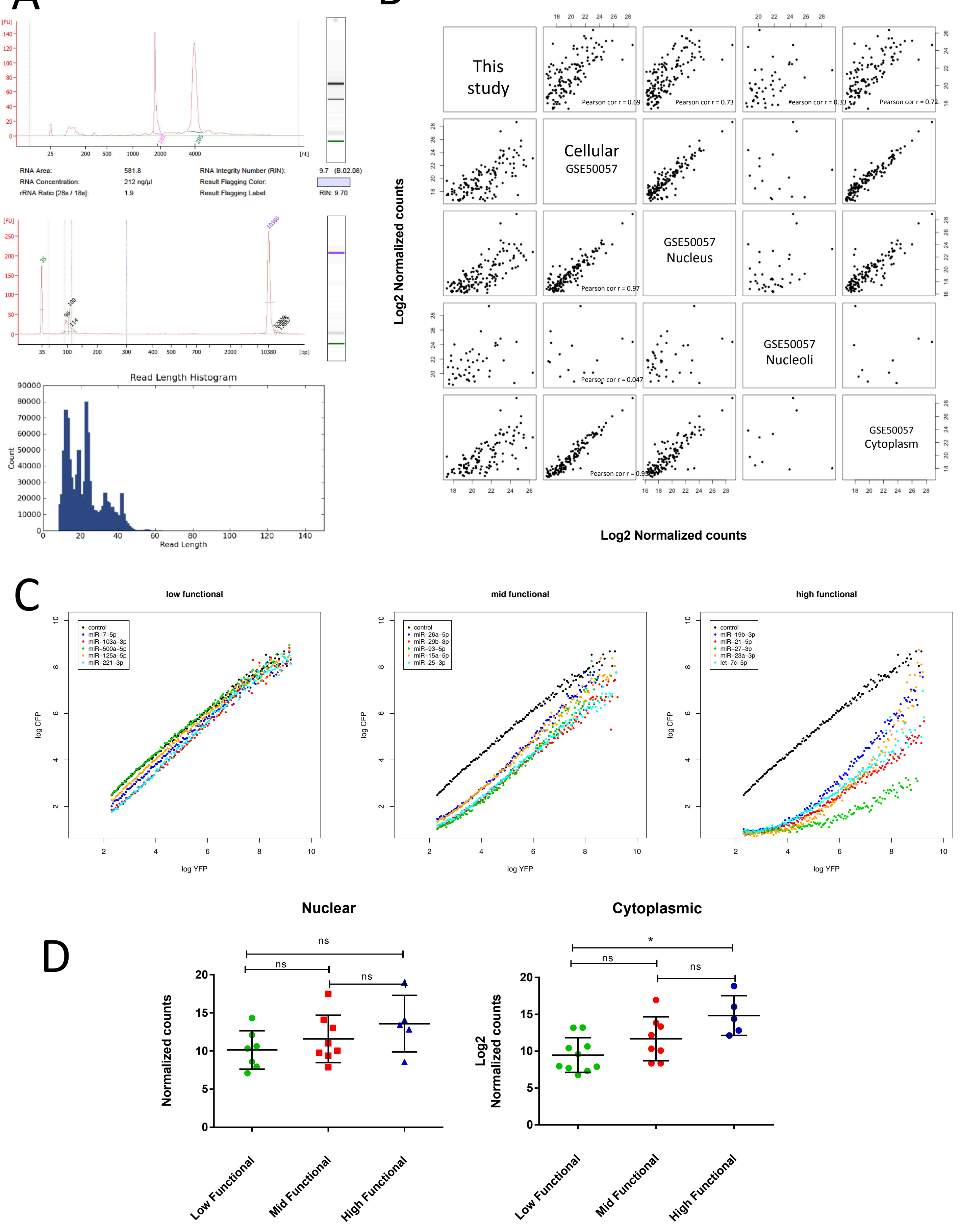

A
RNA Area: 581.8
RNA Concentration: 212 ng/µl
rRNA Ratio [28s / 18s]: 1.9
RNA Integrity Number (RIN): 9.7 (B.02.08)
Result Flagging Color:
Result Flagging Label: RIN: 9.70
Read Length Histogram
Count
Read Length
B
This study
Cellular GSE50057
GSE50057 Nucleus
GSE50057 Nucleoli
GSE50057 Cytoplasm
Pearson cor r = 0.69
Pearson cor r = 0.73
Pearson cor r = 0.33
Pearson cor r = 0.72
Pearson cor r = 0.97
Pearson cor r = 0.047
Pearson cor r = 0.95
Log2 Normalized counts
Log2 Normalized counts
C
low functional
control
miR−7−5p
miR−103a−3p
miR−500a−5p
miR−125a−5p
miR−221−3p
mid functional
control
miR−26a−5p
miR−29b−3p
miR−93−5p
miR−15a−5p
miR−25−3p
high functional
control
miR−19b−3p
miR−21−5p
miR−27−3p
miR−23a−3p
let−7c−5p
log CFP
log YFP
D
Nuclear
Normalized counts
ns
ns
ns
Low Functional
Mid Functional
High Functional
Cytoplasmic
Log2 Normalized counts
*
ns
ns
Low Functional
Mid Functional
High Functional

**Figure S8. Functional characterization of miRNAs from single cell reporter assays in Hela cells. (A)** Bionalyzer graphs for total RNA quality, prepared library and read distribution of sequenced library from Hela cells. GSE50057 raw data and produced library were aligned to miRNA and snoRNA database, **(B)** Scatter plot from normalized reads with Pearson correlation between the used libraries.

All tested HEK293 miRNAs reporters were transfected in Hela cells and UTA transfer function generated: **(C)** miRNA functional outputs were classified as before low functional (left), mid functional (center) and high functional (right). **(D)** Read count distribution for the three ordinal qualitative groups for Nuclear (left) and Cytoplasmic (right) libraries.

**Supplementary Table 1**: DNA oligonucleotides used in this study.

| miRNA | Oligos (5'-3' XhoI or NotI overhangs) |
|---|---|
| hsa-miR-100-5p | ggccagcttctttacagtgctgccttg |
| | tcgacaaggcagcactgtaaagaagct |
| hsa-miR-1301-3p | ggcctgcagctgcctgggagtgacttc |
| | tcgagaagtcactcccaggcagctgca |
| hsa-miR-215-5p | ggccatgacctatgaattgacagac |
| | tcgagtctgtcaattcataggtcat |
| hsa-miR-28-3p | ggcccactagattgtgagctcctgga |
| | tcgatccaggagctcacaatctagtg |
| hsa-miR-29a-3p | ggcctagcaccatctgaaatcggtta |
| | tcgataaccgatttcagatggtgcta |
| hsa-miR-24-3p | ggcctggctcagttcagcaggaacag |
| | tcgactgttcctgctgaactgagcca |
| hsa-miR-451a | ggccaaaccgttaccattactgagtt |
| | tcgaaactcagtaatggtaacggttt |
| hsa-miR 7-5p | ggcctggaagactagtgattttgttgt |
| | tcgaacaacaaaatcactagtcttcca |
| hsa-miR-15a-5p | ggcctagcagcacataatggtttgtg |
| | tcgacacaaaccattatgtgctgcta |
| hsa-miR-19b-3p | ggcctgtgcaaatccatgcaaaactga |
| | tcgatcagttttgcatggatttgcaca |
| hsa-miR-26a-5p | ggccttcaagtaatccaggataggct |
| | tcgaagcctatcctggattacttgaa |
| hsa-miR-29b-3p | ggcctagcaccatttgaaatcagtgtt |
| | tcgaaacactgatttcaaatggtgcta |
| hsa-miR-93-5p | ggcccaaagtgctgttcgtgcaggtag |
| | tcgactacctgcacgaacagcactttg |
| hsa-miR-103a-3p | ggccagcagcattgtacagggctatga |
| | tcgatcatagccctgtacaatgctgct |
| hsa-miR-148a-3p | ggcctcagtgcactacagaactttgt |
| | tcgaacaaagttctgtagtgcactga |
| hsa-miR-181a-5p | ggccaacattcaacgctgtcggtgagt |
| | tcgaactcaccgacagcgttgaatgtt |
| hsa-miR-221-3p | ggccagctacattgtctgctgggtttc |
| | tcgagaaacccagcagacaatgtagct |
| hsa-let-7c-5p | ggcctgaggtagtaggttgtatggtt |
| | tcgaaaccatacaacctactacctca |
| hsa-miR-18a-5p | ggcctaaggtgcatctagtgcagatag |
| | tcgactatctgcactagatgcacctta |
| hsa-miR-19b-3p | ggcctgtgcaaatccatgcaaaactga |
| | tcgatcagttttgcatggatttgcaca |
| hsa-miR-25-3p | ggcccattgcacttgtctcggtctga |
| | tcgatcagaccgagacaagtgcaatg |

| | |
|---|---|
| hsa-miR-125a-5p | ggcctccctgagaccctttaacctgtga |
| | tcgatcacaggttaaagggtctcaggga |
| hsa-miR-500a-5p | ggcctaatccttgctacctgggtgaga |
| | tcgatctcacccaggtagcaaggatta |
| hsa-miR-340-5p | ggccttataaagcaatgagactgatt |
| | tcgaaatcagtctcattgctttataa |
| hsa-miR-671-5p | ggccaggaagccctggaggggctggag |
| | tcgactccagcccctccagggcttcct |
| hsa-miR-3607-5p | ggccgcatgtgatgaagcaaatcagt |
| | tcgaactgatttgcttcatcacatgc |
| hsa-miR-3607-3p | ggccactgtaaacgctttctgatg |
| | tcgacatcagaaagcgtttacagt |
| hsa-miR-21-5p | ggcctagcttatcagactgatgttga |
| | tcgatcaacatcagtctgataagcta |
| hsa-miR-27a 3-p | ggccttcacagtggctaagttccgc |
| | tcgagcggaacttagccactgtgaa |
| hsa-miR-23a 3-p | ggccatcacattgccagggatttcc |
| | tcgaggaaatccctggcaatgtgat |
| hsa-miR-27a 3-p bulged | ggccttcacaggtctaagttccgc |
| | tcgagcggaacttagacctgtgaa |
| GL-2 | ggcccacgtacgcggaatacttcgaaa |
| | tcgatttcgaagtattccgcgtacgtg |
| GL-2 bulged | ggcccacgtacgcggtctacttcgaaa |
| | tcgatttcgaagtagaccgcgtacgtg |

**Supplementary Table 2:** Summarized data of miRNA expression, functional classifications and results of transfer function regression

| miRNAs | Functional Group* | Lambda[1,3] | Theta[1] | Normalized Read Counts[2] |
|---|---|---|---|---|
| hsa-miR-7-5p | MF | 1,5 | 3,4 | 192,3 |
| hsa-miR-103a-3p | MF | 1,3 | 3,2 | 1949,3 |
| hsa-miR-148a-3p | MF | 2,1 | 3,1 | 193,6 |
| hsa-miR-181a-5p | MF | 3,0 | 3,6 | 188,4 |
| hsa-miR-221-3p | MF | 1,0 | 3,0 | 1249,9 |
| hsa-miR-340-5p | MF | 2,3 | 3,2 | 107,6 |
| hsa-miR-27a-3p | MF | 2,7 | 4,0 | 70,0 |
| hsa-miR-100-5p | LF | 4,0 | 3,1 | 3,2 |
| hsa-miR-1301-3p | LF | 3,7 | 2,9 | 11,7 |
| hsa-miR-215-5p | LF | 2,6 | 2,9 | 3,4 |
| hsa-miR-28-3p | LF | 3,9 | 2,6 | 16,4 |
| hsa-miR-29a-3p | LF | 2,2 | 2,7 | 94,2 |
| hsa-miR-24-3p | LF | 2,3 | 3,2 | 261,1 |
| hsa-miR-29b-3p | LF | 1,9 | 3,0 | 352,0 |
| hsa-miR-451a | LF | 8,0 | 3,9 | 0,0 |
| hsa-miR-125a-5p | LF | 2,3 | 3,2 | 239,8 |
| hsa-miR-500a-5p | LF | 4,8 | 3,4 | 23,4 |
| hsa-miR-671-5p | LF | 5,7 | 4,0 | 14,7 |
| hsa-miR-3607-5p | LF | 4,0 | 3,5 | 456,4 |
| hsa-miR-3607-3p | LF | 3,6 | 3,1 | 439,8 |
| hsa-miR-23a-3p | LF | 1,6 | 2,3 | 180,7 |
| hsa-miR-15a-5p | HF | 1,7 | 5,1 | 424,3 |
| hsa-miR-19b-3p | HF | 1,7 | 7,0 | 12501,7 |
| hsa-miR-26a-5p | HF | 1,2 | 3,9 | 969,5 |
| hsa-miR-93-5p | HF | 1,6 | 6,0 | 1365,8 |
| hsa-let-7c-5p | HF | 1,3 | 5,5 | 131,9 |
| hsa-miR-18a-5p | HF | 1,1 | 6,0 | 6586,8 |
| hsa-miR-25-3p | HF | 1,4 | 6,0 | 98,2 |
| hsa-miR-21-5p | HF | 2,6 | 5,6 | 585,5 |

*Low Functional= LF, Mid Functional=MF, High Functional=HF.
1.Lambda and Theta values were calculated using non-linear least squares.
2.Counts were Normalized using the DEseq Package on R.
3.Lambda ($\lambda \sim K_{off}$ or $\lambda \sim 1/K_{on}$)

**Supplementary Table 3:** Summarized data of miRNA expression and functional classification for Hela Cells. Data retrieved from GEO GSE50057*

| miRNA | Cellular | Nu | Cyto | No | Ratio | Function |
|---|---|---|---|---|---|---|
| **hsa_let_7c_5p** | 1977,11 | 382,08 | 4471,07 | NA | 11,70 | HF |
| **hsa_miR_100_5p** | 7445,34 | 4400,72 | 9448,87 | NA | 2,15 | LF |
| **hsa_miR_103a_3p** | 19423,79 | 20570,80 | 9289,89 | NA | 0,45 | LF |
| **hsa_miR_125a_5p** | 1371,51 | 1275,87 | 765,05 | 29529,546 | 0,60 | LF |
| **hsa_miR_1301_3p** | 133,59 | NA | NA | NA | #VALUE! | LF |
| **hsa_miR_148a_3p** | 507,64 | 388,90 | 248,39 | NA | 0,64 | LF |
| **hsa_miR_15a_5p** | 400,77 | 238,80 | 327,88 | NA | 1,37 | MF |
| **hsa_miR_181a_5p** | 2333,35 | 1582,89 | 1361,19 | 1139,508 | 0,86 | LF |
| **hsa_miR_18a_5p** | 21009,04 | 17377,72 | 14883,70 | NA | 0,86 | MF |
| **hsa_miR_19b_3p** | 8905,91 | 7361,82 | 7163,65 | NA | 0,97 | HF |
| **hsa_miR_21_5p** | 417215,12 | 519530,32 | 466690,51 | 3678,984 | 0,90 | HF |
| **hsa_miR_221_3p** | 1059,80 | 245,62 | 1609,59 | NA | 6,55 | LF |
| **hsa_miR_23a_3p** | 58057,62 | 17241,26 | 67354,22 | NA | 3,91 | HF |
| **hsa_miR_24_3p** | 155844,50 | 183356,42 | 126402,18 | NA | 0,69 | MF |
| **hsa_miR_25_3p** | 1416,04 | 880,14 | 1082,99 | NA | 1,23 | MF |
| **hsa_miR_26a_5p** | 1994,92 | 668,64 | 4659,85 | 423,246 | 6,97 | MF |
| **hsa_miR_27a_3p** | 16039,54 | 11052,97 | 21679,73 | 1139,508 | 1,96 | HF |
| **hsa_miR_28_3p** | NA | NA | 158,97 | NA | #VALUE! | LF |
| **hsa_miR_29a_3p** | 605,60 | 1037,07 | 327,88 | NA | 0,32 | LF |
| **hsa_miR_29b_3p** | 5423,70 | 2067,31 | 1281,71 | NA | 0,62 | MF |
| **hsa_miR_500a_5p** | 106,87 | NA | 208,65 | NA | #VALUE! | LF |
| **hsa_miR_671_5p** | 418,58 | NA | 238,46 | 21650,659 | #VALUE! | LF |
| **hsa_miR_7_5p** | NA | 136,46 | 109,29 | NA | 0,80 | LF |
| **hsa_miR_93_5p** | 13323,24 | 8303,37 | 10333,14 | NA | 1,24 | MF |

***Bai, B., Yegnasubramanian, S., Wheelan, S. J. & Laiho, M. RNA-Seq of the nucleolus reveals abundant SNORD44-derived small RNAs. *PloS one* 9, e107519, doi:10.1371/journal.pone.0107519 (2014).**

**Supplementary Information**

Model Simulation online tool:
https://medicalrnabiology.shinyapps.io/pUTApp/

$$r = 1/2(r_0 - \lambda - \theta + \sqrt{(r - \lambda - \theta)^2 + 4\lambda r_0})$$

where:

$$r_0 = \frac{K_R}{y_R} \qquad \lambda = \frac{y_{R^*} + K_{off}}{K_{on}} \qquad \theta = \frac{y_{R^*}}{y_R}\left[miRNA\right]_T$$

Steady state solutions for r (log CFP) as a function of $r_0$ (log YFP)
Theta and Lambda can be introduced.

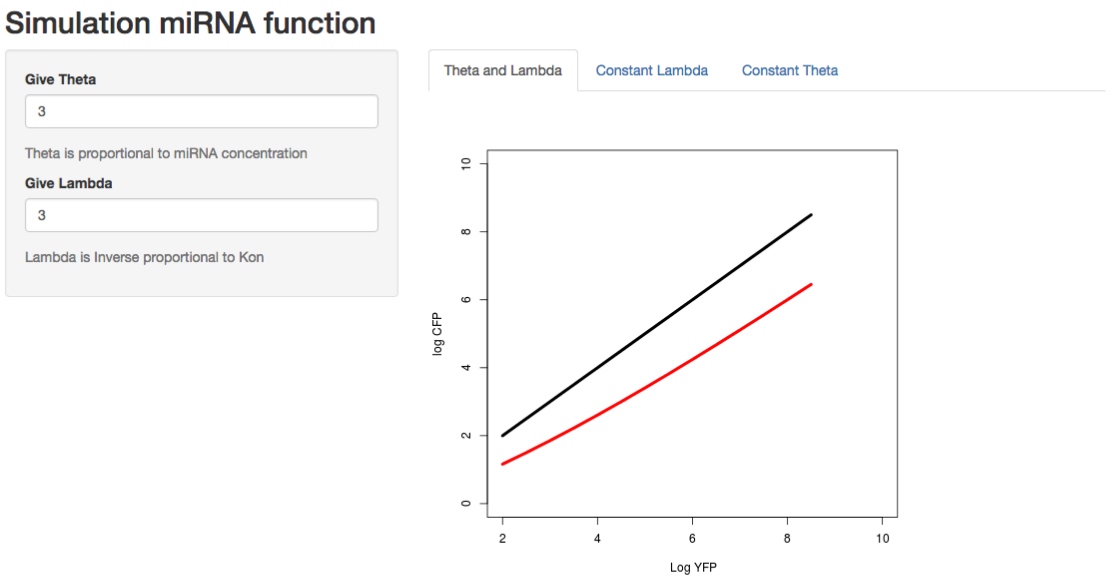


Several Theta values are computed (simulating miRNA concentration increments).

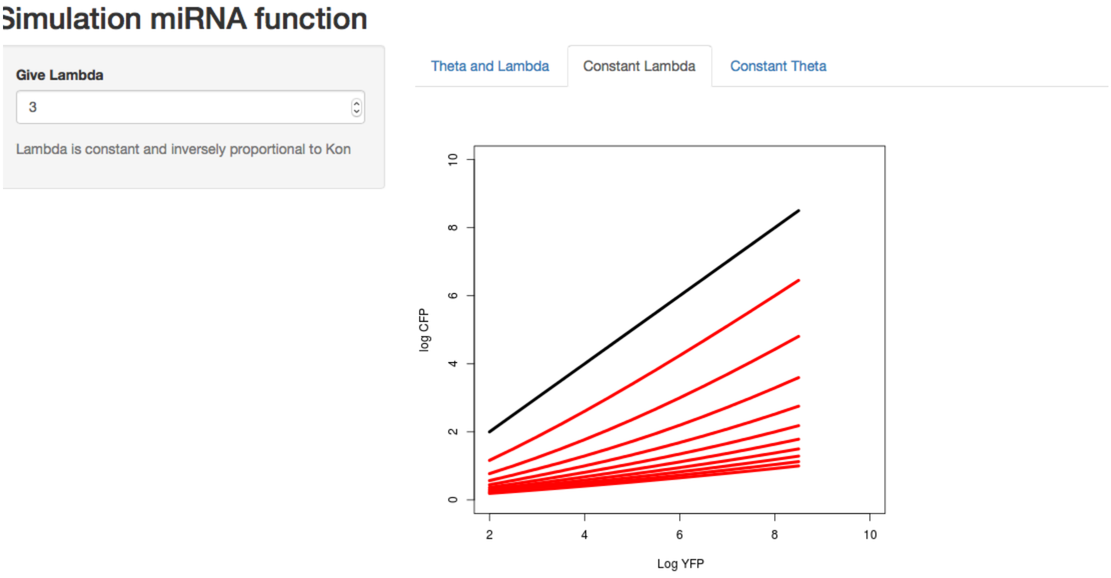


Several lambda values are computed (simulating changes on miRNA binding capability).

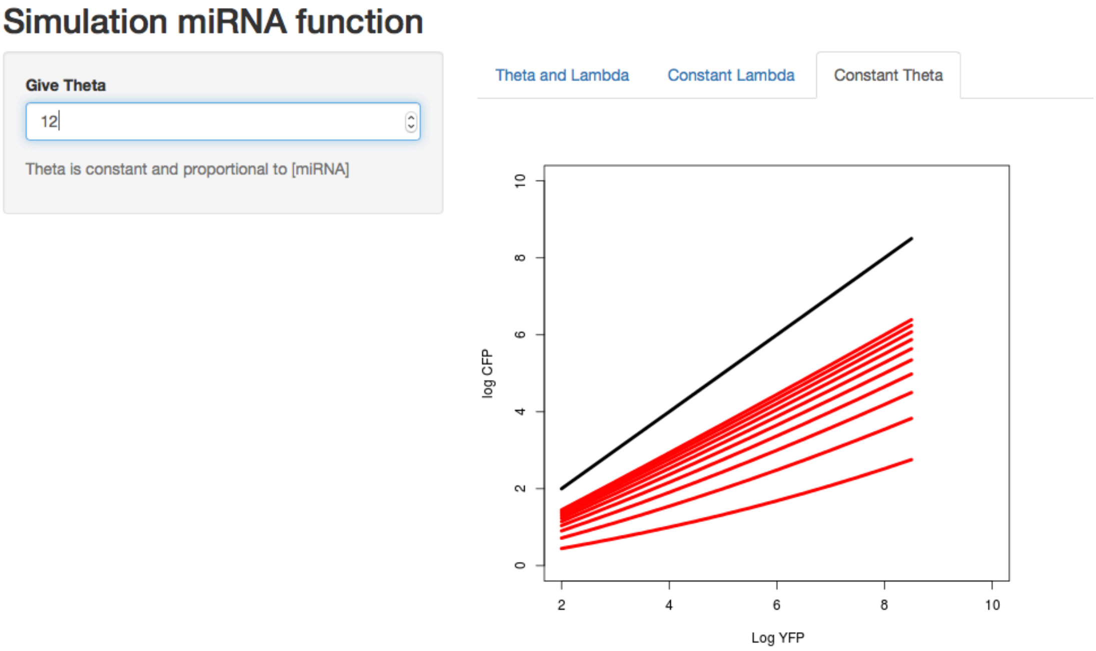

**Supplementary extended data**

All tested miRNA transfer function were calculated reduction bin size from 0.05 to 0.02. Non-linear regression was performed using least squares. Transfer function for miRNA are depicted in Green and Non-cognate control in black. Lambda ,Values and RSS are included.

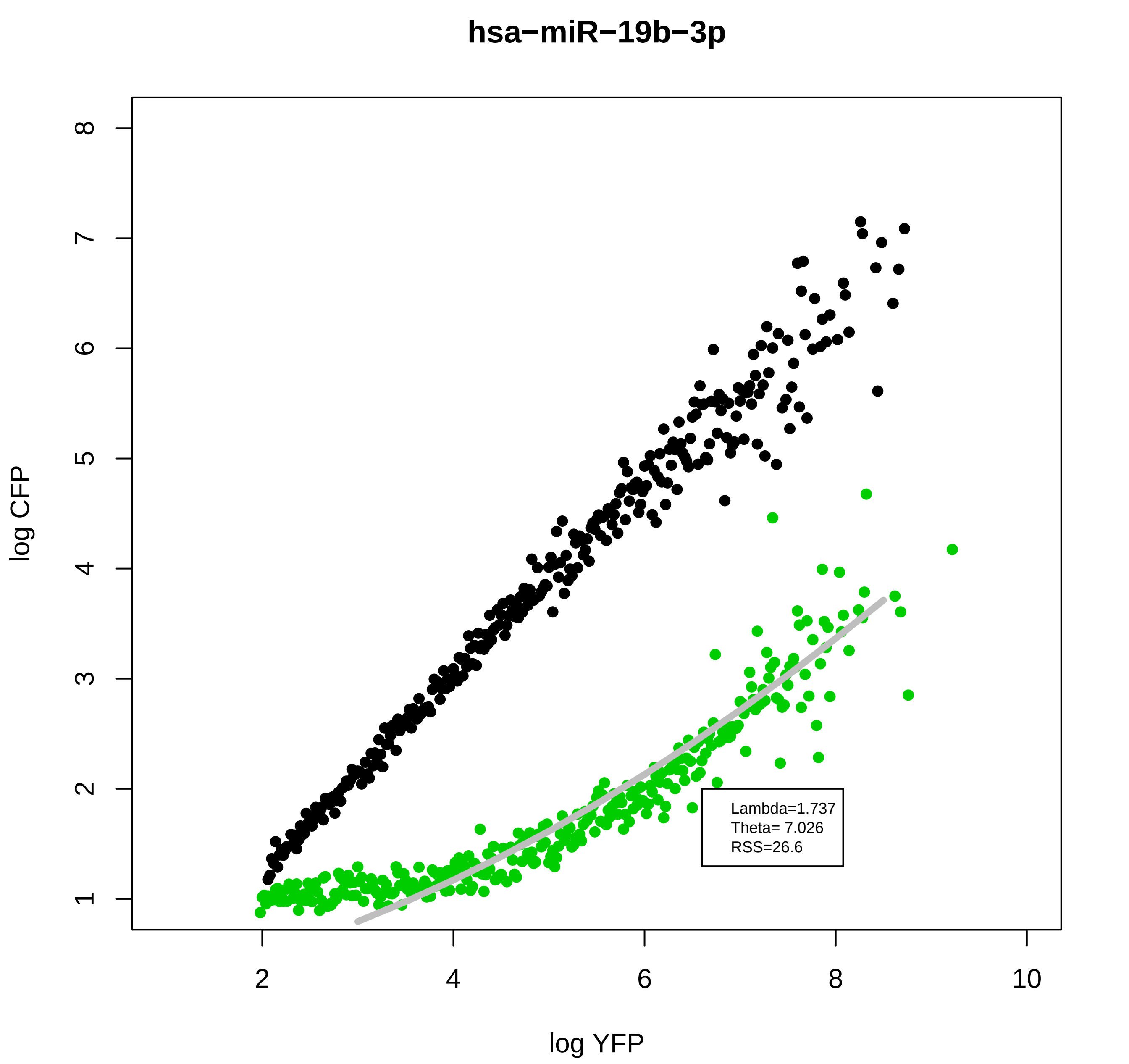
hsa−miR−19b−3p
log CFP
log YFP
1
2
3
4
5
6
7
8
2
4
6
8
10
Lambda=1.737
Theta= 7.026
RSS=26.6

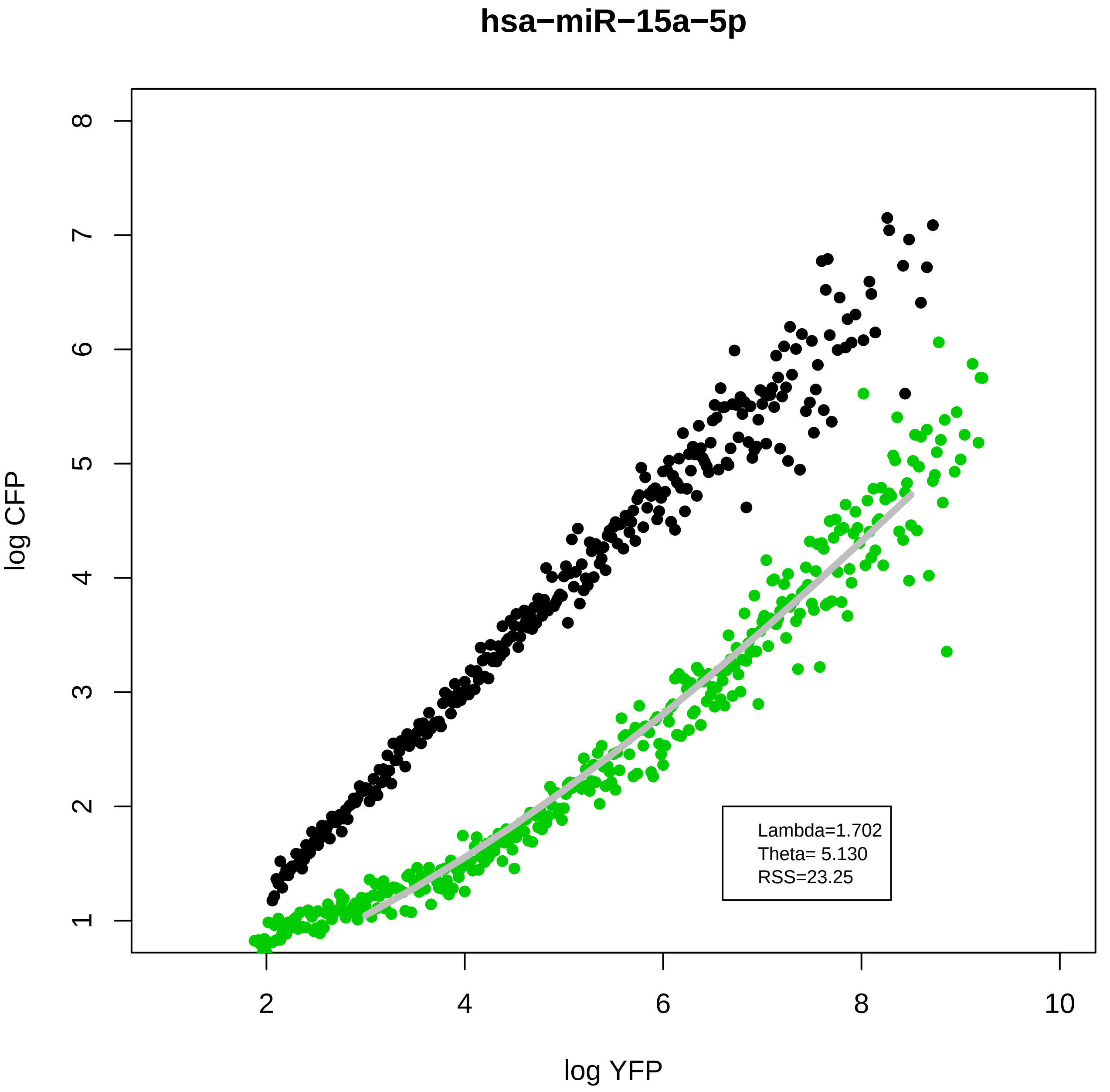

hsa−miR−15a−5p
log CFP
log YFP
1
2
3
4
5
6
7
8
2
4
6
8
10
Lambda=1.702
Theta= 5.130
RSS=23.25

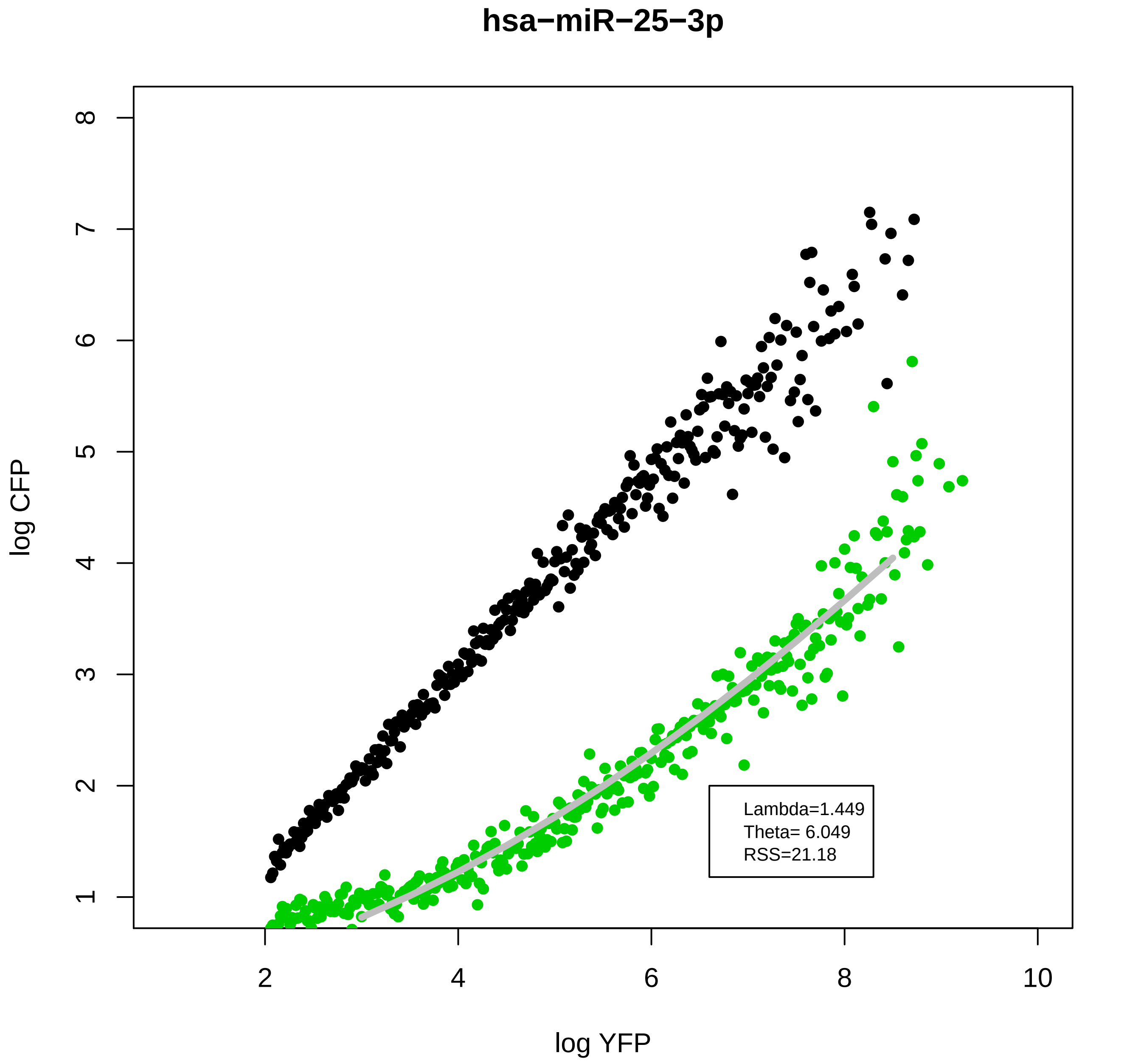

hsa−miR−25−3p
Lambda=1.449
Theta= 6.049
RSS=21.18
log CFP
log YFP
1
2
3
4
5
6
7
8
2
4
6
8
10

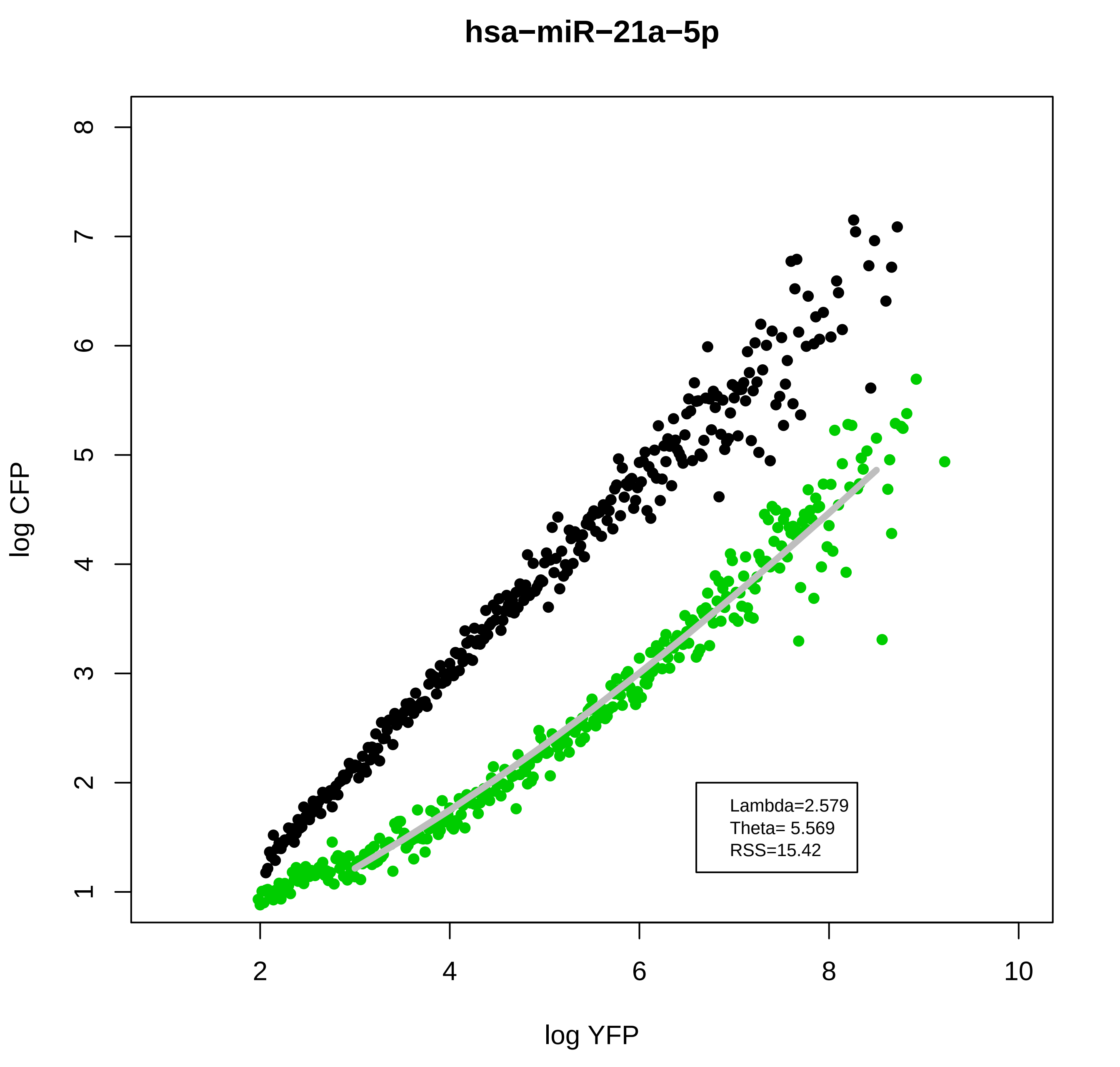
hsa−miR−21a−5p
log CFP
log YFP
1
2
3
4
5
6
7
8
2
4
6
8
10
Lambda=2.579
Theta= 5.569
RSS=15.42

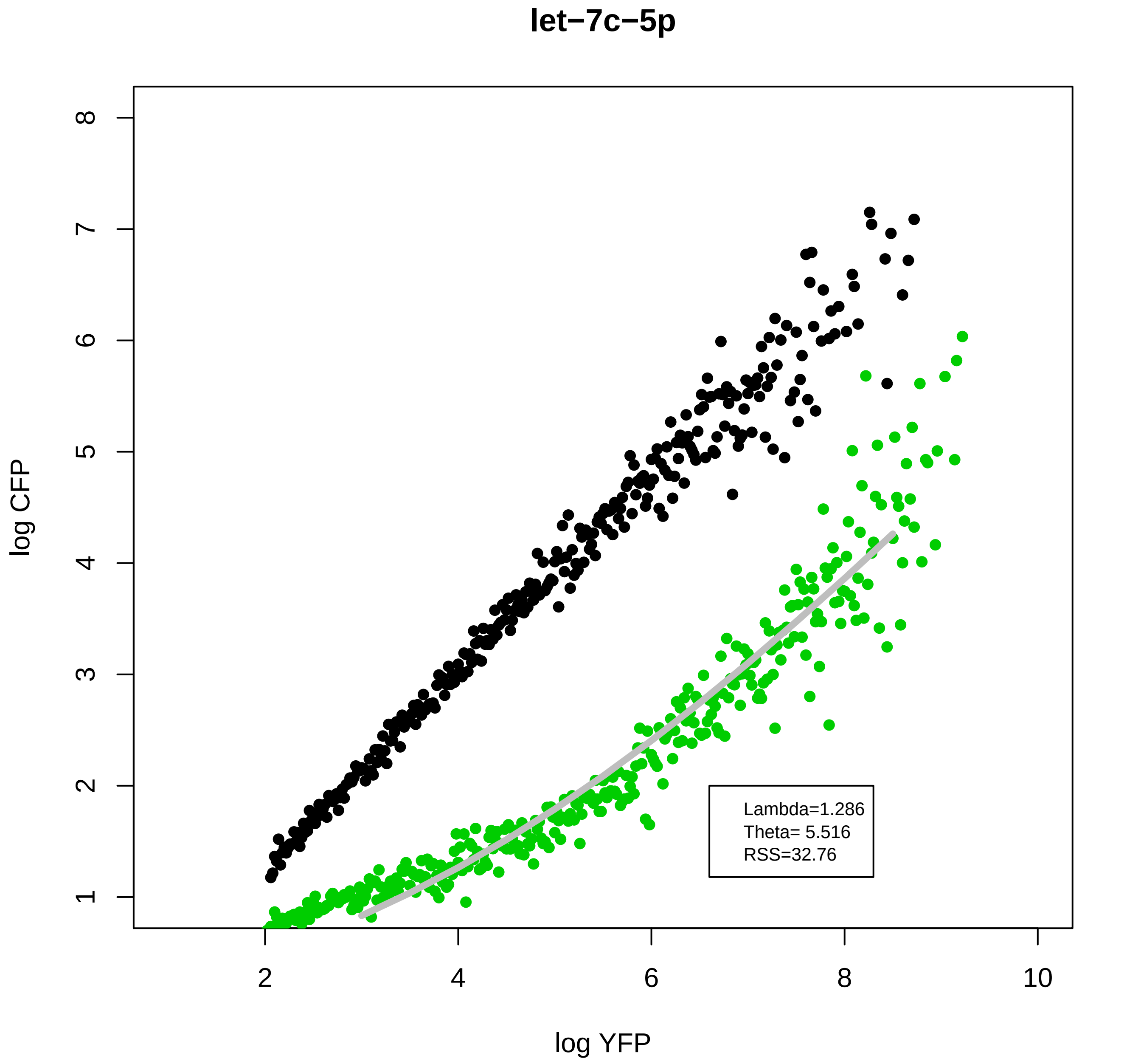
let−7c−5p
log CFP
log YFP
1
2
3
4
5
6
7
8
2
4
6
8
10
Lambda=1.286
Theta= 5.516
RSS=32.76

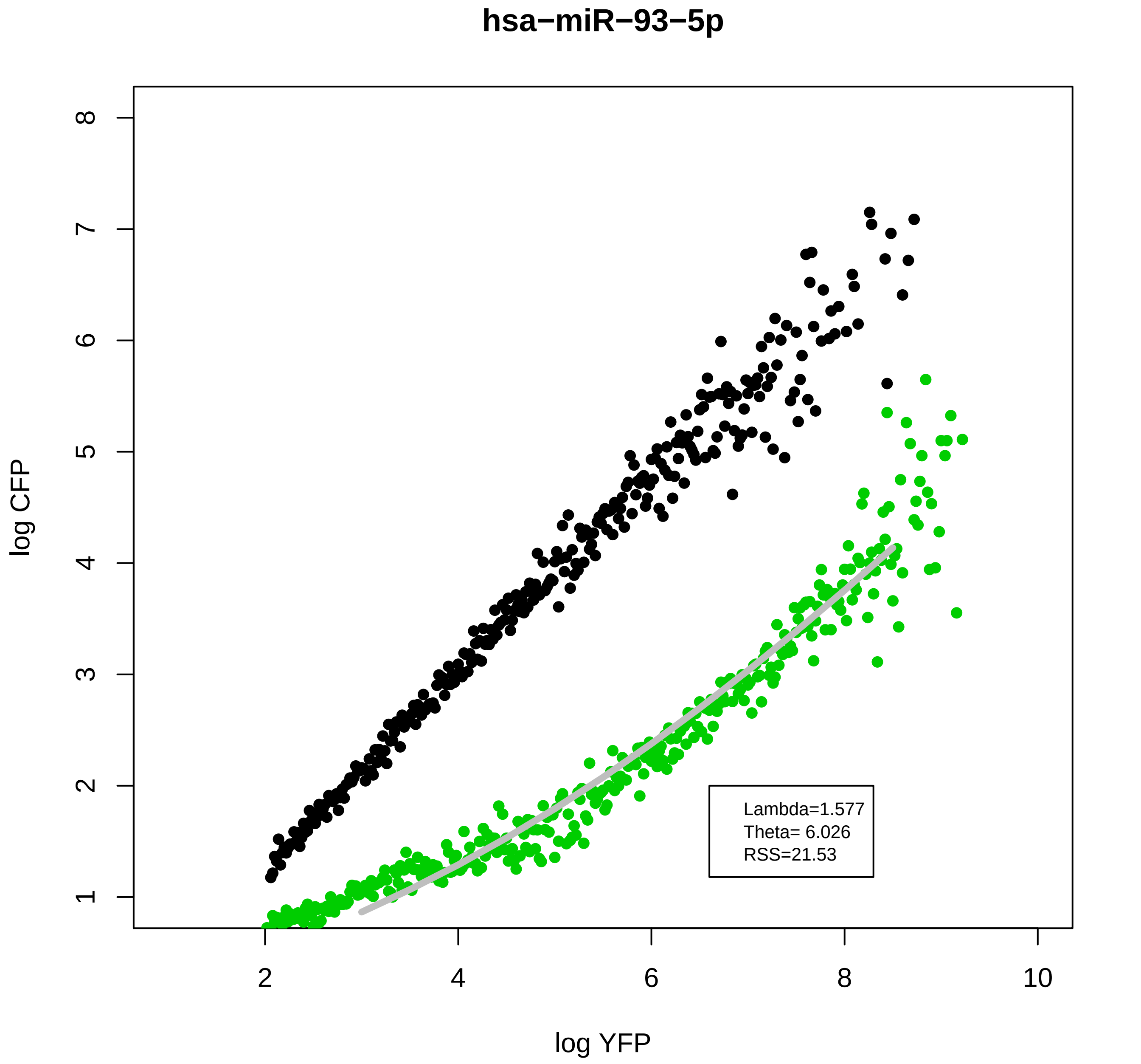

hsa−miR−93−5p
log CFP
log YFP
1
2
3
4
5
6
7
8
2
4
6
8
10
Lambda=1.577
Theta= 6.026
RSS=21.53

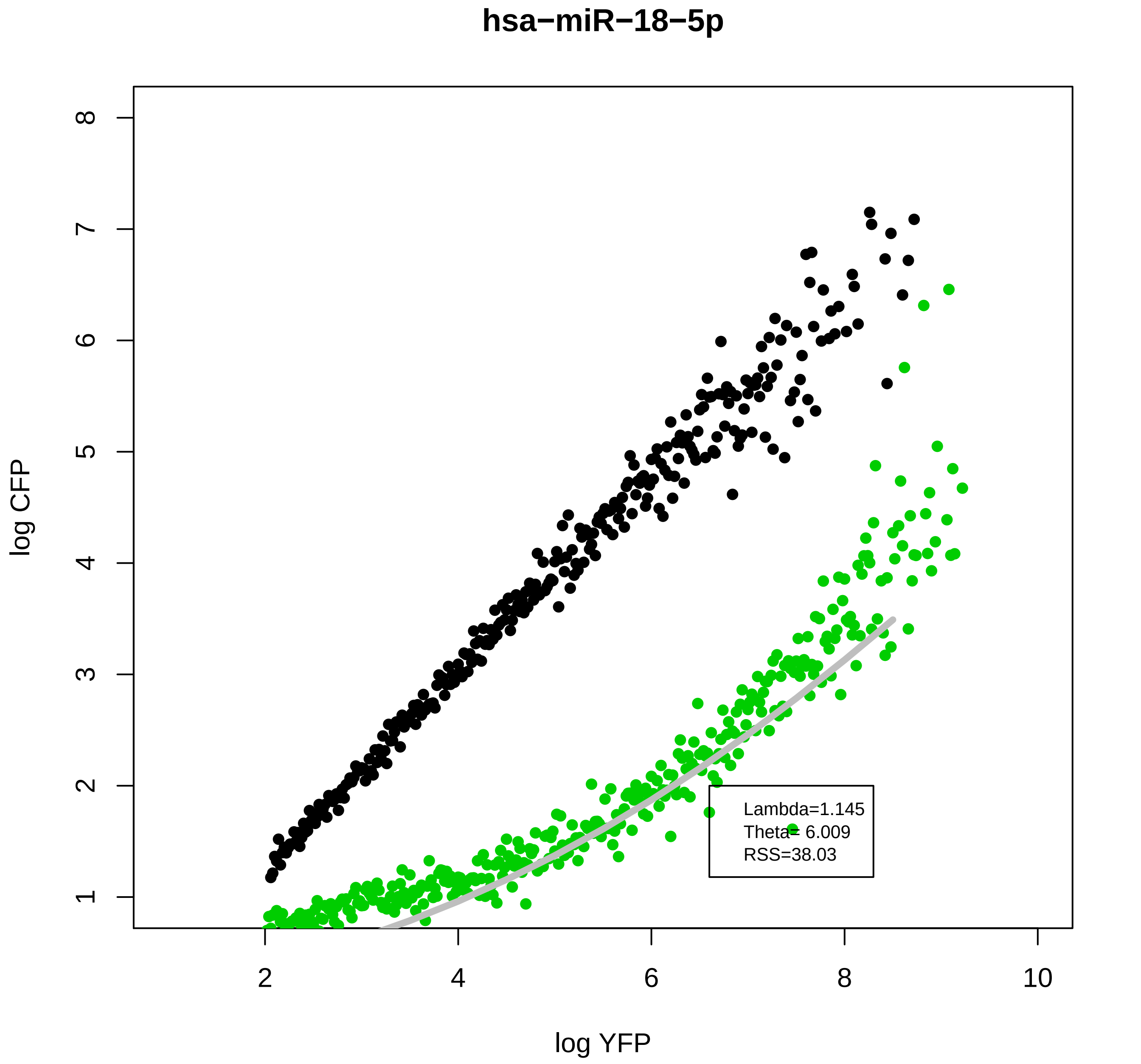

hsa−miR−18−5p
log CFP
log YFP
1
2
3
4
5
6
7
8
2
4
6
8
10
Lambda=1.145
Theta= 6.009
RSS=38.03

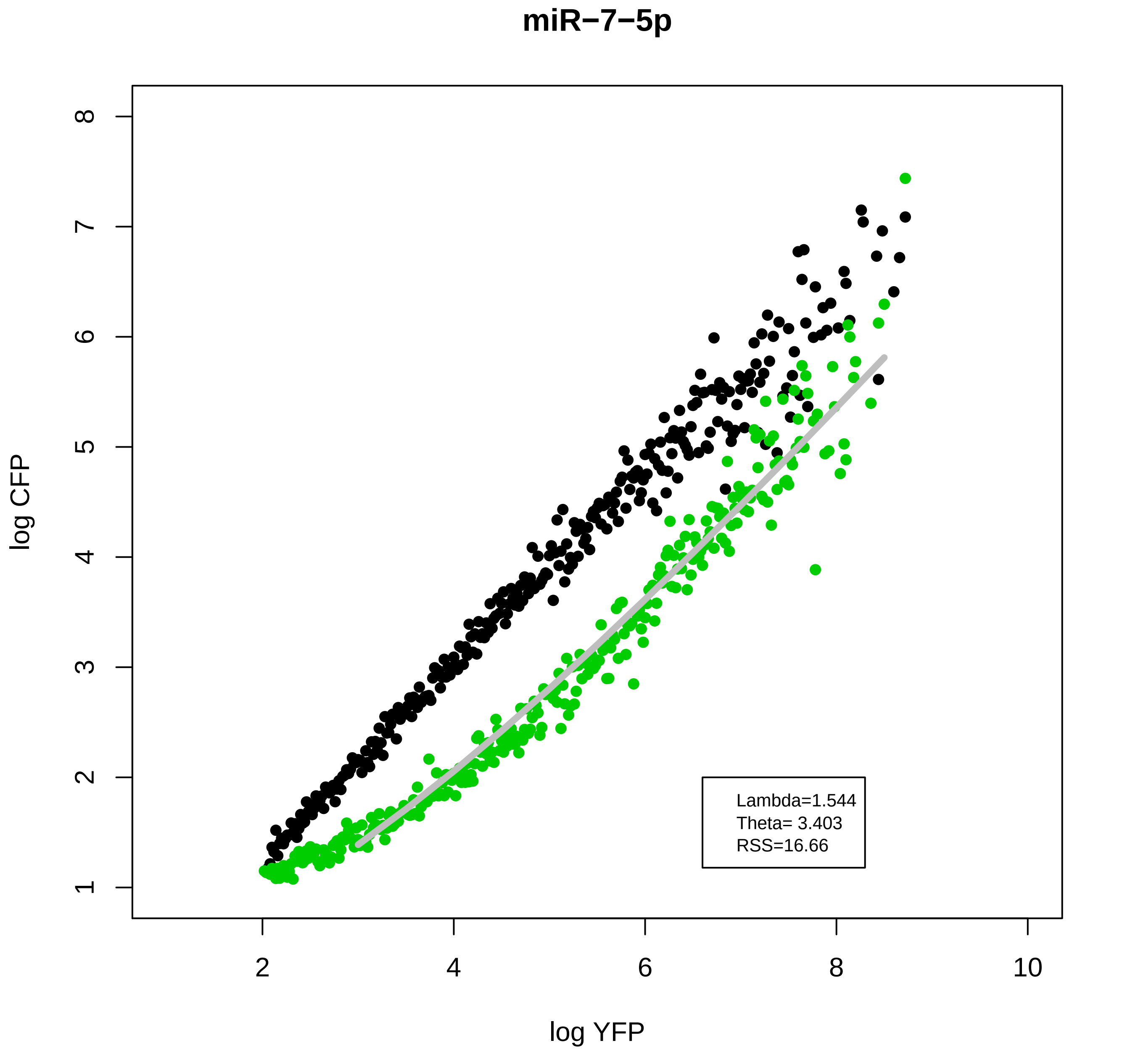

miR−7−5p
log CFP
log YFP
1
2
3
4
5
6
7
8
2
4
6
8
10
Lambda=1.544
Theta= 3.403
RSS=16.66

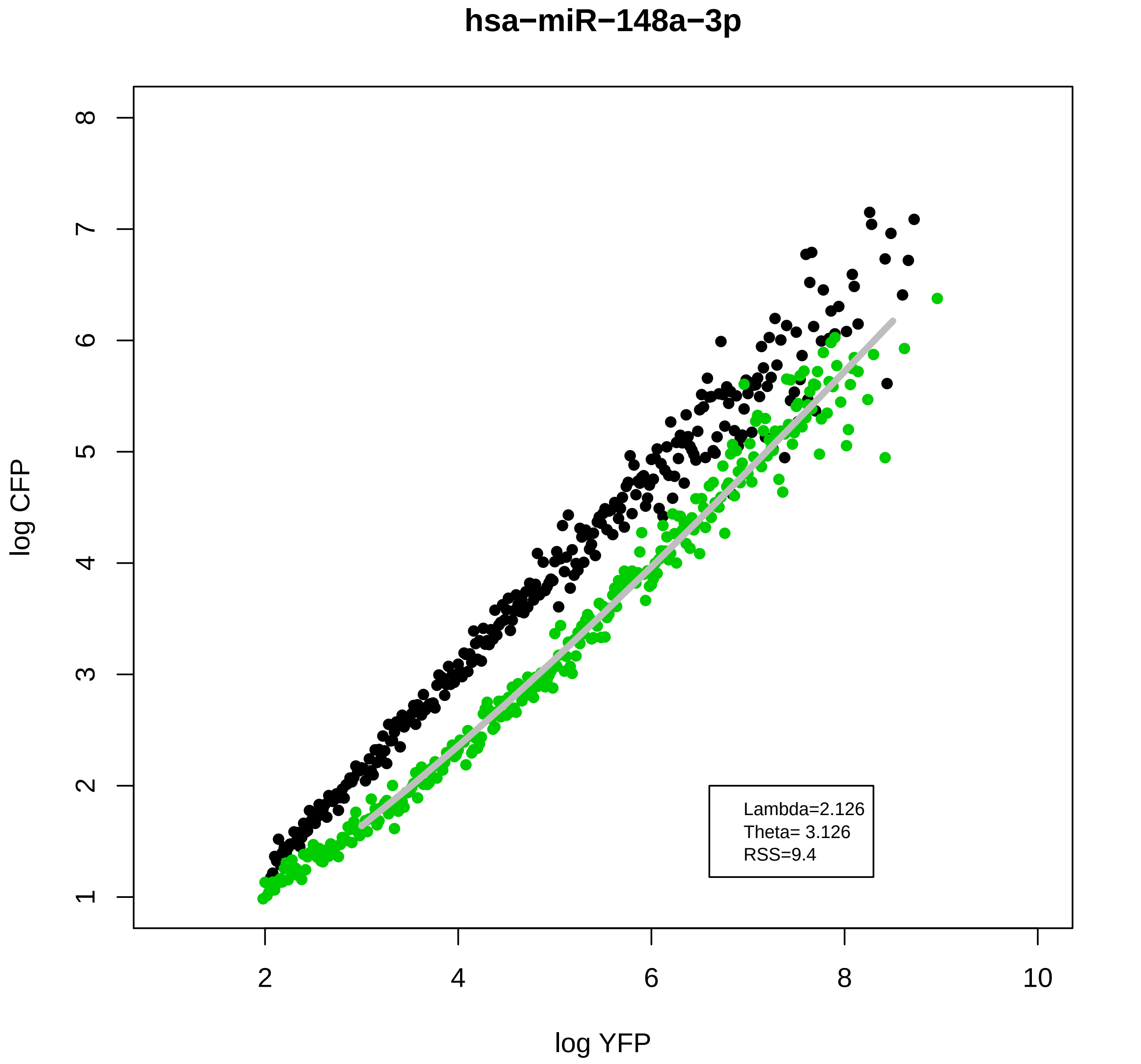
hsa−miR−148a−3p
log CFP
log YFP
1
2
3
4
5
6
7
8
2
4
6
8
10
Lambda=2.126
Theta= 3.126
RSS=9.4

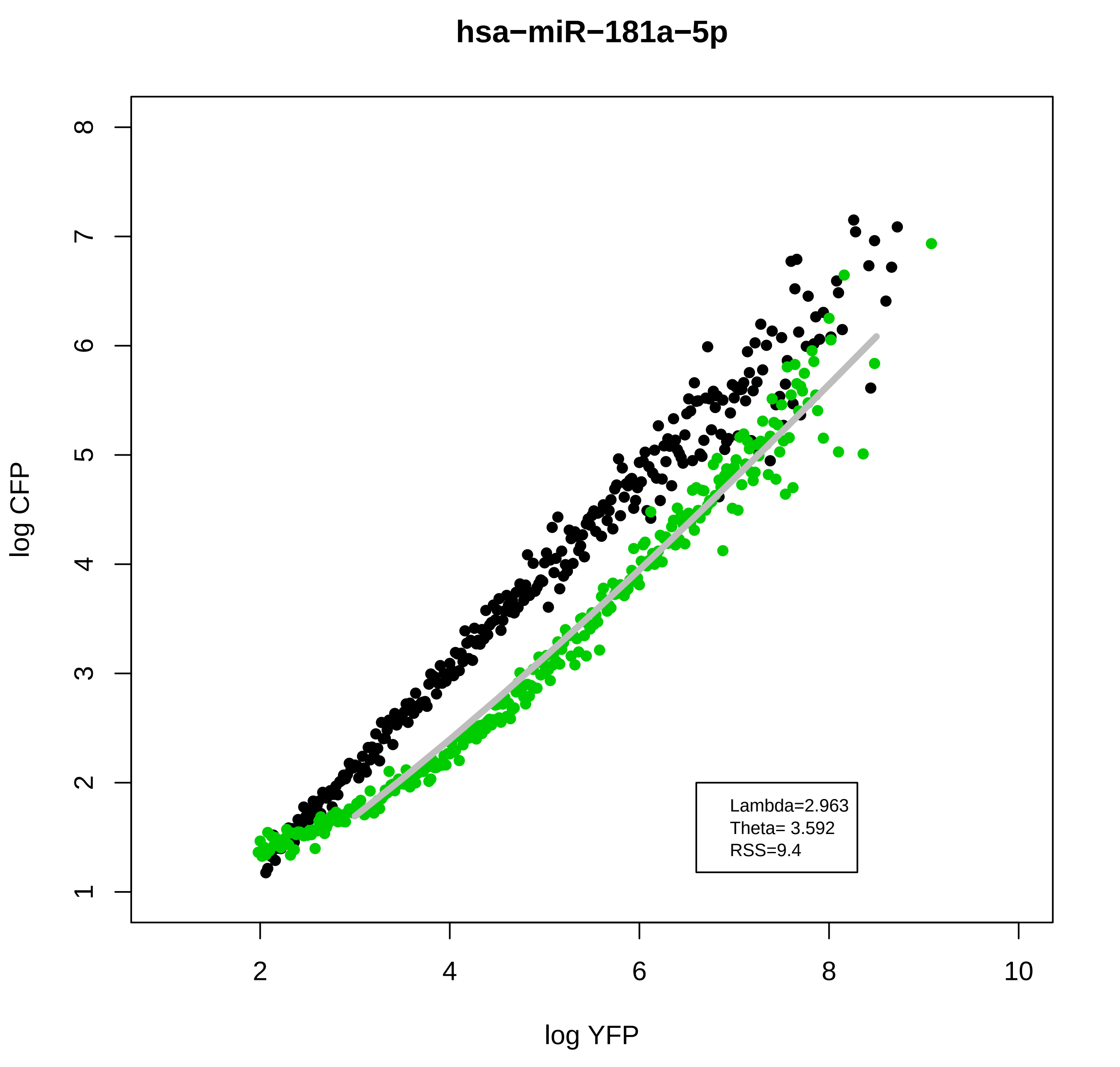
hsa−miR−181a−5p
log CFP
log YFP
1
2
3
4
5
6
7
8
2
4
6
8
10
Lambda=2.963
Theta= 3.592
RSS=9.4

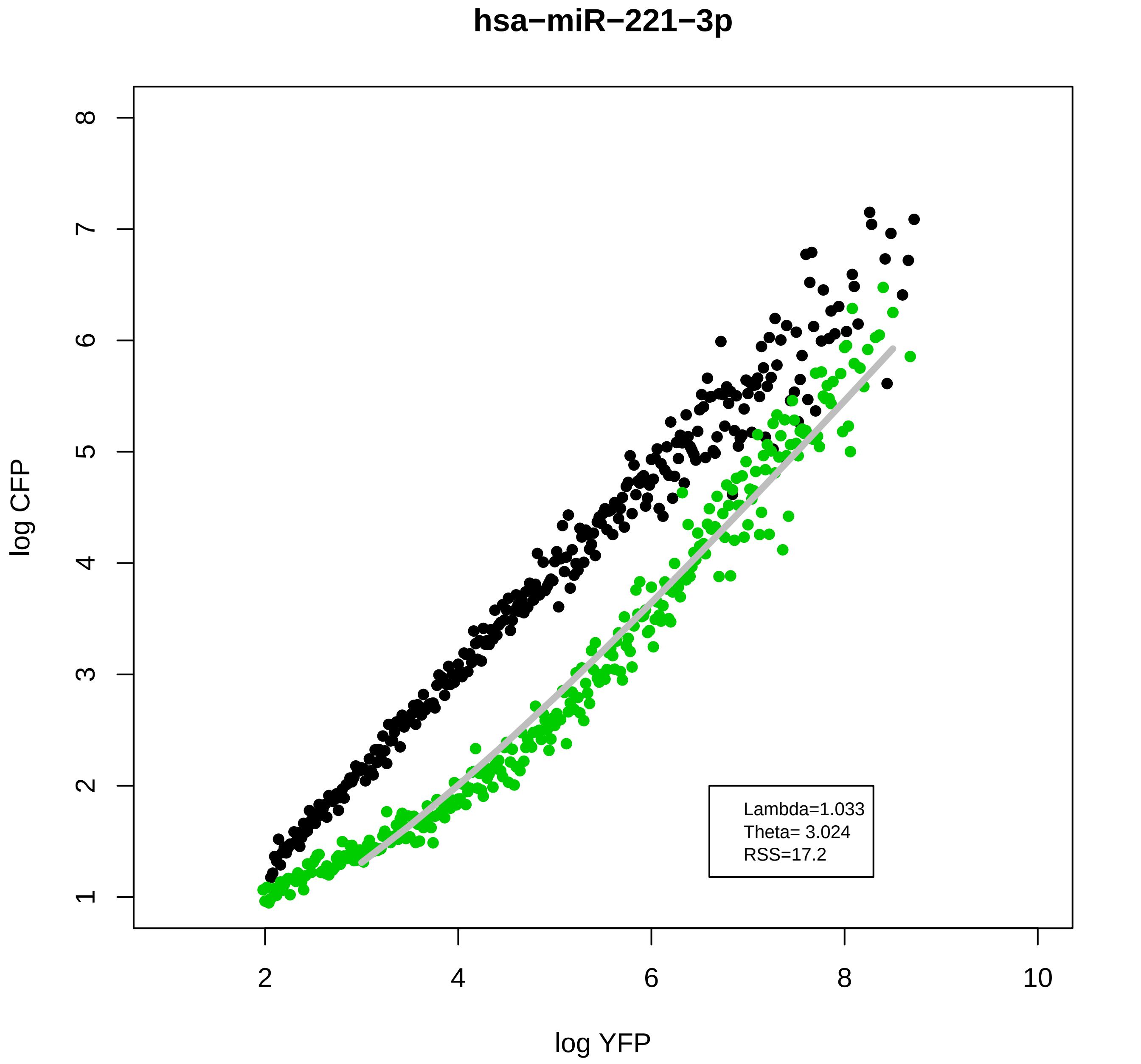

hsa−miR−221−3p
log CFP
log YFP
1
2
3
4
5
6
7
8
2
4
6
8
10
Lambda=1.033
Theta= 3.024
RSS=17.2

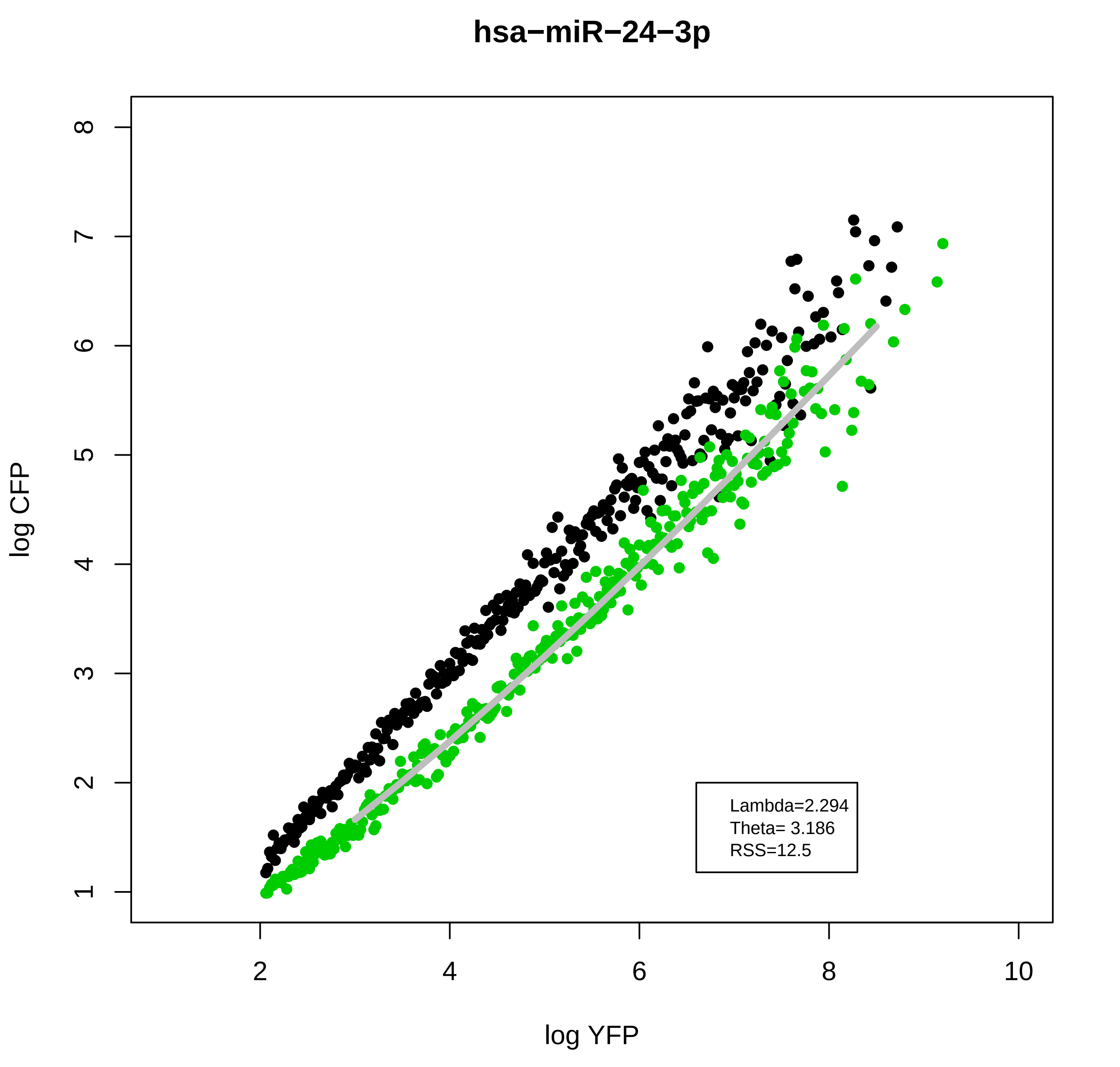
hsa−miR−24−3p
log CFP
log YFP
1
2
3
4
5
6
7
8
2
4
6
8
10
Lambda=2.294
Theta= 3.186
RSS=12.5

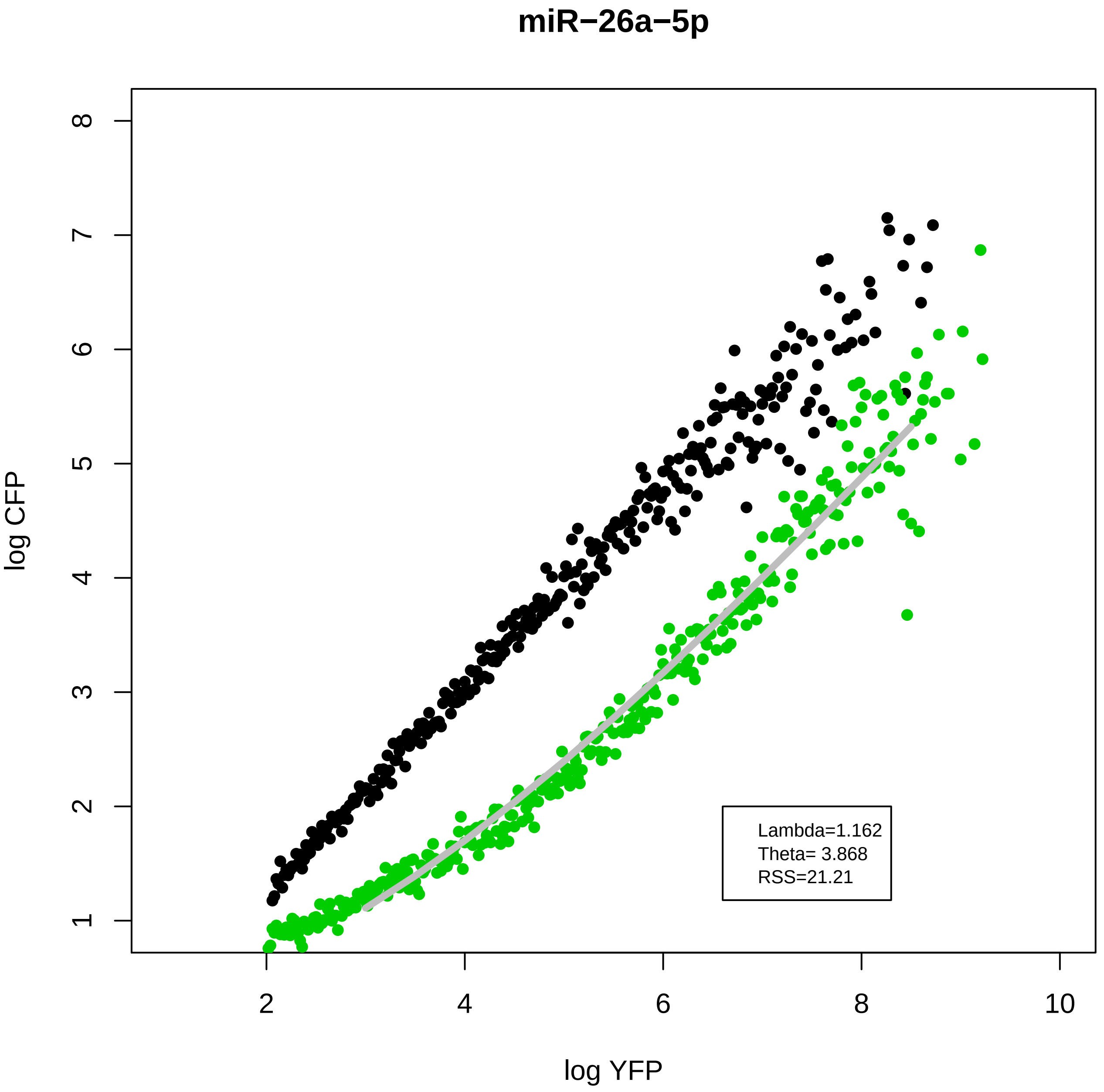

miR−26a−5p
log CFP
log YFP
1
2
3
4
5
6
7
8
2
4
6
8
10
Lambda=1.162
Theta= 3.868
RSS=21.21

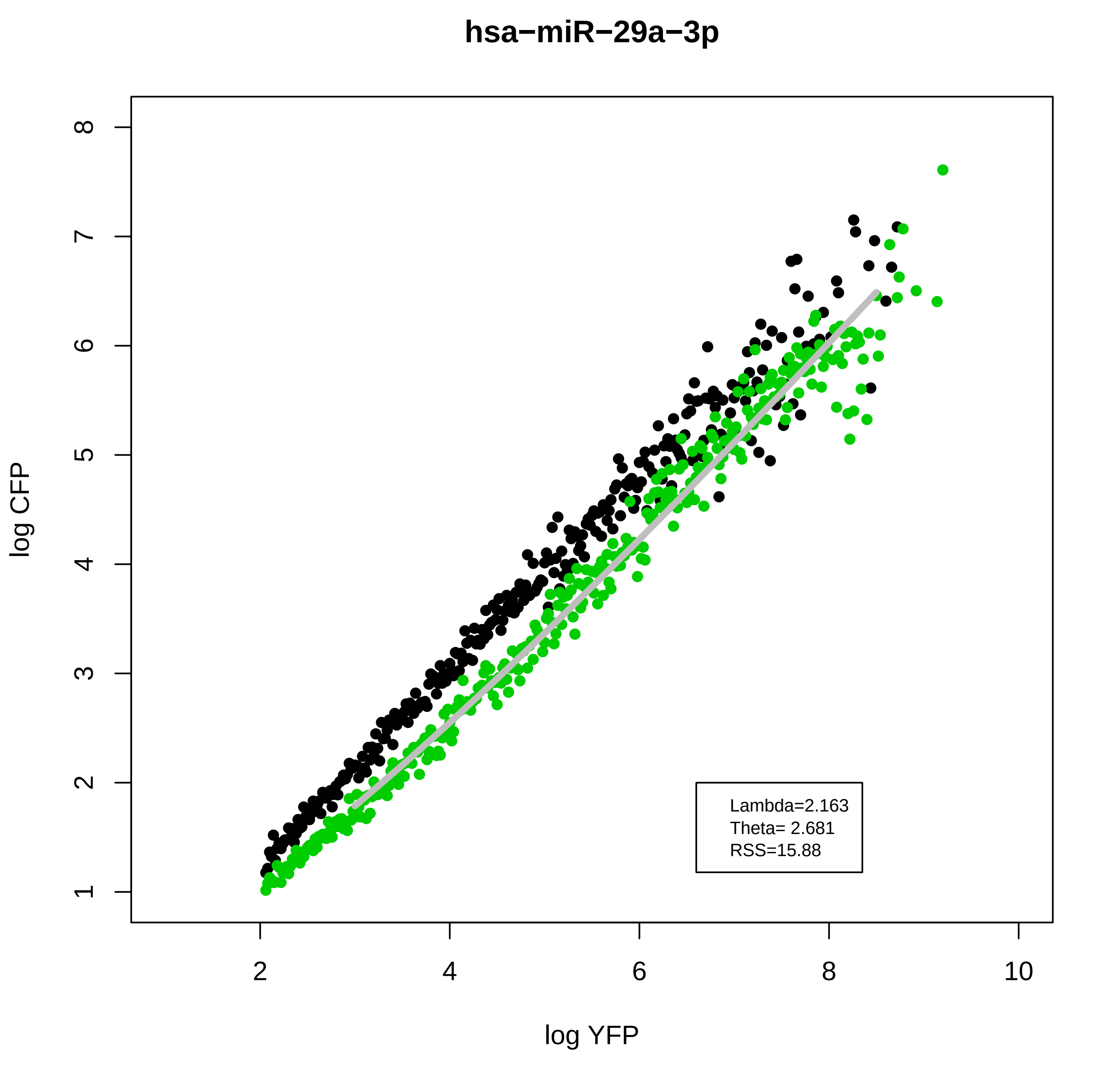

hsa−miR−29a−3p
log CFP
log YFP
1
2
3
4
5
6
7
8
2
4
6
8
10
Lambda=2.163
Theta= 2.681
RSS=15.88

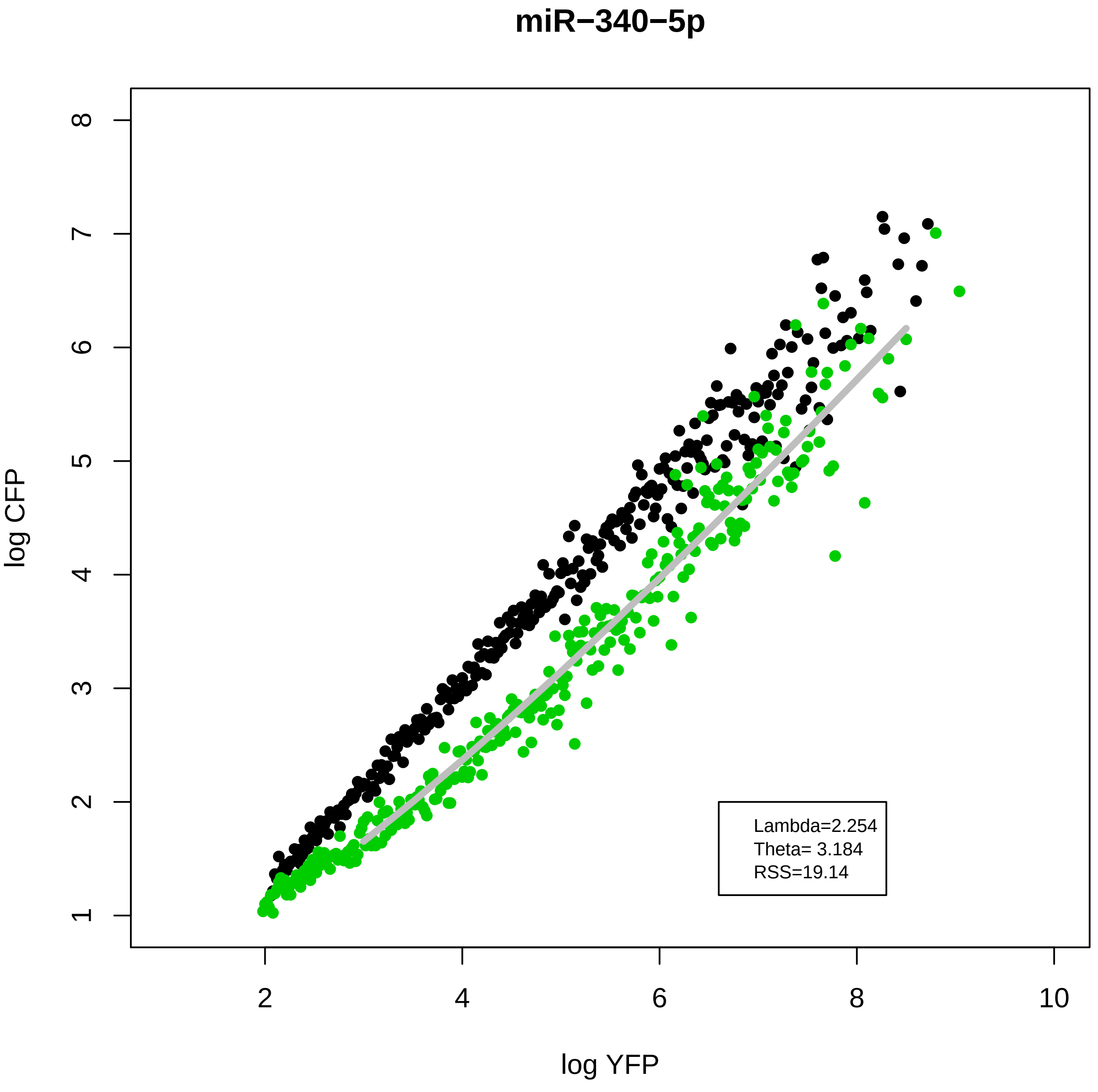

miR−340−5p
log CFP
log YFP
1
2
3
4
5
6
7
8
2
4
6
8
10
Lambda=2.254
Theta= 3.184
RSS=19.14

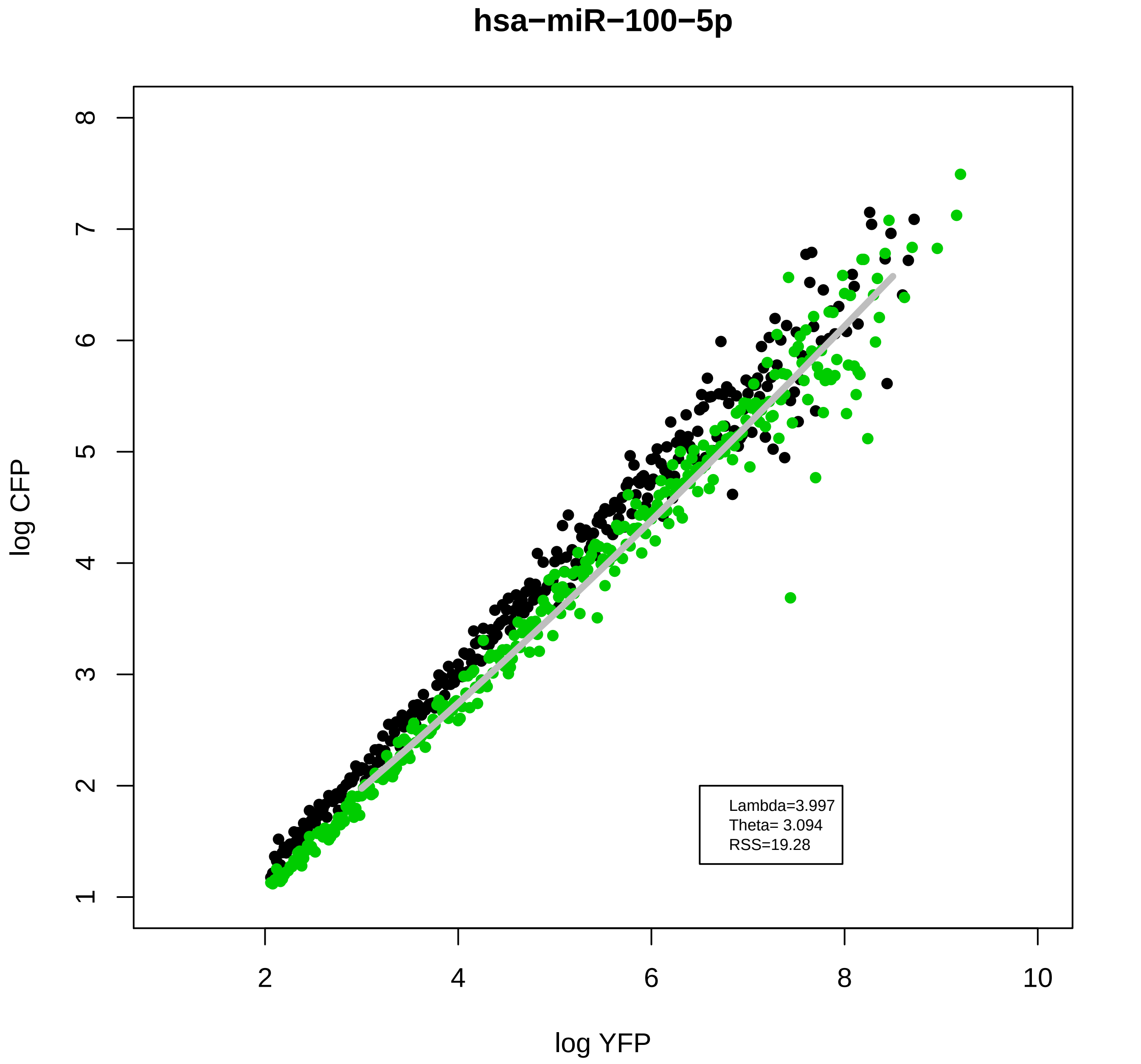

hsa−miR−100−5p
log CFP
log YFP
1
2
3
4
5
6
7
8
2
4
6
8
10
Lambda=3.997
Theta= 3.094
RSS=19.28

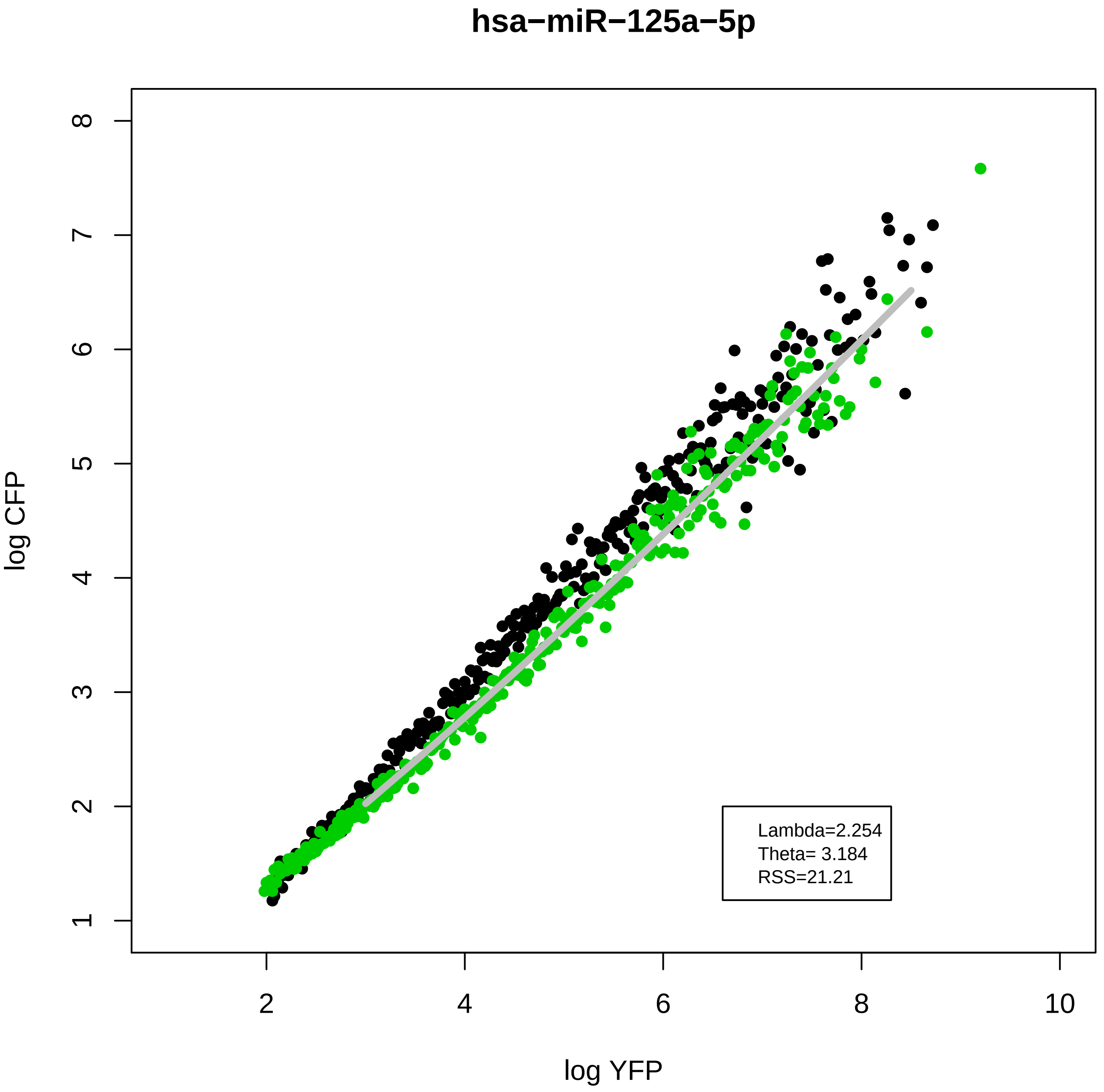
hsa−miR−125a−5p
log CFP
log YFP
1
2
3
4
5
6
7
8
2
4
6
8
10
Lambda=2.254
Theta= 3.184
RSS=21.21

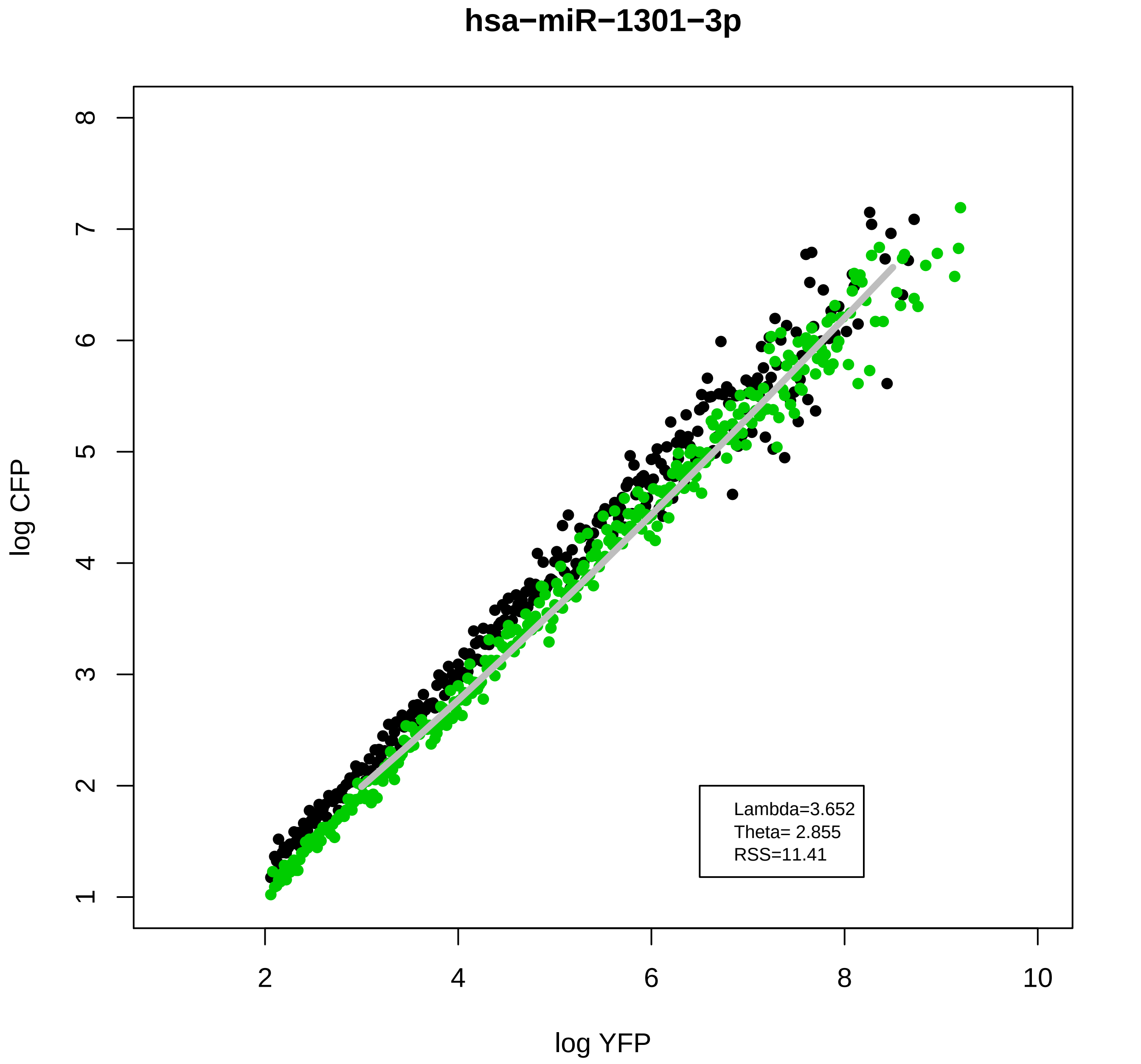

hsa−miR−1301−3p
log CFP
log YFP
1
2
3
4
5
6
7
8
2
4
6
8
10
Lambda=3.652
Theta= 2.855
RSS=11.41

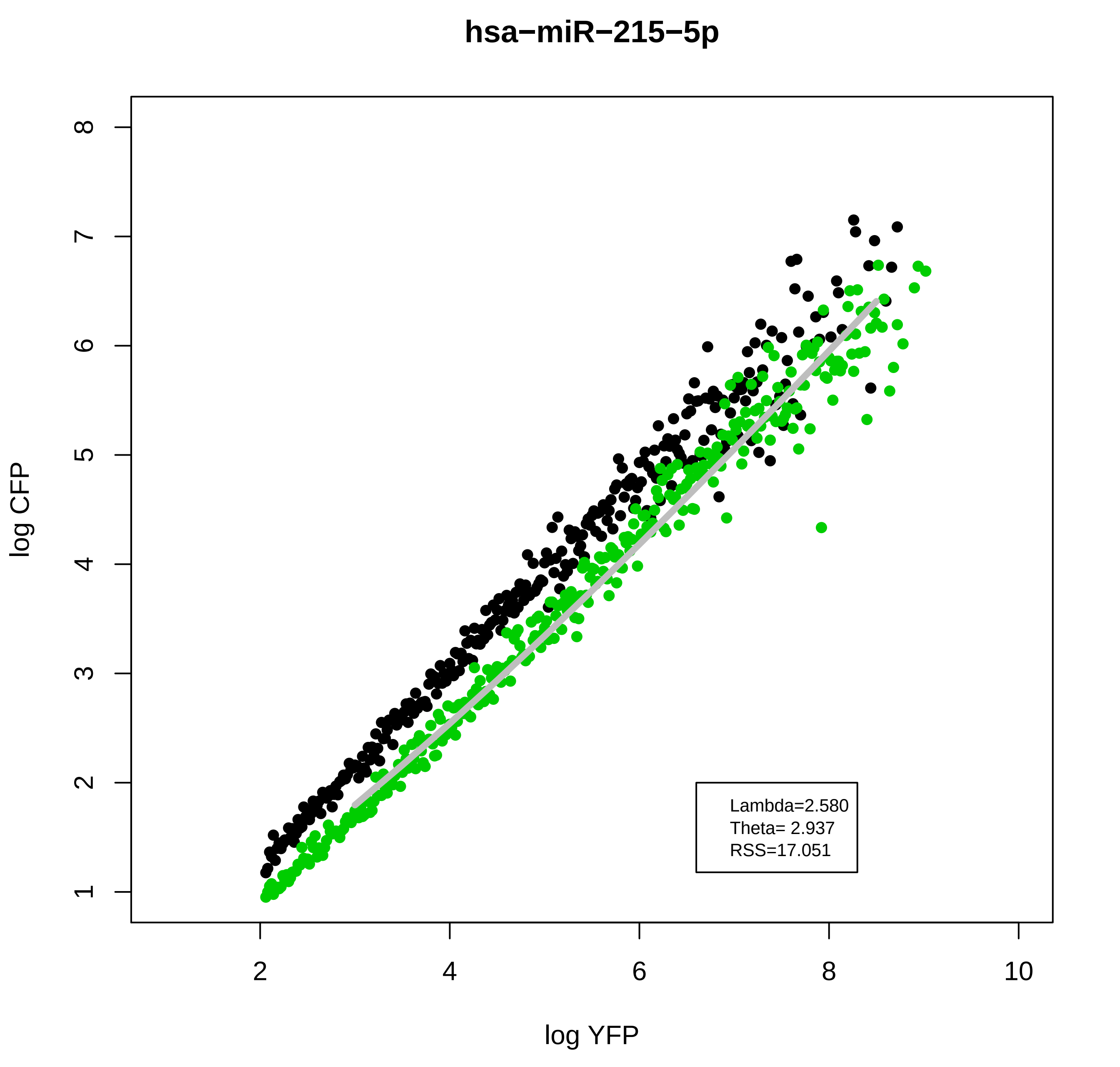

hsa−miR−215−5p
log CFP
log YFP
1
2
3
4
5
6
7
8
2
4
6
8
10
Lambda=2.580
Theta= 2.937
RSS=17.051

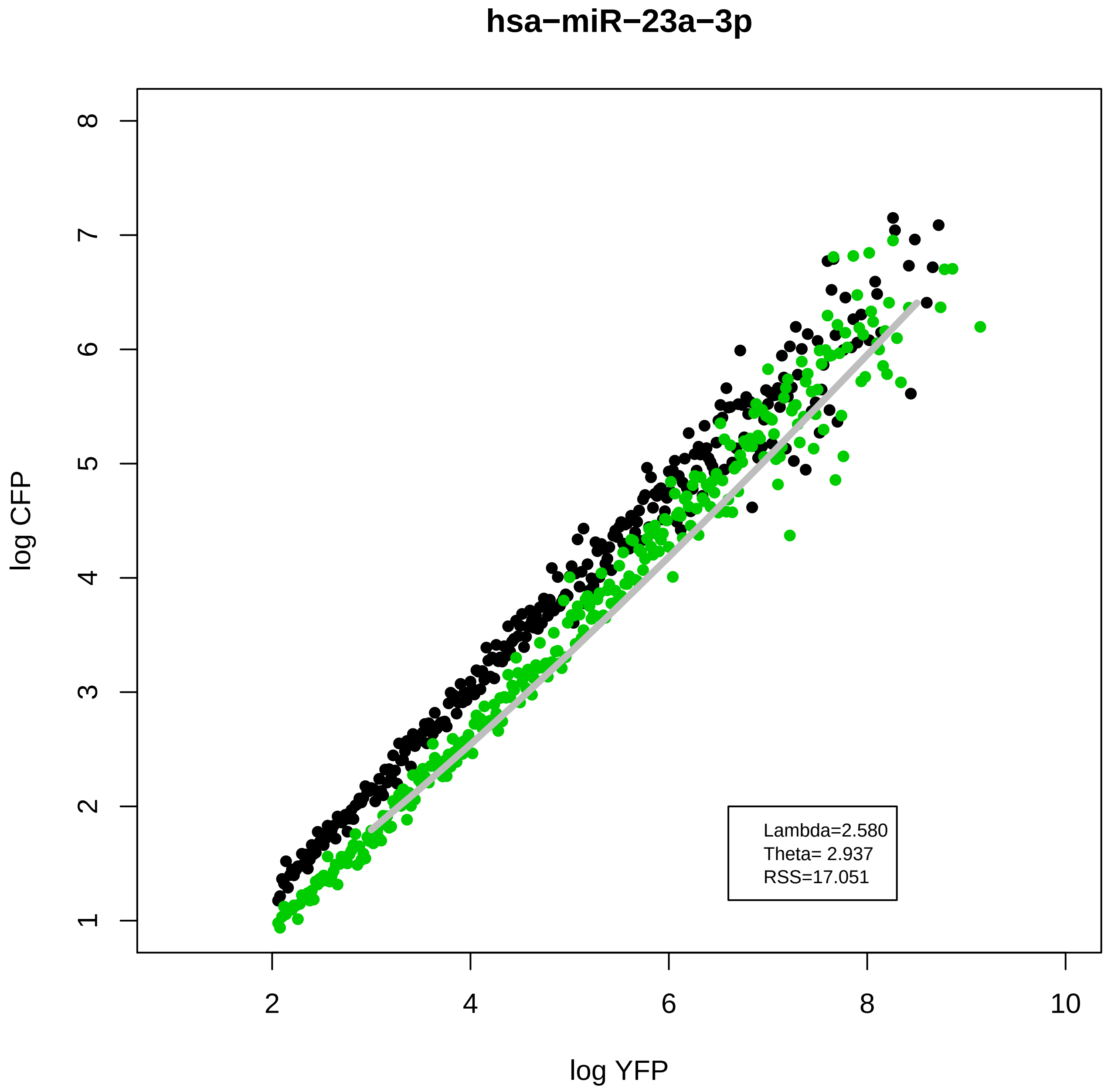

hsa−miR−23a−3p
log CFP
log YFP
1
2
3
4
5
6
7
8
2
4
6
8
10
Lambda=2.580
Theta= 2.937
RSS=17.051

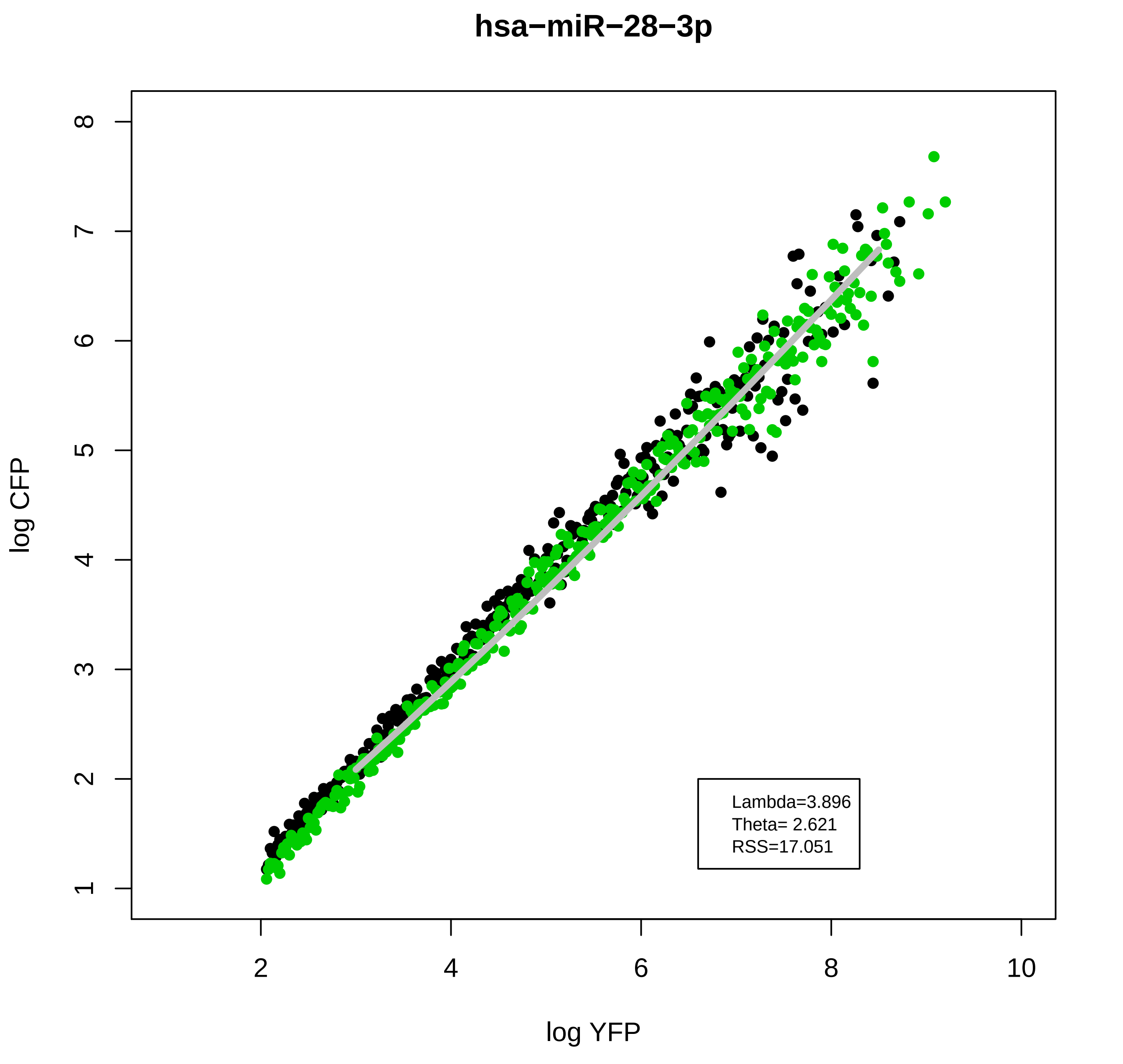

hsa−miR−28−3p
log CFP
log YFP
1
2
3
4
5
6
7
8
2
4
6
8
10
Lambda=3.896
Theta= 2.621
RSS=17.051

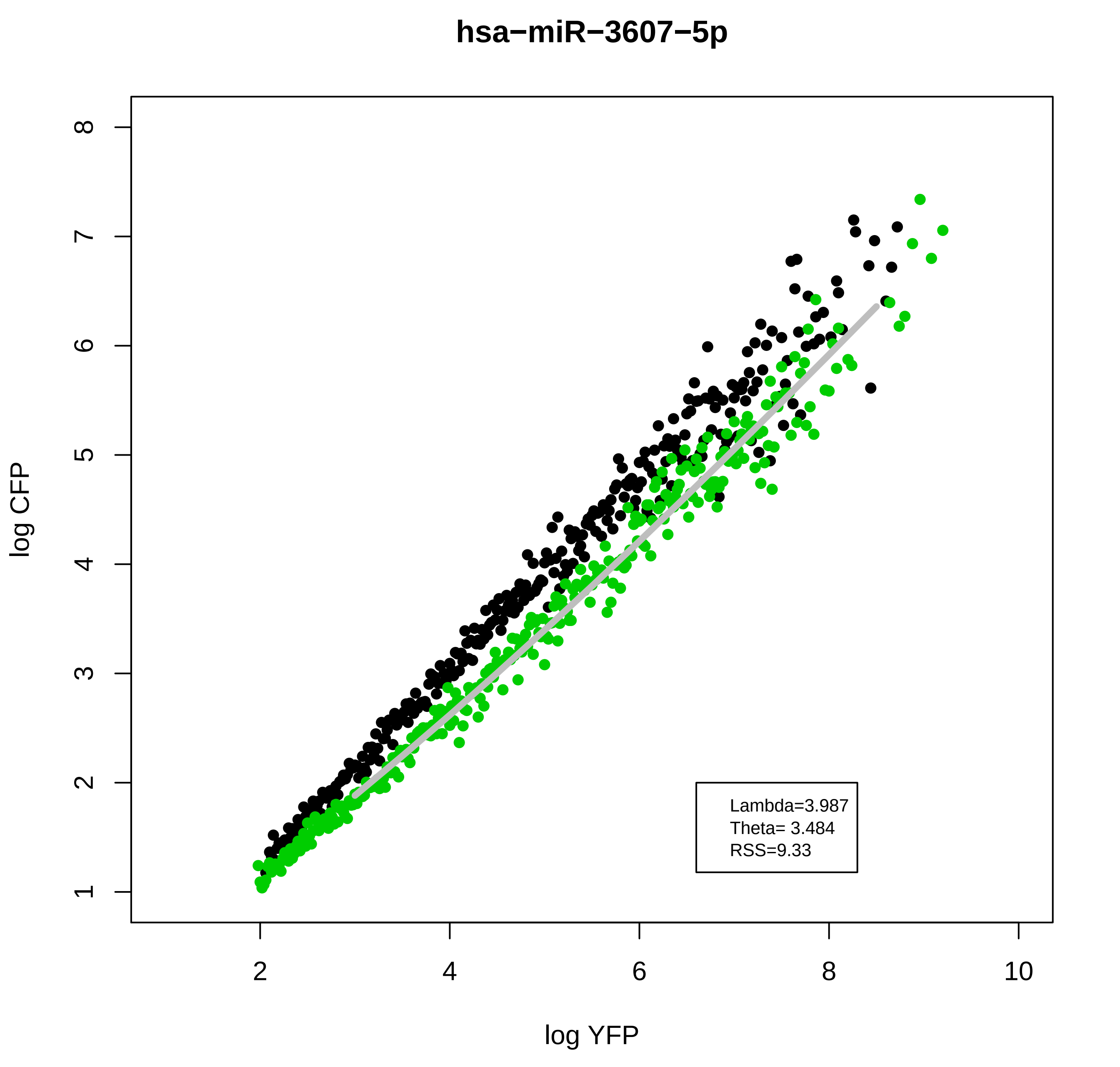
hsa−miR−3607−5p
log CFP
1
2
3
4
5
6
7
8
log YFP
2
4
6
8
10
Lambda=3.987
Theta= 3.484
RSS=9.33

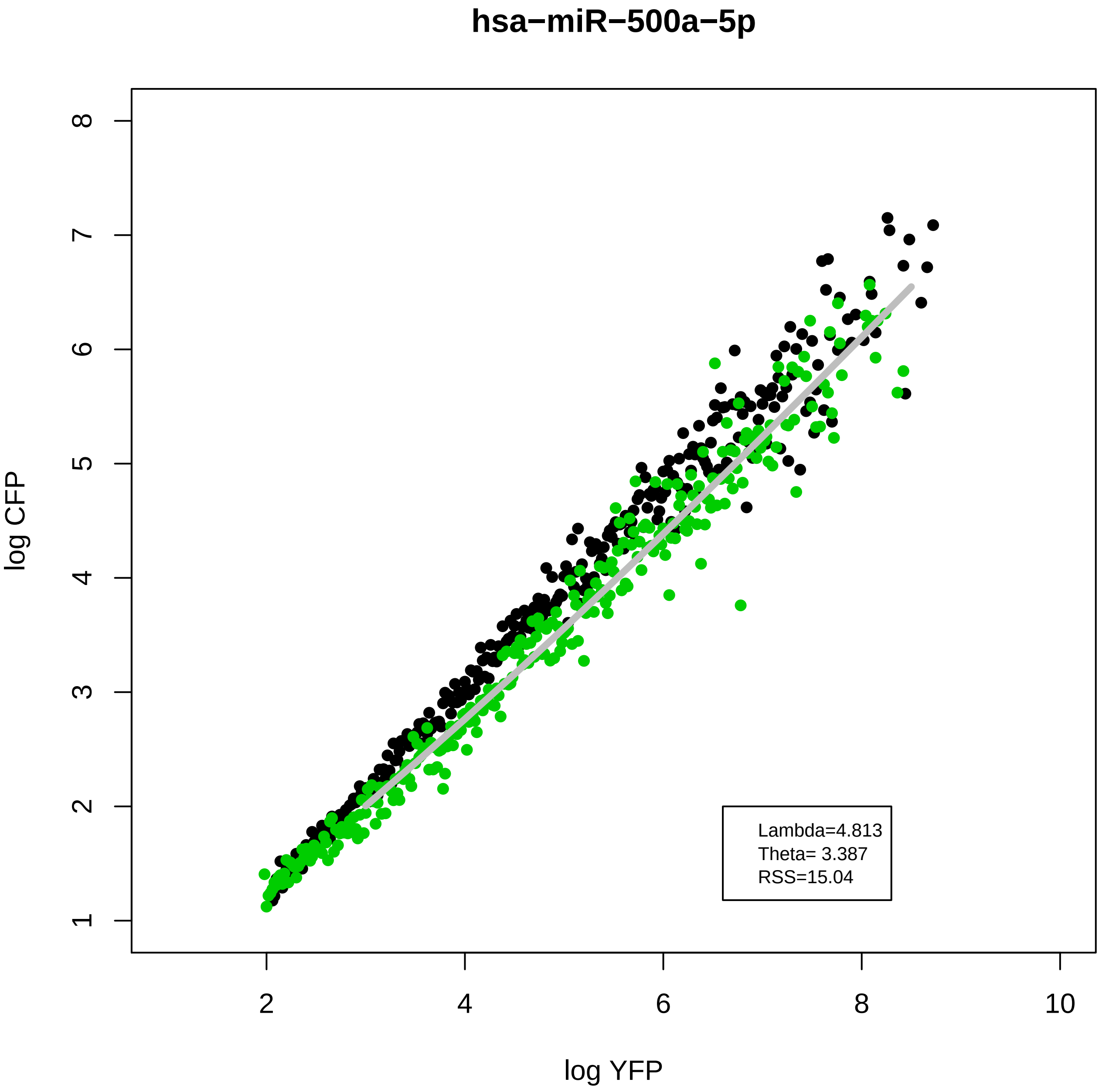

hsa−miR−500a−5p
Lambda=4.813
Theta= 3.387
RSS=15.04
log CFP
log YFP
1
2
3
4
5
6
7
8
2
4
6
8
10

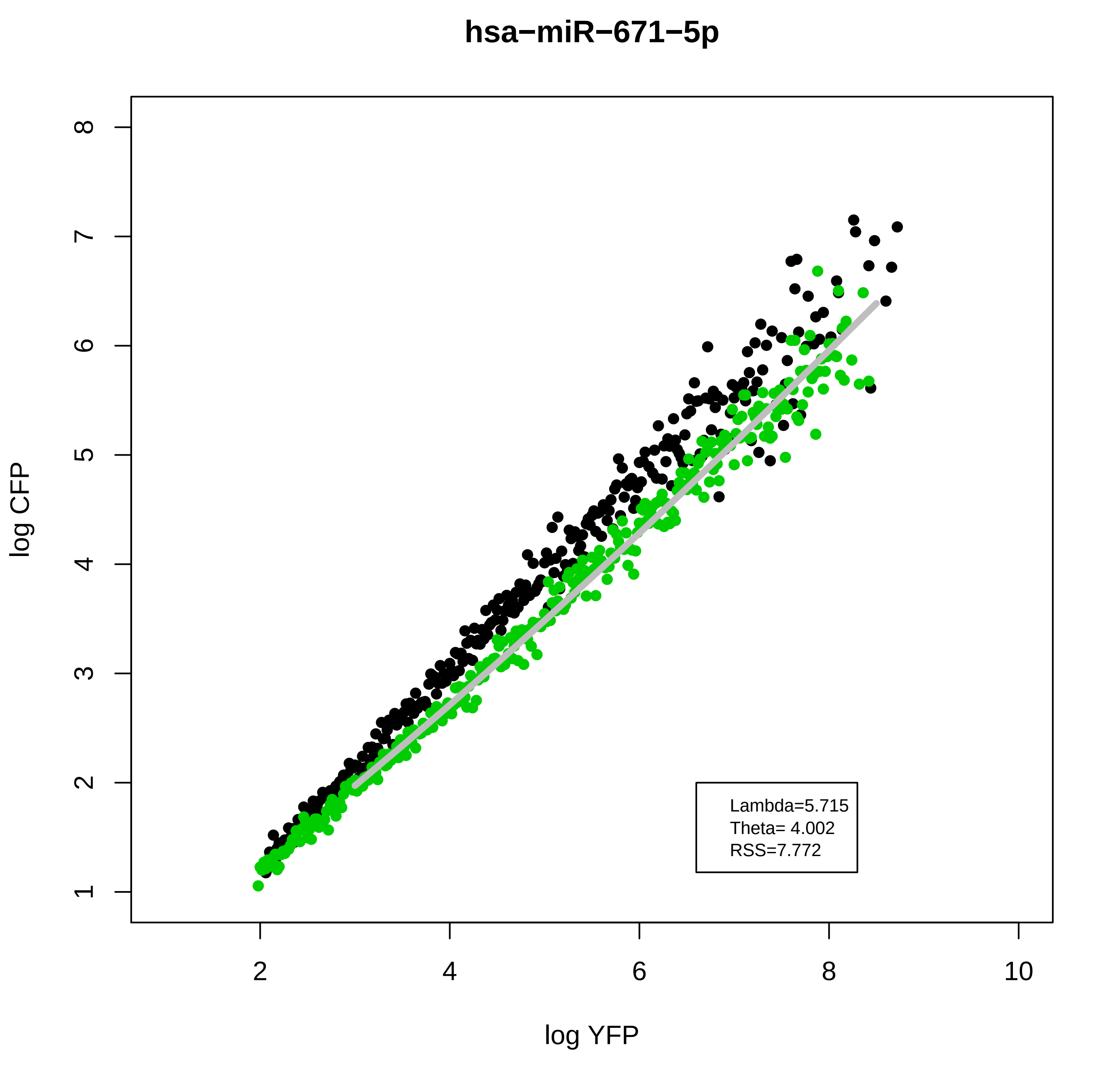
hsa−miR−671−5p
log CFP
log YFP
Lambda=5.715
Theta= 4.002
RSS=7.772
1
2
3
4
5
6
7
8
2
4
6
8
10

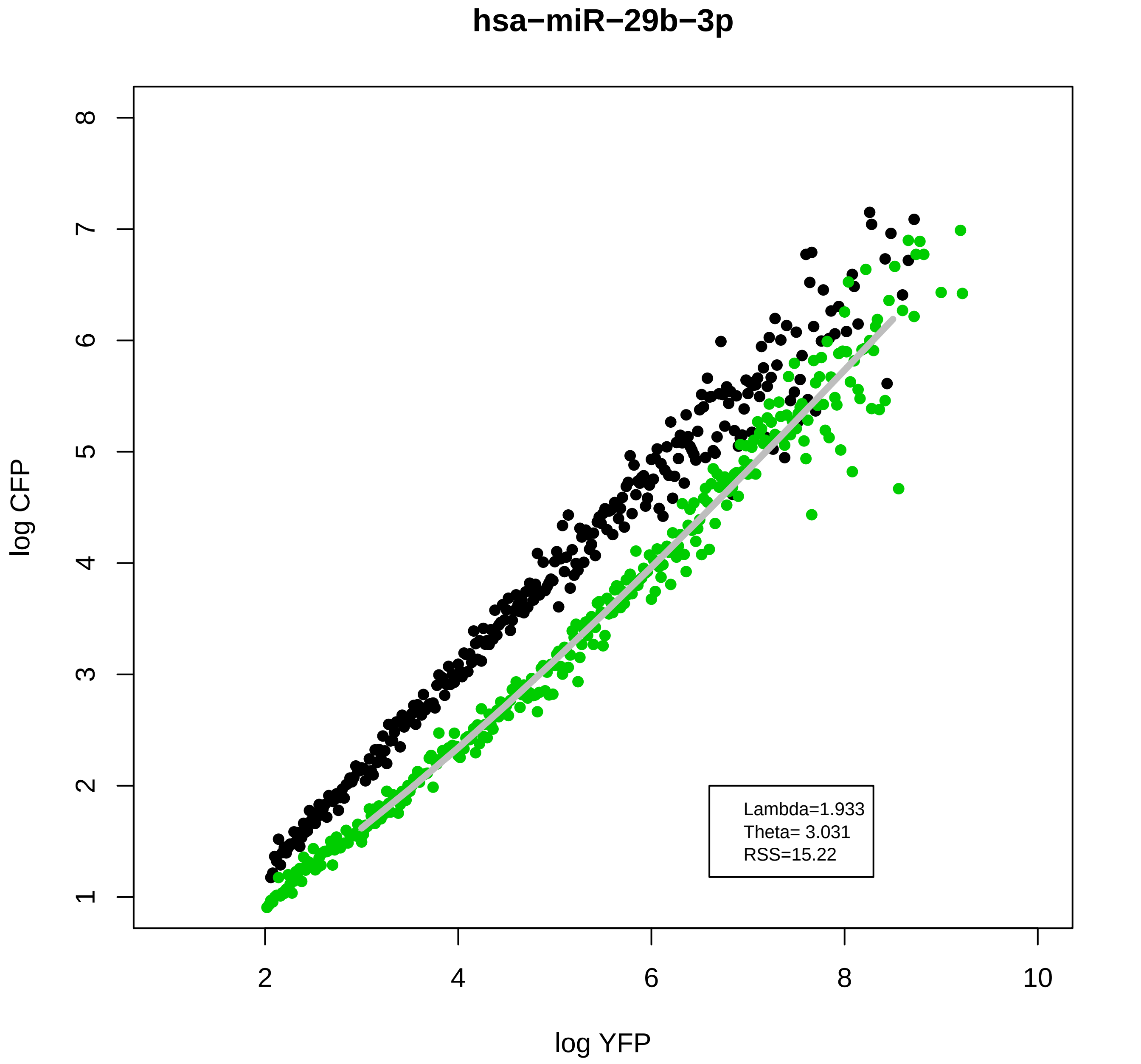

hsa−miR−29b−3p
log CFP
log YFP
1
2
3
4
5
6
7
8
2
4
6
8
10
Lambda=1.933
Theta= 3.031
RSS=15.22

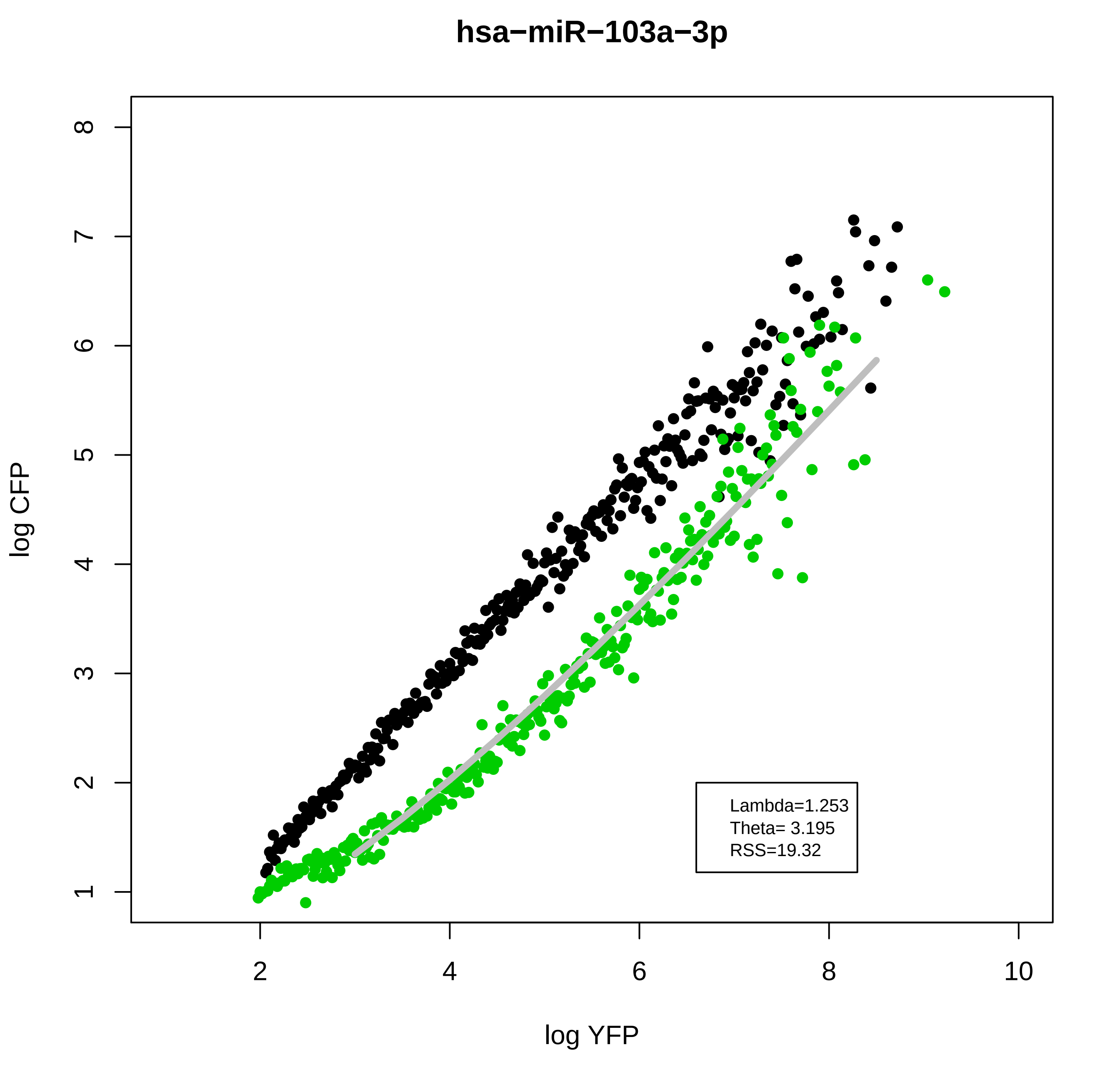
hsa−miR−103a−3p
log CFP
log YFP
1
2
3
4
5
6
7
8
2
4
6
8
10
Lambda=1.253
Theta= 3.195
RSS=19.32

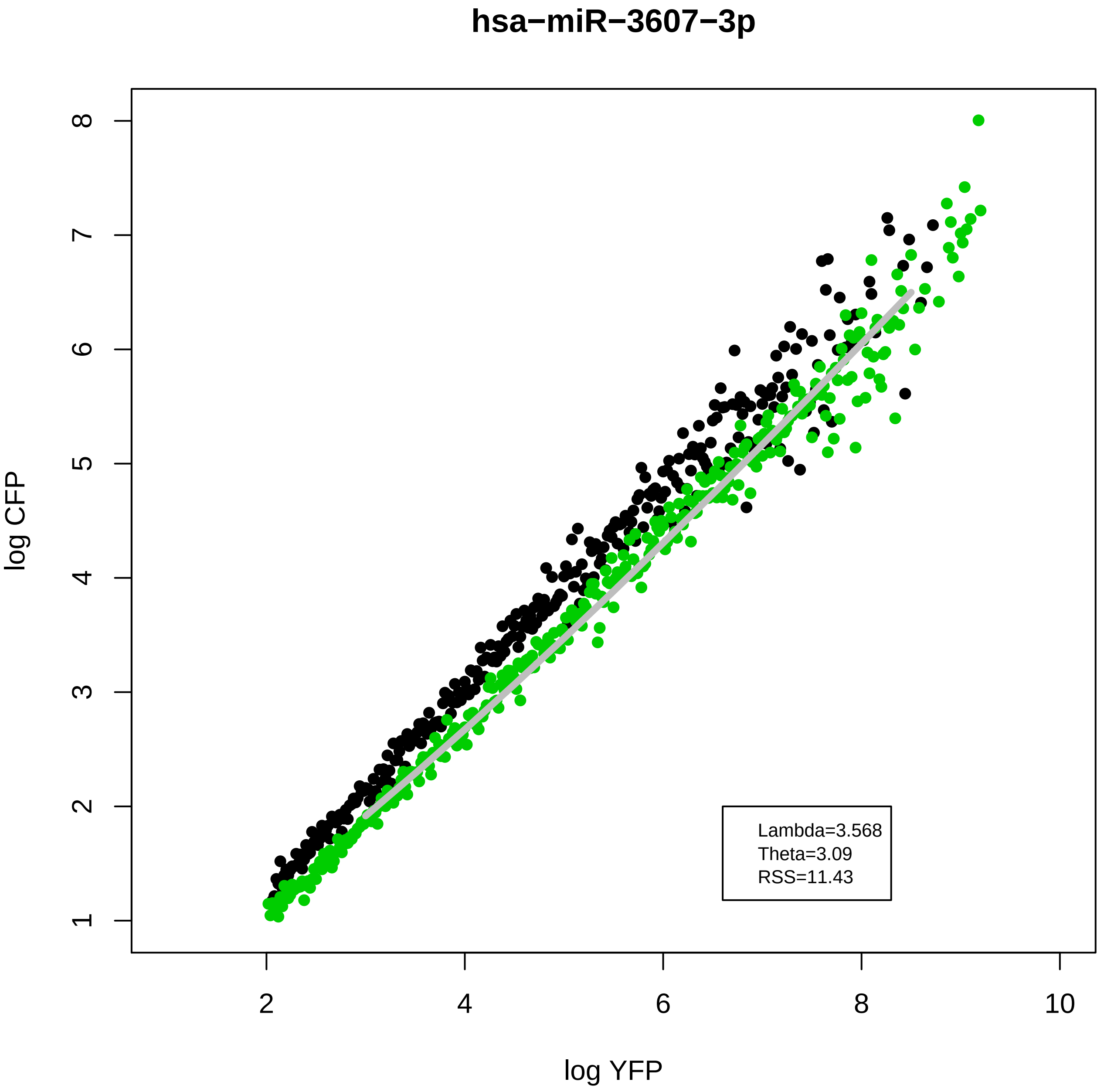
hsa−miR−3607−3p
log CFP
log YFP
1
2
3
4
5
6
7
8
2
4
6
8
10
Lambda=3.568
Theta=3.09
RSS=11.43

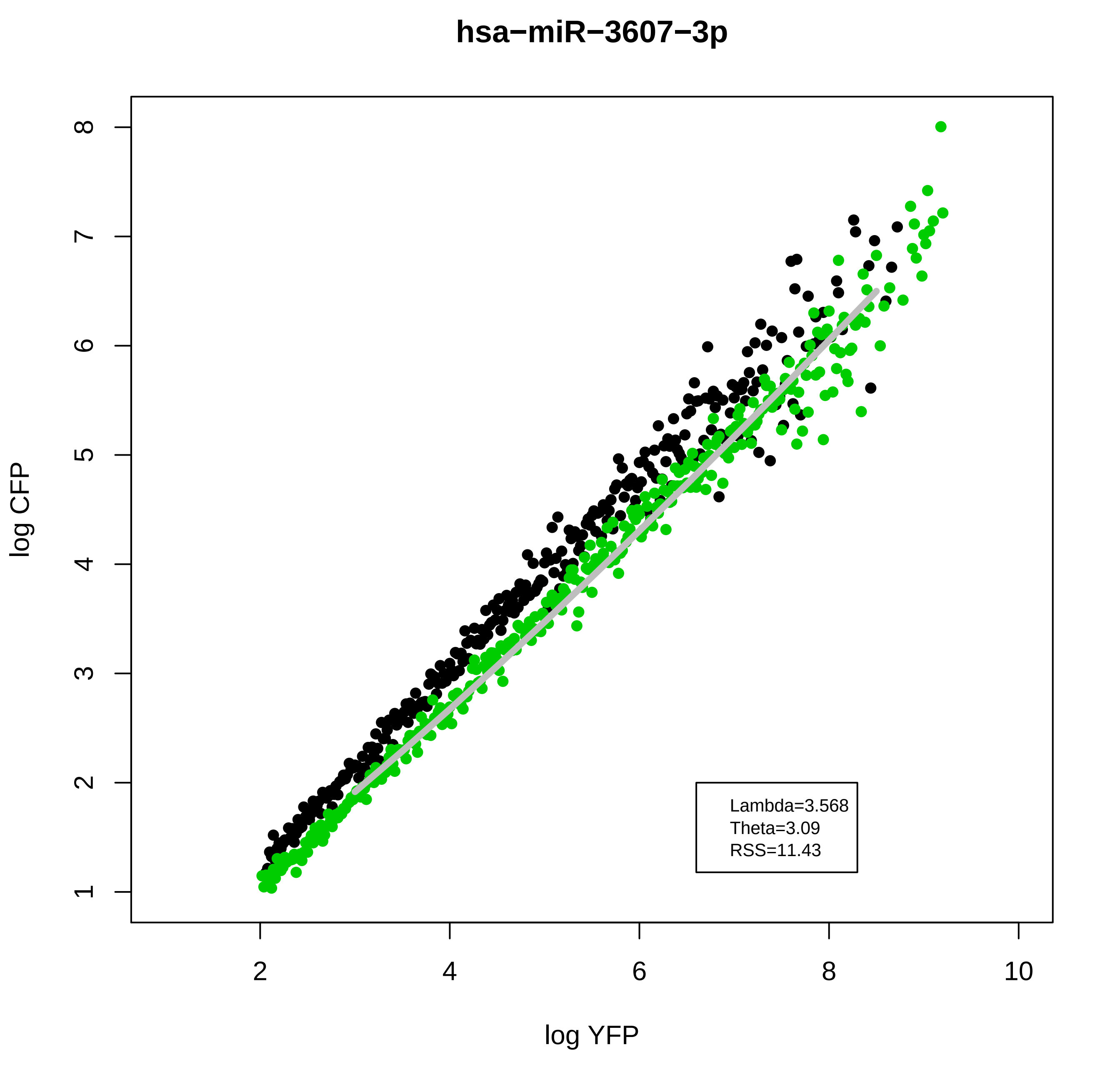
hsa−miR−3607−3p
log CFP
log YFP
1
2
3
4
5
6
7
8
2
4
6
8
10
Lambda=3.568
Theta=3.09
RSS=11.43

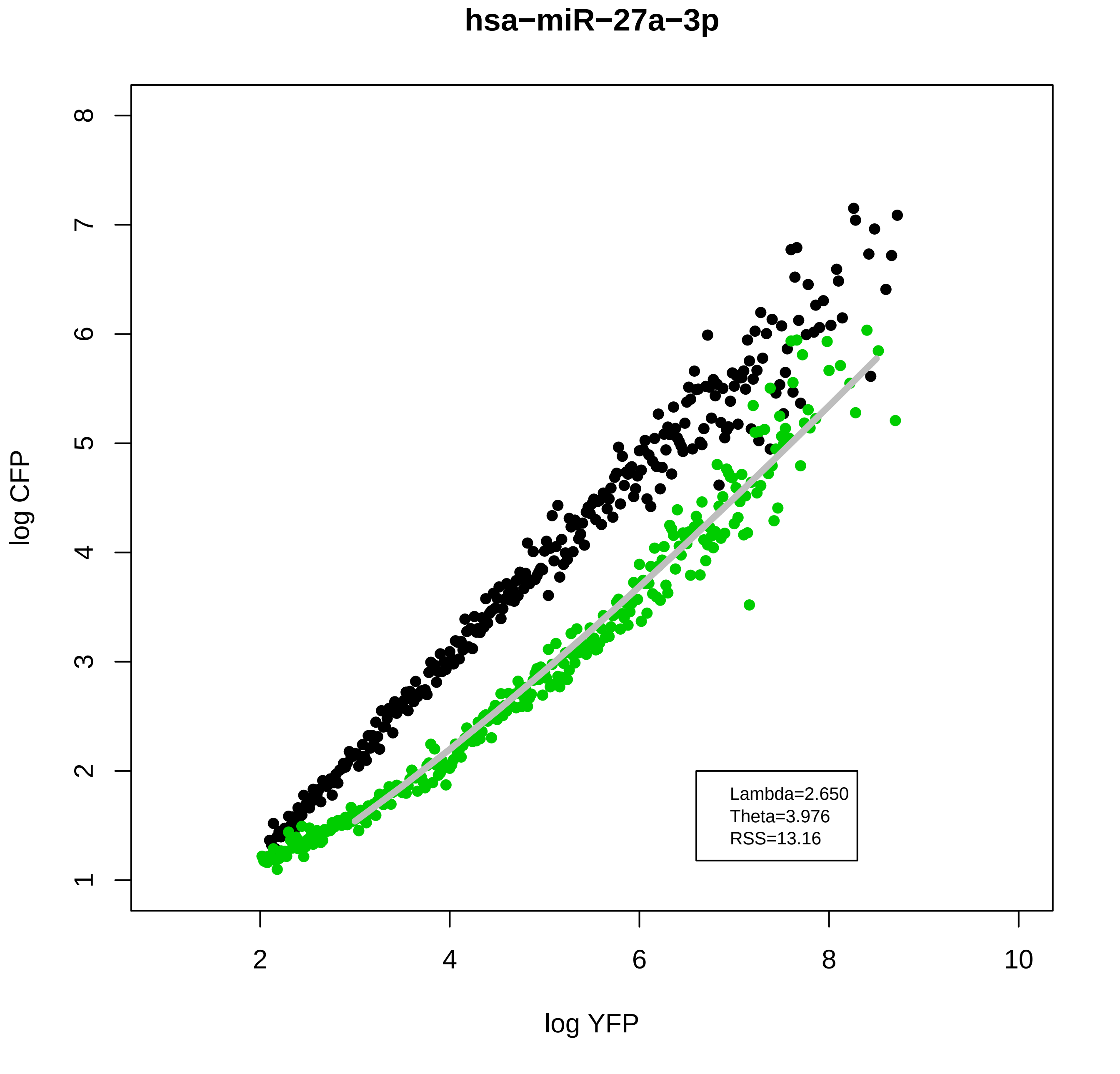
hsa−miR−27a−3p
log CFP
log YFP
1
2
3
4
5
6
7
8
2
4
6
8
10
Lambda=2.650
Theta=3.976
RSS=13.16

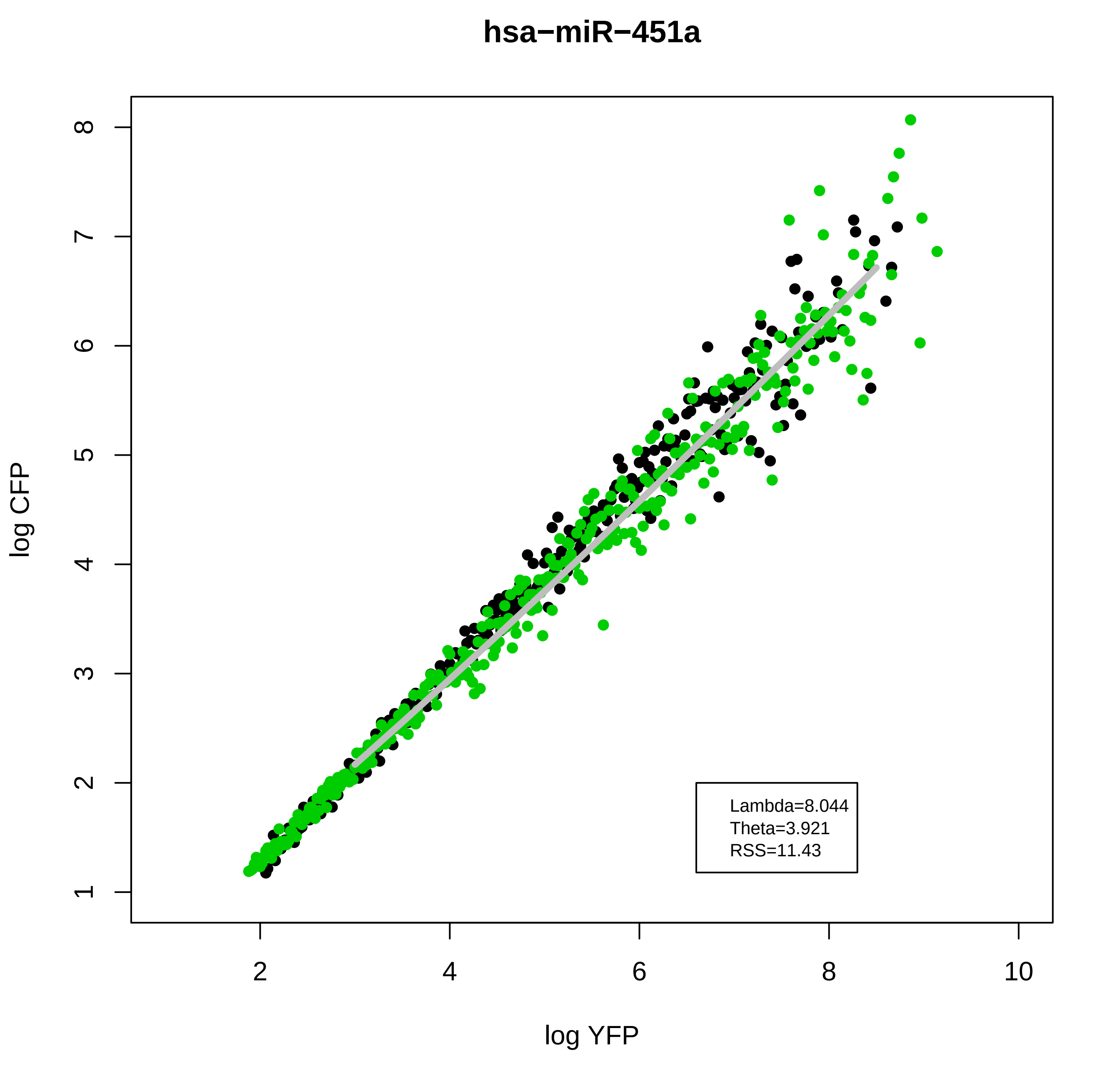
hsa−miR−451a
log CFP
log YFP
1
2
3
4
5
6
7
8
2
4
6
8
10
Lambda=8.044
Theta=3.921
RSS=11.43
